# Acidity and the multiphase chemistry of atmospheric aqueous particles and clouds

**DOI:** 10.5194/acp-21-13483-2021

**Published:** 2021-09-10

**Authors:** Andreas Tilgner, Thomas Schaefer, Becky Alexander, Mary Barth, Jeffrey L. Collett, Kathleen M. Fahey, Athanasios Nenes, Havala O. T. Pye, Hartmut Herrmann, V. Faye McNeill

**Affiliations:** 1 Atmospheric Chemistry Department (ACD), Leibniz Institute for Tropospheric Research (TROPOS), Leipzig 04318, Germany; 2 Department of Atmospheric Science, University of Washington, Seattle, WA 98195, USA; 3 Atmospheric Chemistry Observation & Modeling Laboratory, National Center for Atmospheric Research, Boulder, CO 80307, USA; 4 Department of Atmospheric Science, Colorado State University, Fort Collins, CO 80523, USA; 5 Office of Research and Development, U.S. Environmental Protection Agency, Research Triangle Park, Durham, NC 27711, USA; 6 School of Architecture, Civil and Environmental Engineering, École Polytechnique Fédérale de Lausanne, Lausanne 1015, Switzerland; 7 Institute for Chemical Engineering Sciences, Foundation for Research and Technology Hellas, Patras 26504, Greece; 8 Department of Chemical Engineering, Columbia University, New York, NY 10027, USA; 9 Department of Earth and Environmental Sciences, Columbia University, New York, NY 10027, USA

## Abstract

The acidity of aqueous atmospheric solutions is a key parameter driving both the partitioning of semi-volatile acidic and basic trace gases and their aqueous-phase chemistry. In addition, the acidity of atmospheric aqueous phases, e.g., deliquesced aerosol particles, cloud, and fog droplets, is also dictated by aqueous-phase chemistry. These feedbacks between acidity and chemistry have crucial implications for the tropospheric lifetime of air pollutants, atmospheric composition, deposition to terrestrial and oceanic ecosystems, visibility, climate, and human health. Atmospheric research has made substantial progress in understanding feedbacks between acidity and multiphase chemistry during recent decades. This paper reviews the current state of knowledge on these feedbacks with a focus on aerosol and cloud systems, which involve both inorganic and organic aqueous-phase chemistry. Here, we describe the impacts of acidity on the phase partitioning of acidic and basic gases and buffering phenomena. Next, we review feedbacks of different acidity regimes on key chemical reaction mechanisms and kinetics, as well as uncertainties and chemical subsystems with incomplete information.

Finally, we discuss atmospheric implications and highlight the need for future investigations, particularly with respect to reducing emissions of key acid precursors in a changing world, and the need for advancements in field and laboratory measurements and model tools.

## 1 Introduction

**Figure 1.**
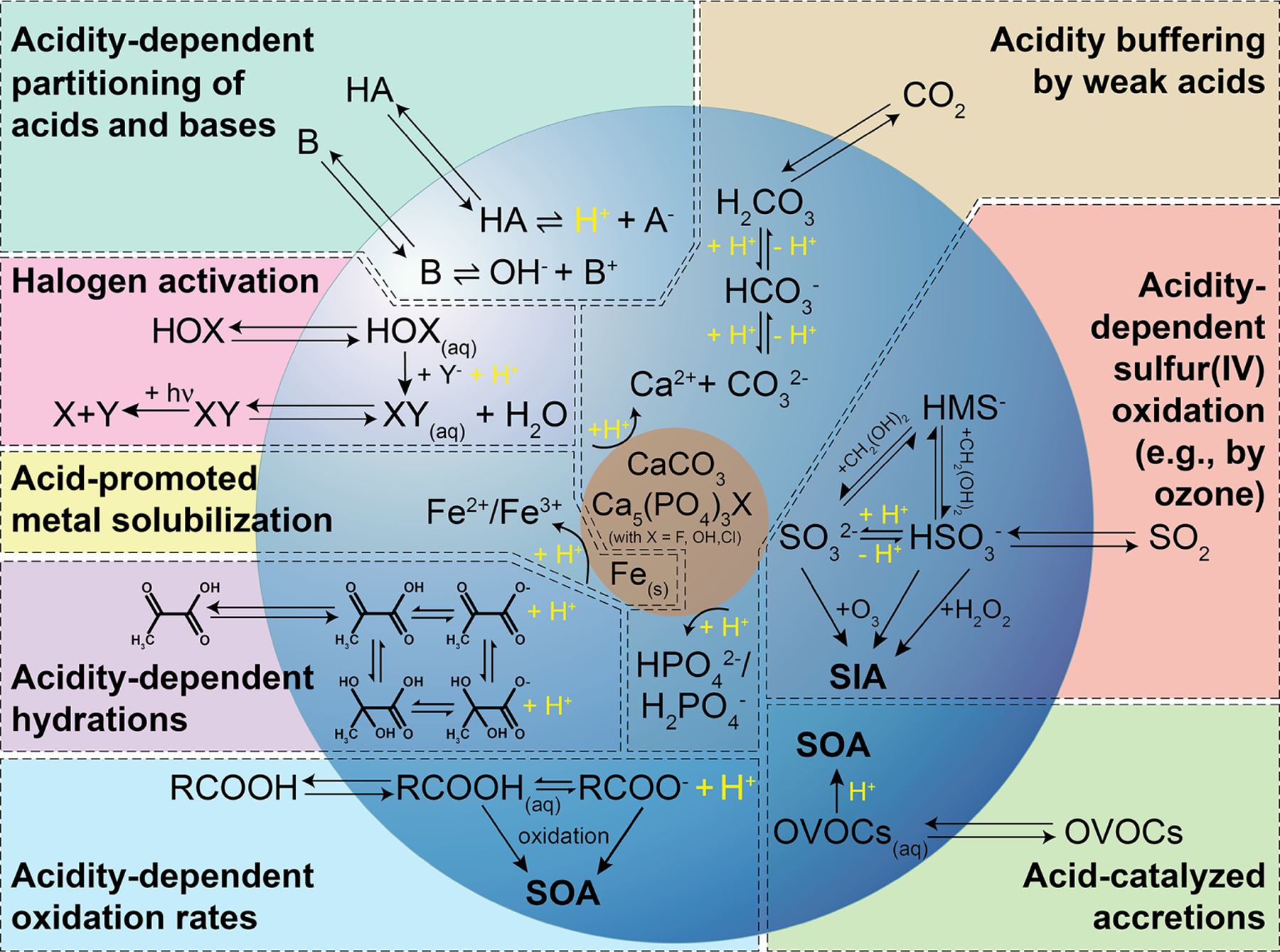
Schematic of chemical processes influenced by and affecting acidity in tropospheric aerosols.

The acidity of the atmospheric aqueous phase (i.e., deliquesced aerosol particles, cloud, and fog droplets) impacts human health, climate, and terrestrial and oceanic ecosystems (see, e.g., the companion article of Pye et al., 2020 and references therein). Changes in acidity in these aqueous media can arise due to the uptake of acidic or basic gases, coalescence, or chemical reactions in the aqueous phase. In turn, the acidity of aerosols influences the phase partitioning of semi-volatile species, particulate matter (e.g., Nenes et al., 2020b), their deposition rates (e.g., Nenes et al., 2021), and the rates and types of their chemical transformations. As a result of this two-way coupling between acidity and chemistry, acidity in atmospheric aqueous aerosol matrices is controlled not only by thermodynamic equilibrium but also by mass transfer, chemical reaction kinetics, and emissions. Multiphase oxidation and reduction processes in atmospheric waters are strongly linked to the acidity-dependent uptake of acidic or basic compounds, which, in turn, affects the phase partitioning and the composition of aerosol particles. Moreover, the acidity level directly impacts chemical transformations, but the acidity itself is also influenced as a consequence of such processes. Figure 1 illustrates important tropospheric chemical processes in aqueous atmospheric matrices that are influenced by acidity and affecting acidity.

The most important source of acidity in aqueous aerosols in the troposphere is the uptake and in situ formation of strong acids, including sulfuric acid, a classic and important compound connected to anthropogenic pollution. Acid formation in aqueous atmospheric phases is itself influenced by acidity, but, more importantly, it also substantially increases the acidity of those media. Important acidityinfluenced chemical processes, such as the conversion of sulfur(IV) to sulfur(VI) (Calvert et al., 1985; Faloona, 2009; Harris et al., 2013; Turnock et al., 2019), and acid-driven and acid-catalyzed reactions of organic compounds (McNeill et al., 2012; Herrmann et al., 2015), contribute significantly to both secondary inorganic aerosol (SIA) and secondary organic aerosol (SOA) formation. These constituents are often responsible for a large fraction of fine particulate matter (Jimenez et al., 2009). Due to their relative abundance and importance, they are strongly associated with aerosol effects on climate (Charlson et al., 1992; Boucher et al., 2013; Seinfeld et al., 2016; McNeill, 2017), air quality (Fuzzi et al., 2015), visibility (Hyslop, 2009), ecosystems (Keene and Galloway, 1984; Adriano and Johnson, 1989; Baker et al., 2021), and human health (Pöschl, 2005a; Pope and Dockery, 2012; Lelieveld et al., 2015). Therefore, changes in acidity can significantly affect the global impacts of aerosols (Turnock et al., 2019).

Acidity-dependent chemical reactions also modify the tropospheric multiphase oxidant budget. For instance, the activation of halogen radicals is promoted by acidity (see Fig. 1) and can substantially affect the tropospheric oxidative capacity (Vogt et al., 1996; von Glasow et al., 2002a; Pechtl and von Glasow, 2007; Sherwen et al., 2016, 2017; Hoffmann et al., 2019b). Acidity can indirectly affect aerosol and cloud composition by promoting the solubilization of transition metals and other bioavailable nutrients such as phosphorus (Meskhidze et al., 2005; Nenes et al., 2011; Shi et al., 2011; Stockdale et al., 2016). Soluble transition metal ions (TMIs) can initiate enhanced HO_*x*_ chemistry in aqueous aerosol particles and clouds or catalyze S(IV) oxidation. Moreover, these solubilized metals, phosphorus, and semi-volatile inorganic reactive nitrogen molecules (NH_3_ and HNO_3_) can deposit to the ocean surface, contribute to the bioavailable nutrient budget, and, thus, impact biological activity and the carbon cycle. TMI solubilization also influences the impacts of atmospheric aerosols on human health (Fang et al., 2017). On the other hand, the acidity of aqueous solutions can be buffered (see Fig. 1; Weber et al., 2016; Song et al., 2019a) by chemical interactions of (i) marine and crustal primary aerosol constituents (e.g., carbonates, phosphates, and halogens), (ii) dissolved weak organic acids (e.g., formic acid, acetic acid, etc.), (iii) dissolved weak inorganic acids (e.g., HNO_3_, HCl, and HONO), and bases (e.g., ammonia and amines).

In comparison to other aqueous environments, such as sea water and continental surface waters, which are characterized by rather small acidity variations, atmospheric aqueous environments show much higher diversity (see Pye et al., 2020, for details). This is in part because of the huge concentration range of dissolved species in atmospheric waters, but it is also due to the decoupled exchange of acidic and basic species between the gas and condensed phases. Due to the technical challenges of sampling and/or characterizing the pH of aerosols, fogs, and cloud water, there is also comparatively limited data on the acidity of these phases in time and space. Pye et al. (2020) provide a more complete overview of the literature data on the acidity of atmospheric waters, which we briefly summarize here. Typical pH values for cloud and fog droplets lie between 2–7, while pH values for continental and marine aerosol particles have a larger range of −1–5 and 0–8, respectively (Herrmann et al., 2015; Pye et al., 2020, and references therein). Because of the importance of aerosol and cloud acidity for atmospheric processes and the environment, acidity has been a key subject of research for 3 decades. The majority of those studies were focused on clouds, motivated by acid rain and SIA formation. A detailed review on observations, thermodynamic processes, and implications of atmospheric acidity is given in Pye et al. (2020).

Here, we review in detail the impact of acidity on the chemical transformations of atmospheric aerosols, clouds, and fog water, with a focus on aqueous-phase chemical reaction kinetics and mechanisms. We also highlight how chemical reactions control acidity in atmospheric aqueous media. We first discuss the uptake of acidic and basic gases, as well as buffering phenomena, and then describe feedbacks between particle and droplet acidity, aqueous-phase inorganic (SO_2_ oxidation and halogen) chemistry, and organic chemistry. Finally, a summary addresses atmospheric implications and the need for future investigations, for example, in the context of reduced fossil fuel combustion emissions of key acid precursors in a changing world.

## 2 Fundamental physical and chemical processes of importance for acidity

### 2.1 Aqueous-phase partitioning of acidic and basic gases

The partitioning of acidic or basic gases to atmospheric aerosols or cloud and fog droplets can have a major influence on condensed-phase acidity. Similarly, the acidity of the aqueous phase itself influences the partitioning of dissociating species from the gas phase. Condensed-phase acidity also governs the back transfer or evaporation of dissociating compounds into the gas phase – an important acidity buffering process (see Sect. 2.2).

#### 2.1.1 The phase partitioning of acids and bases

The partitioning of a compound, between the gas phase, aqueous phase, and its ionic forms, is usually achieved in < 1 h for fine-mode aqueous aerosols and small cloud droplets (Dassios and Pandis, 1998; Ervens et al., 2003; Ip et al., 2009; Koop et al., 2011). Therefore, equilibrium conditions are often assumed in order to estimate the aqueous-phase concentrations. Exceptions include large droplets with higher pH values, droplets or particles with surface coatings, viscous aerosol particles, or highly reactive dissolving compounds, where mass transfer limitations in the gas or aqueous phase can prevent the attainment of equilibrium partitioning on relevant timescales. The assumption of a thermodynamic equilibrium in such a case may result in model biases (Ervens et al., 2003).

Assuming an ideal aqueous solution at equilibrium, i.e., neglecting, for example, mass transport limitations, chemical production and degradation processes, and non-ideal solution effects (i.e., considering the activity of ions in solution equal to their aqueous concentration), the aqueous-phase concentration of a soluble compound ([*A*]_aq_) is proportional to the partial pressure of the compound in the gas phase (*p*_*A*(air)_) and its Henry’s law constant *H*_*A*_. The Henry’s law constant (in moles per liter per atmosphere; hereafter mol L^−1^ atm^−1^) is defined as follows:

(1) $$H_{A}=\frac{{\left[A\right]}_{\text{aq}}}{p_{A(\text{air})}}.$$

Once an acid is taken up into an aqueous solution, it can dissociate into a hydrogen ion (H^+^) and anions (A^*z*−^), the degree of which depends on its tendency for dissociation, characterized by an equilibrium dissociation constant *K*_a_, and the acidity of the aqueous environment. Consequently, an effective Henry’s law constant, $H_{A}^{*}$, e.g., for a diacid, is defined by Eq. (2a). For a monoprotic acid, the third term in the parenthesis is omitted (*K*_a2_ = 0). For typical atmospheric monoprotic bases, such as NH_3_ or dimethylamine, the corresponding effective Henry’s law constant, $H_{A}^{*}$, is defined by Eq. (2b). In Eq. (2b), *K*_a_ is the equilibrium dissociation constant *K*_a_ of the base cation.

(2a) $$H_{A(\text{acid})}^{*}=H_{A}\left(1+\frac{K_{\text{a}1}}{\left[{\text{H}}^{+}\right]}+\frac{K_{\text{a}1}K_{\text{a}2}}{{\left[{\text{H}}^{+}\right]}^{2}}\right)$$

(2b) $$H_{A(\text{base})}^{*}=H_{A}\left(1+\frac{\left[{\text{H}}^{+}\right]}{K_{\text{a}}}\right).$$

Together with the liquid water content (LWC), the acidity of an aqueous solution can substantially affect the partitioning of dissociating compounds to the aqueous aerosol or cloud phase. Increasing acidity leads to a decrease in the effective partitioning of acids, an increase in the effective partitioning of bases, and vice versa. For example, the partitioning of nitrate to the particle phase varies dramatically across the typical range of aerosol pH, with nearly 100 % of nitrate existing as HNO_3_ in the gas phase at pH 1 and near-complete particle-phase partitioning at pH 4. As a result, even small biases in predicted particle pH in air quality models can result in over- or under-predictions of fine particle mass (Vasilakos et al., 2018). Since atmospheric waters are typically acidic, bases are predominantly present in their protonated form, and their partitioning is not greatly altered by typical variations in pH. Hence, this section mainly focuses on the impact of acidity on the partitioning of weak acids into aqueous aerosols, cloud, and fog droplets.

From Eq. (1) and the ideal gas law, the concentration of the dissociating compound in the gas $\left(C_{A_{\text{air}}}\right)$ and aqueous $\left(C_{A_{\text{aq}}}\right)$ phase, with respect to the volume of air, can be determined. Moreover, the aqueous-phase fraction of *A* $\left(X_{A_{\text{aq}}}\right)$, i.e., the ratio of the aqueous-phase concentration of compound *A* and the overall multiphase concentration (sum of *A* in the gas and aqueous phase, including undissociated and dissociated forms of *A*) can accordingly be calculated by Eq. (3) (see Seinfeld and Pandis, 2006, for details).

(3) $$X_{A_{\text{aq}}}=\frac{C_{A_{\text{aq}}}}{\left(C_{A_{\text{aq}}}+C_{A_{\text{air}}}\right)}\;=\frac{H_{A}^{*}\cdot R^{*}\cdot T\cdot \text{LWC}\cdot 10^{-6}}{1+H_{A}^{*}\cdot R^{*}\cdot T\cdot \text{LWC}\cdot 10^{-6}}.$$

Here, $C_{A_{\text{air}}}$ is the concentration of *A* in air (moles per liter of air; hereafter $\text{mol }{\text{L}}_{\text{air}}^{-1}$), $C_{A_{\text{aq}}}$ is the aqueous-phase concentration of *A* in the volume of air $\left(\text{mol }{\text{L}}_{\text{air}}^{-1}\right)$, and R* is the universal gas constant (0.082058 atm L_air_ mol^−1^ K^−1^). *T* (Kelvin) is the temperature, $H_{A}^{*}$ is the effective Henry’s law constant (moles per liter of water per atmosphere; hereafter $\text{mol }{\text{L}}_{\text{water}}^{-1}\;{\text{atm}}^{-1}$), and LWC is the liquid water content (grams of water per cubic meter of air; hereafter $\text{g }{\text{m}}_{\text{air}}^{-3}$). Considering activities instead of concentrations, Eq. (3) modifies to Eqs. (3a) and (3b) for monoprotic acids and bases as follows (see Nenes et al., 2020, and Guo et al., 2017 for details):

(3a) $$X_{\text{aq},\text{acid}}=\frac{H_{A}\cdot K_{\text{a}1}\cdot R^{*}\cdot T\cdot \text{LWC}\cdot 10^{-6}}{\gamma_{{\text{H}}^{+}}\cdot \gamma_{{\text{A}}^{-}}\cdot \left[{\text{H}}^{+}\right]+H_{A}\cdot K_{\text{a}1}\cdot R^{*}\cdot T\cdot \text{LWC}\cdot 10^{-6}}$$

(3b) $$X_{\text{aq},\text{base}}=\frac{H_{A}\cdot K_{\text{a}1}\cdot R^{*}\cdot T\cdot \text{LWC}\cdot 10^{-6}}{1+\frac{\gamma_{H^{+}}}{\gamma_{{\text{B}}^{+}}}\cdot \frac{H_{A}\cdot \left[{\text{H}}^{+}\right]}{K_{\text{a}1}}\cdot R^{*}\cdot T\cdot \text{LWC}\cdot 10^{-6}},$$

where $\gamma_{{\text{H}}^{+}}$, $\gamma_{{\text{A}}^{-}}$, and $\gamma_{{\text{B}}^{+}}$ are the single-ion activity coefficients for H^+^, the acid anion (A^−^), and the base cation (B^+^), respectively, which can be calculated for a known ion composition using thermodynamic models (e.g., ISORROPIA-II in Fountoukis and Nenes, 2007; E-AIM in Clegg and Seinfeld, 2006; AIOMFAC in Zuend et al., 2008).

**Figure 2.**
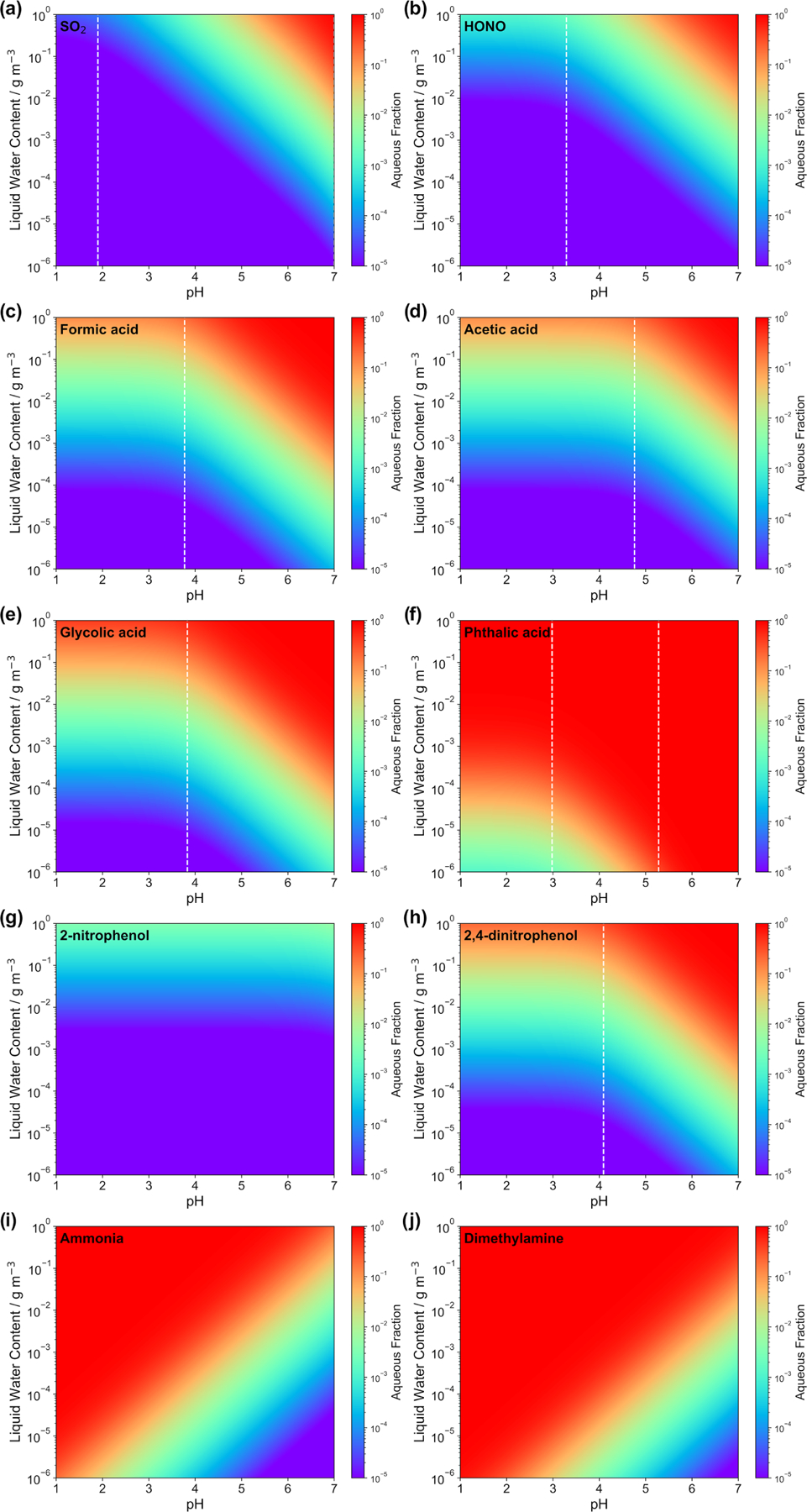
Calculated aqueous-phase fraction $X_{A_{\text{aq}}}$ of eight selected weak acids (i.e., **a** SO_2_, **b** HONO, **c** formic acid, **d** acetic acid, **e** glycolic acid, **f** phthalic acid, **g** 2-nitrophenol, and **h** 2,4-dinitrophenol) and bases (i.e., **i**: ammonia and **j**: dimethylamine) as a function of the LWC and acidity. The black lines are the isolines of the aqueous fractions of 10^−*i*^ (*i* = 1, …, 6). The dashed white lines indicate *pK*_a_ values of the corresponding acids (except for the two bases and for 2-nitrophenol due to the very high *pK*_a_ of 7.2; see Table S1).

Figure 2 displays the aqueous fraction, $X_{A_{\text{aq}}}$, of eight weak atmospheric acids (sulfurous acid, nitrous acid, formic acid, acetic acid, glycolic acid, lactic acid, benzoic acid, phthalic acid, 2-nitrophenol, and 2,4-dinitrophenol) and two important atmospheric bases (ammonia and dimethylamine) as a function of the LWC and acidity, as calculated by Eq. (3). For the plots, an acidity range ([H^+^] = 10^−1^–10^−7^ mol L^−1^) and a liquid water content range (10^−6^–1 g m^−3^) have been considered that represent typical values for tropospheric aqueous aerosols, cloud and fog droplets, and haze (see Herrmann et al., 2015). A temperature of 298 K was assumed. It should be noted that temperature plays an important role for the effective solubility of trace gases. In general, as temperature decreases, the trace gas effective solubility increases. Thus, clouds at the top of the mixing layer height (~285 K typically) have higher aqueous fractions than aerosol water near the surface on a hot summer day. Similarly, winter hazes should also have higher aqueous fractions than summertime haze events. Therefore, the aqueous fractions shown in Fig. 2 should be used carefully. Note, the *H*_*A*_ and *pK*_a_ values applied for the idealized calculation of LWC and acidity-dependent aqueous fraction $X_{A_{\text{aq}}}$ are listed in Table S1 in the Supplement.

Examples in Fig. 2 illustrate that acidity, along with the LWC, strongly influences the phase partitioning of weak acids and bases into the aqueous phase. The partitioning into the aqueous phase is more effective for pH values well above the individual *pK*_a_ values of each acidic compound. Below the individual *pK*_a,1_ value, only the Henry’s law constant and the LWC limit the uptake. High LWCs (0.1–1 g m^−3^) typically associated with cloud conditions and, accordingly, less acidic media (pH > 4) favor phase partitioning towards the aqueous phase for most of the weak acids, as well as for ammonia. Less water-soluble acids (i.e., with lower *H* values), such as dissolved SO_2_ and HONO, display fractions above 0.1 only under less acidic conditions for typical cloud LWC values. Thus, even at colder cloud temperatures than the 298 K used in Fig. 2, where $H_{A}^{*}$ is larger, SO_2_ and HONO largely remain in the gas phase under typical cloud acidity conditions. Hence, note that $X_{A_{\text{aq}}}$ values of SO_2_ are typically in the range of 0.005 to 0.5, depending on both the cloud acidity and temperature. Under typical aerosol conditions (0 ≤ pH ≤ 4; see, e.g., Pye et al., 2020; 10^−6^ ≤ ALWC ≤ 10^−4^ g m^−3^; see, e.g., Herrmann et al., 2015), the LWC restricts uptake and only very small fractions of the less water-soluble and weak acids can partition in the aqueous particle phase due to their *pK*_a_ values (typically above 4). Moreover, very weak acids, with *pK*_a_ values larger than 7 (e.g., 2-nitrophenol) show almost no acidity dependency in the plotted acidic range. On the other hand, for stronger acids, the LWC and acidity impact is even lower due to their lower and/or multiple *pK*_a_ values. For example, phthalic acid partitions in substantial amounts into the aqueous phase for a large range of acidity and LWC conditions. The implication is that only very water-soluble and strong acids are expected to remain in acidic aerosol solutions. However, it is worth mentioning again that this treatment neglects several other factors and processes affecting the partitioning of acids in the aqueous phase, particularly under concentrated aqueous aerosol conditions. Specifically, volatile acids (e.g., formic and acetic) often show substantial deviations from this theory (see Nah et al., 2018), for instance, because of the formation of organic salts which can increase their particle partitioning by 2 orders of magnitude (Meng et al., 2007). In practice, weak acid anions are often measured in non-negligible fractions in the particle phase (Tanner and Law, 2003; Limbeck et al., 2005; van Pinxteren and Herrmann, 2007; Bao et al., 2012; Nah et al., 2018; Teich et al., 2019).

#### 2.1.2 Non-ideal solutions

At less than 100 % relative humidity (non-cloud conditions), aqueous aerosol solutions exist as a highly concentrated, complex mixture of electrolytes. Interionic and ion-molecule interactions are critically important under those conditions, leading to thermodynamically non-ideal behavior (Pitzer, 1991; Zaveri, 2005; Cappa et al., 2008; Zuend et al., 2008; Herrmann et al., 2015; Rusumdar et al., 2016, 2020). Therefore, parameters that have been developed for dilute aqueous solutions do not strictly apply to aerosol-phase chemistry.

Nevertheless, several such principles, such as Henry’s law, have been shown experimentally to hold for the aqueous aerosol phase (Kroll et al., 2005; Sumner et al., 2014), although it may be necessary to account for phenomena such as salting effects (Kampf et al., 2013; Waxman et al., 2015). Factors such as ionic strength, the different chemical composition of the concentrated solution, other favored chemical pathways, shifted chemical equilibria (e.g., salting in and salting out, hydration, metal complexes, dimer and polymer, etc.), and more can significantly affect overall phase partitioning and reaction rates. The inclusion of these factors into the calculation of the effective Henry’s law constant can explain increased or decreased aqueous-phase partitioning of chemical compounds, such as atmospheric carbonyl compounds (Kampf et al., 2013; Waxman et al., 2015) and organic monocarboxylic acids (Limbeck et al., 2005; Meng et al., 2007), compared to what may be expected based on aqueous solubility alone. Ionic strength effects are also believed to be critically important for acidity producing in-particle chemical reactions, such as S(IV) oxidation (Martin and Hill, 1987b; Lagrange et al., 1993, 1994; Maaß et al., 1999; Ali et al., 2014; Cheng et al., 2016), although experimental data at the extremely high ionic strengths typical of atmospheric aerosols are limited. The first models, treating both non-ideal solution effects and their feedbacks on occurring chemical processes in detail, have been developed in the last few years and have enabled advanced investigations, e.g., on the phase partitioning issues (Rusumdar et al., 2016).

### 2.2 Acidity buffering

**Figure 3.**
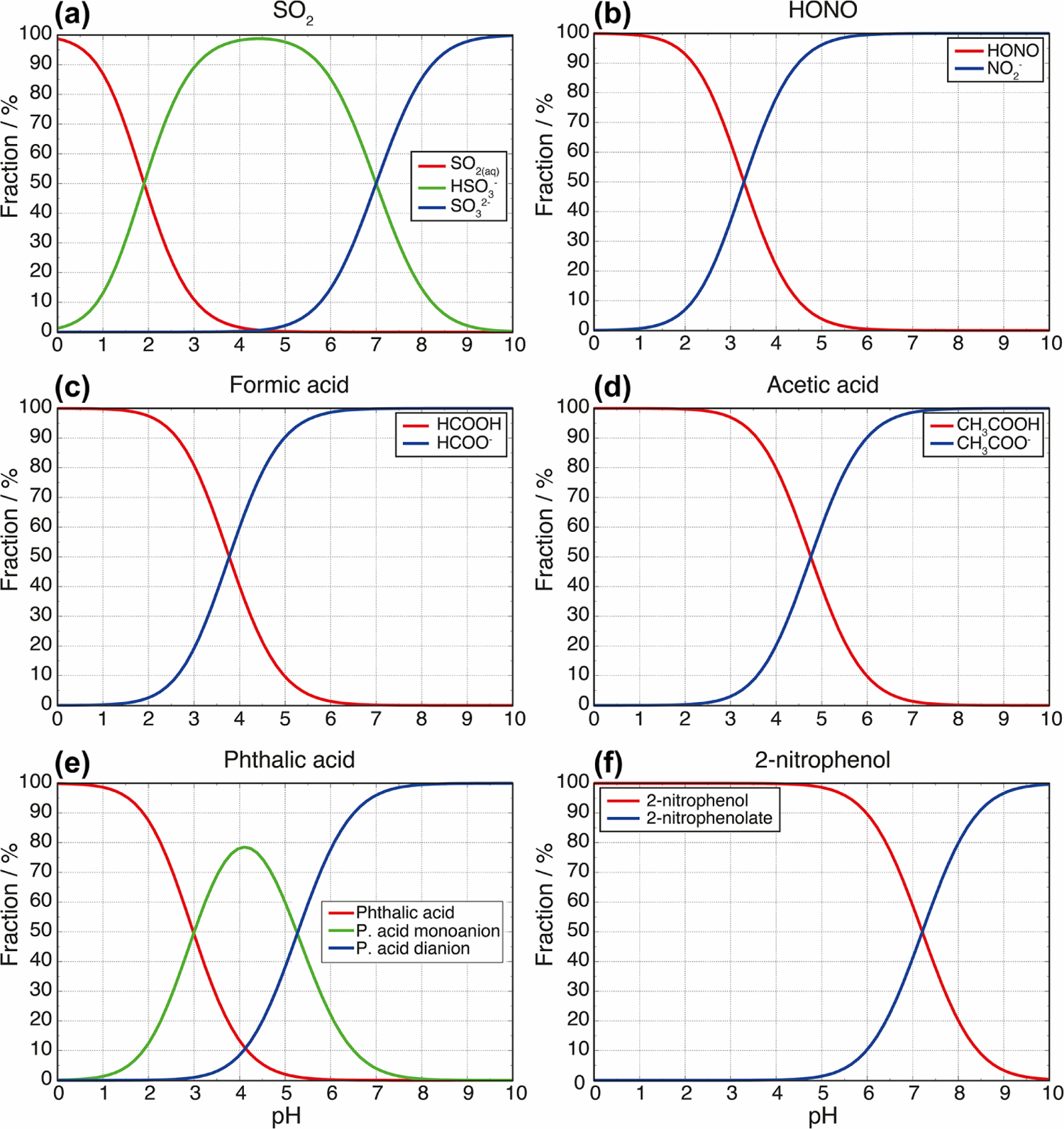
Ion speciation of dissolved (**a**) SO_2_, (**b**) HONO, (**c**) formic acid, (**d**) acetic acid, (**e**) phthalic acid, and (**f**) 2-nitrophenol as a function of pH.

The response of pH in the atmospheric aqueous phases to a perturbation in acidity can be strongly affected by the presence and ability of weak acids or bases to buffer against that change. A buffer is a mixture of a weak acid and its conjugate base (e.g., formic acid and formate) or a mix of a weak base and its conjugate acid (e.g., ammonia and ammonium). The buffering effect, a resistance to pH change, comes from changes in the equilibrium between concentrations, for example, of a weak acid and a conjugate base. The Henderson–Hasselbach equation (Eq. 4) is as follows:

(4) $$\text{pH}=pK_{\text{a}}+\text{log}\left(\frac{\left[{\text{A}}^{-}\right]}{\left[\text{HA}\right]}\right),$$

and is used to calculate the pH of a buffer solution based on the acid dissociation constant (*K*_a_) and the concentrations of the acid [HA] and its conjugate base [A^−^]. Ion speciation curves for a wide range of atmospherically relevant weak acids are shown in Fig. 3. The magnitude of the buffering effect is greatest when the solution pH is equal to the *pK*_a_ of the weak acid buffer (intersection points of the speciation curves as shown in Fig. 3). Consider, for example, the case of formic acid (*pK*_a_ = 3.8 at 298 K) and formate (see Fig. 3c). If protons are added (e.g., through the addition of a strong acid such as sulfuric acid) to a solution containing formate or formic acid, and the solution pH is far above or below the 3.8 *pK*_a_ of formic acid, each added proton will directly increase the H^+^ concentration in the solution. When the solution pH, however, is close to the formic acid *pK*_a_ (where the concentrations of formic acid and formate are equal), many of the added protons will be consumed in converting formate to formic acid, thereby slowing the pH decline of the solution. For diprotic acids, buffering occurs at each of the two acid dissociation steps. Carbonate buffering is a relevant example for atmospheric cloud and fog droplets. The *pK*_a_ values for carbonic acid and bicarbonate are 6.4 and 10.3 at 298 K. A titration by acid addition beginning at pH 12, therefore, would show strong buffering at pH 10.3 and again at pH 6.4, with the latter being much more relevant for atmospheric water. Moreover, in mineral dust and volcanic particles that can bear phosphate minerals such as apatite, dissolved phosphate can act as a buffer. But, unlike carbonates, the phosphate buffer cannot be lost owing to volatilization. Nevertheless, the buffering by phosphate in other kind of atmospheric aerosol particles is negligible because of the typically extremely low phosphate concentration.

The buffering capacity (*β*), a measure to quantitatively express the resistance of an aqueous solution towards acidity changes, is defined for a monoprotic acid by Eq. (5) (see Urbansky and Schock, 2000, for details). The buffering capacity *β* expresses the amount of an acid or base concentration addition (d[*C*_a*/*b_]) needed to cause a certain change in pH (d(pH)).

(5) $$\beta =\frac{\text{d}\left[C_{\text{a}/\text{b}}\right]}{\text{d}(\text{pH})}\;=\text{ln }10\cdot \left(\frac{K_{\text{W}}}{\left[{\text{H}}^{+}\right]}+\left[{\text{H}}^{+}\right]+\underset{i}{\sum }\frac{{\left[C\right]}_{i}\cdot K_{\text{a},i}\cdot \left[{\text{H}}^{+}\right]}{{\left(K_{\text{a},i}+\left[{\text{H}}^{+}\right]\right)}^{2}}\right).$$

**Figure 4.**
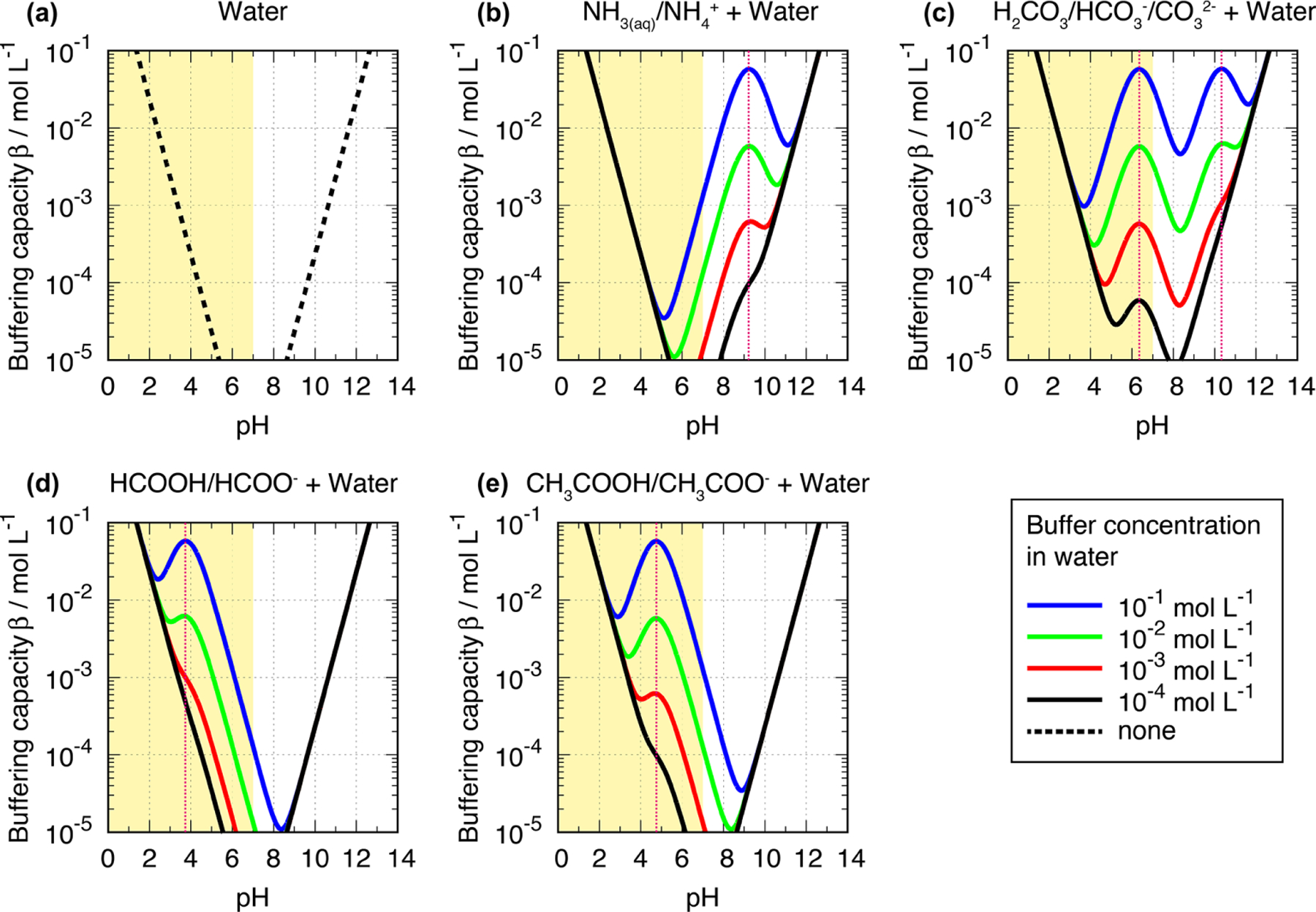
Buffering capacity *β* of (**a**) water, (**b**) ammonia or ammonium, and (**c**) carbonate, bicarbonate, or carbonic acid (top), as well as (**d**) formic and (**e**) acetic acid (bottom), as a function of pH. The atmospherically relevant range of cloud and aerosol pH is marked in yellow, and the *pK*_a_ values of the corresponding buffers are marked with dotted pink lines.

Equation (5) and the plotted examples in Fig. 4 reveal that very high and very low acidity conditions show significantly increased buffering capacities. The first term $\left(\text{ln }10\cdot \frac{K_{\text{W}}}{\left[{\text{H}}^{+}\right]}\right)$ and second term (ln 10·[H^+^]) of Eq. 5 represent the terms for water (H^+^ and OH^−^, respectively) and create the lower buffering capacity limits (dashed lines in Fig. 4a) with a minimum at pH 7 (not shown in Fig. 4a). The first and the second terms of Eq. (5) lead to high *β* values at high and low pH conditions, respectively. The third term adds an additional buffering capacity of all other buffers in the aqueous solution. So, added buffers in the solution can introduce local maxima of *β* between very acidic and very alkaline conditions, where the contribution of the first and the second terms to the *β* is small. In the case of one monoprotic acid present in an aqueous solution, the maximum of the buffering capacity occurs at the *pK*_a_ value of the acid, as mentioned above. Furthermore, Eq. 5 shows that buffering capacity, i.e., the amplitude of the local maxima, depends on the concentration of the buffer compound. This agrees with findings in the field, e.g., in fog samples analyzed by Collett et al. (1999) (see discussion below). Furthermore, this dependency implies a rather high buffering capacity in regions with high multiphase concentrations of weak inorganic and organic acids and bases or high amounts of particulate buffers such as carbonate components. However, the latter are most important in buffering the acidity of supermicron particles or fog and cloud droplets that activate on them.

Titrations of actual cloud and fog samples have exhibited buffering across a wide pH range, suggesting the importance of pH buffering by a variety of compounds with different *pK*_a_ values. For example, Collett et al. (1999) report titrations of fog samples collected at urban and rural locations in California’s San Joaquin Valley. Observed buffering in rural fogs in the study could be nearly accounted for based on ammonia and bicarbonate concentrations present in the fog samples. By contrast, significant additional buffering (*β* up to 10^−4^ mol L^−1^) was observed in urban fogs over a broad pH range from 4 to 7. The amount of additional buffering was strongly correlated with concentrations of organic compounds in fogs from these environments, with relevant organic buffering agents likely including carboxylic and dicarboxylic acids and phenols.

The buffering phenomenon described above is often referred to as “internal buffering”, since it derives from shifts in equilibrium concentrations of compounds present in solution. The exchange of material with the gas phase can also lead to “external buffering”. Perhaps the most important form of external buffering is the uptake of additional ammonia from the gas phase in response to a drop in solution pH, as outlined by Liljestrand (1985) and Jacob et al. (1986). Corresponding buffering in atmospheric aerosols from semi-volatile partitioning also occurs, as shown by Meng et al. (2007), Weber et al. (2016), and Song et al. (2019a), as well as recently by Zheng et al. (2020). However, it should be noted that the effect of aerosol pH buffering from semi-volatile gases on relevant chemical processes has not been studied comprehensively and still represents an issue for future research.

One important consequence of pH buffering in fog and cloud drops is an effect on rates of pH-sensitive aqueous reactions. The presence of (internal and/or external) acid buffering in cloud and fog droplets can slow droplet acidification and maintain greater rates of reaction for strongly pH-dependent aqueous chemical pathways (e.g., the oxidation of S(IV) by ozone) which are favored by high pH.

## 3 Sources of acidity and alkalinity

Acidic and alkaline components of tropospheric aerosols result from primary gas and aerosol particle emissions, as well as secondary gas-phase and aqueous-phase formation processes (Pöschl, 2005b; Seinfeld and Pandis, 2006; Seinfeld, 2015; Zhang et al., 2015). The most important acidic chemical components of aerosols and cloud and fog droplets are sulfuric acid, nitric acid, nitrous acid, and hydrochloric acid, as well as organic mono- and dicarboxylic acids (e.g., formic acid, acetic acid, oxalic acid, etc.; Vet et al., 2014; Zhang et al., 2015). Then, the most important basic components of aerosols, cloud, and fog droplets are ammonium, amines and alkali/alkaline Earth metals (U.S. EPA, 2000; Vet et al., 2014; Zhang et al., 2015). The global contribution of different acid and base ions to precipitation has been assessed by Vet et al. (2014). As precipitation samples provide both compositional and acidity information for some portion of the vertical column, these data represent a useful means for pointing out the spatial sources and sinks of gas and aqueous-phase acidity and alkalinity components.

**Figure 5.**
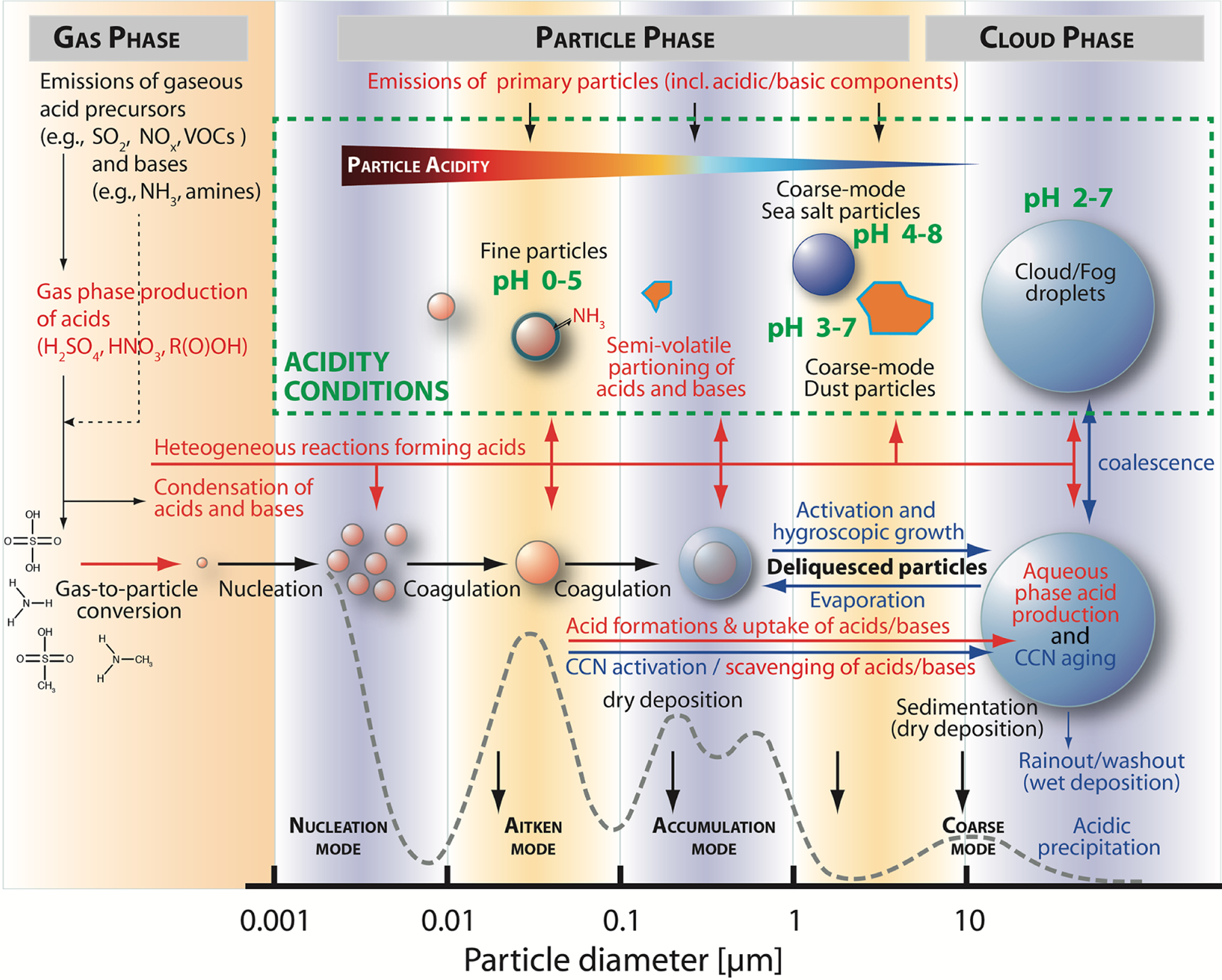
Schematic of sources (red text) and conditions of acidity in different aqueous aerosol particles (green text) together with microphysical and chemical processes that are able to influence the acidity of tropospheric aerosols (created after Raes et al., 2000, and McMurry, 2015). The blue text describes microphysical processes of CCNs and cloud and fog droplets. The dashed gray line represents an aerosol number size distribution, based on McMurry (2015).

Gaseous acids can be directly emitted into the troposphere from primary sources such as biomass combustion, traffic (fuel combustion), domestic heating, industrial burning, agriculture, soil, and vegetation (Chebbi and Carlier, 1996; Paulot et al., 2011; Spataro and Ianniello, 2014; Kawamura and Bikkina, 2016). Moreover, gaseous acids can be formed secondarily by gas-phase oxidations of emitted acid precursor compounds such as SO_2_, NO_*x*_, and VOCs (Chebbi and Carlier, 1996; Paulot et al., 2011; Spataro and Ianniello, 2014; von Schneidemesser et al., 2015; Zhang et al., 2015; Kawamura and Bikkina, 2016). The gas-phase OH oxidation of SO_2_ is an important source of gaseous sulfuric acid and, after condensation, of particulate sulfate (von Schneidemesser et al., 2015; Zhang et al., 2015). SO_2_ is emitted from anthropogenic activities, such as the combustion of sulfur-containing fuels, and various natural sources, such as volcanos (Smith et al., 2001, 2011; Seinfeld, 2015). Moreover, it is formed from the oxidation of natural precursors such as dimethyl sulfide (DMS; CH_3_SCH_3_) emitted by oceanic phytoplankton (Seinfeld and Pandis, 2006). The gaseous oxidation pathway of SO_2_ contributes also to newly formed particles (nucleation; Zhang et al., 2015) which are expected to be quite acidic. Furthermore, the gaseous oxidation of NO_*x*_ and VOCs can lead to the formation of nitric and nitrous acid and organic acids (e.g., formic, acetic, and oxalic acid; Chebbi and Carlier, 1996; Paulot et al., 2011; Spataro and Ianniello, 2014; Zhang et al., 2015; Kawamura and Bikkina, 2016). By contrast, gaseous bases such as ammonia and amines are almost exclusively emitted into the troposphere, mainly from agriculture due to intensive stock farming and the use of NH_3_-based fertilizer applications. Moreover, bases are released from biomass burning, vehicles, industrial processes, and as a consequence of volatilization from soils and oceans (U.S. EPA, 2000; Behera et al., 2013). As shown in Fig. 5, subsequent to their emission or secondary formation, gaseous acids and bases can condense on existing aerosol particles or fog and cloud droplets and can then contribute to aerosol acidity.

Acidic and alkaline aerosol components are also (i), primarily, emitted by anthropogenic and natural processes (Zhang et al., 2015) or, secondarily, formed in aqueous aerosol solutions or at their interface (see Sects. 4 and 5 below). Important anthropogenic primary sources of acidic and alkaline aerosols (see Fig. 5) are urban combustion aerosols and agricultural aerosols, including, e.g., agricultural ammonia from livestock farming. Important natural primary sources of acidic and alkaline aerosols are sea spray, desert dust, biomass burning, and volcanic emissions. Besides the secondary acid formation in the gas phase, in-cloud oxidation of SO_2_ contributes more than 50 % globally to sulfate aerosol mass formation (Alexander et al., 2009; see Sect. 4.1 for details). Thus, the aqueous-phase formation of sulfate from the oxidation of SO_2_ is the largest source of acidity in the atmosphere. However, besides sulfate, other acidic components are also secondarily formed in aqueous aerosols such as nitrate, chloride, formate, acetate, and oxalate (see Chebbi and Carlier, 1996; Spataro and Ianniello, 2014; Ervens, 2015; Zhang et al., 2015; Kawamura and Bikkina, 2016).

In the past, emissions of SO_2_ in industrialized countries were the predominant cause of the strong acidification of aerosol particles, cloud droplets, and precipitation, typically known as the acid rain phenomenon (Adriano and Johnson, 1989; Seinfeld and Pandis, 2006). However, due to strongly reduced anthropogenic sulfur emissions in some parts of the world, a reduction in cloud and fog acidity has been observed over recent decades (see Pye et al., 2020). As a consequence of the changing acid and base sources, the composition of continental aerosol particles and cloud, fog, and/or rain droplets will most likely continue to evolve toward compositions observed preindustrially in rural continental areas, e.g., in North America and Western Europe. These environments are characterized by higher contribution of organic acids and chloride due to (i) lower rates of acid displacement (see, e.g., Pye et al., 2020, and references therein for further details on this topic) and (ii) lower abundances of sulfate and nitrate mass (see precipitation composition data compiled by Vet et al., 2014). In such a future environment, natural acidity sources become a much more important source for the acidity of tropospheric cloud, fog and/or rain droplets. On the other hand, no significant changes are expected for the acidity of marine droplets, except downwind of continents. Their main acidity and alkalinity sources, such as the emission of DMS, marine NH_3_, and sea salt particles containing chloride and base cations, are not expected to change significantly. However, it should be mentioned that the impact of climate change, including higher temperatures and ocean acidification and related changes in the ocean biochemistry, may unequally affect the emission of DMS in different regions. The effects of climate change on DMS emission patterns are still under debate due to the complex interactions of marine biochemistry and atmosphere–ocean interactions (Six et al., 2013; Gypens and Borges, 2014; Dani and Loreto, 2017; Hopkins et al., 2020).

## 4 Interactions of acidity and chemical processes: inorganic systems

In this section, the feedbacks between particle/droplet acidity and key inorganic chemical subsystems, the sulfur(IV) oxidation, and tropospheric halogen chemistry are discussed in detail.

### 4.1 Acidity and sulfur oxidation

In addition to its reaction with OH in the gas phase, SO_2_ is oxidized via heterogeneous and multiphase reactions in clouds, fog, or aerosol particles to form particulate sulfate. Sulfate is a major component of PM_2.5_, especially in areas affected by emissions from burning coal or other sulfur-containing fossil fuels (Attwood et al., 2014). Because the sulfate lifetime is of the order of days (Barth et al., 2000), sulfate contributes to regional haze and acid deposition, as well as local air pollution.

Once in the aqueous phase, SO_2_ is hydrated and undergoes acid–base equilibrium to form other S(IV) species, i.e., bisulfite $\left({\text{HSO}}_{3}^{-}\right)$ (*pK*_a,R1_ = 1.9) and sulfite $\left({\text{SO}}_{3}^{2-}\right)$ (*pK*_a,R2_ = 7.2). The hydration of SO_2_ upon uptake alone, according to Reaction (R1) already leads to the release of acidity, as follows:

(R1) $${\text{SO}}_{2}\cdot {\text{H}}_{2}\text{O}\overset{K_{\text{a1}}}{⇋}{\text{HSO}}_{3}^{-}+{\text{H}}^{+}$$

(R2) $${\text{HSO}}_{3}^{-}\overset{K_{\text{a}2}}{⇋}{\text{SO}}_{3}{}^{2-}+{\text{H}}^{+}.$$

**Figure 6.**
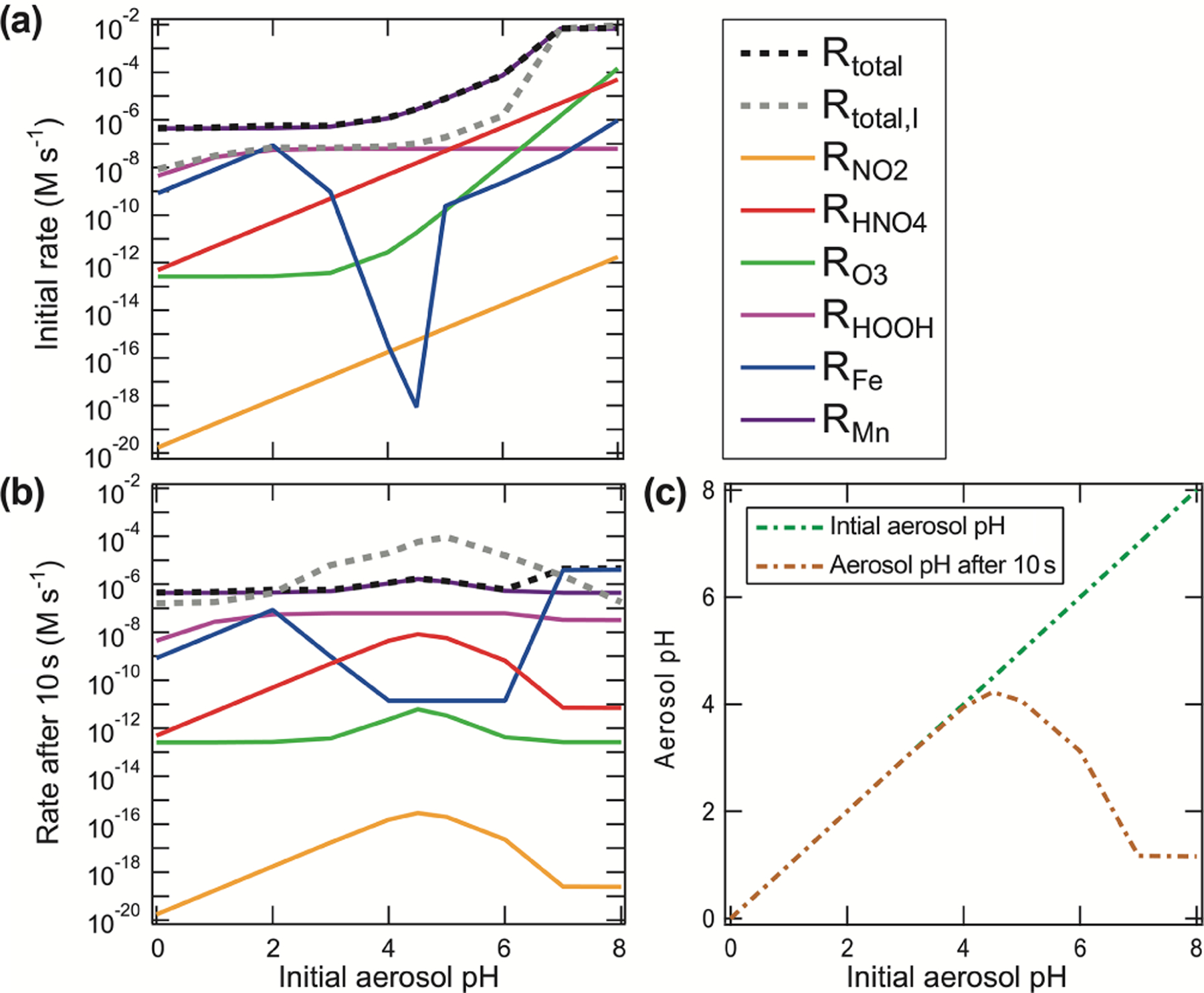
S(IV) oxidation rates for Beijing winter haze conditions (following Cheng et al., 2016). Shown are (**a**) initial S(IV) oxidation rates, (**b**) S(IV) oxidation rates after 10 s of reaction, and (**c**) aerosol pH after 0 and 10 s of reaction as a function of the initial aerosol pH. In the upper right legend, the S(IV) oxidation rates of the different oxidants (NO_2_, HNO_4_, O_3_, H_2_O_2_, Fe, and Mn) are shown in (**a**). The rates in (**b**) are listed together, with total S(IV) to S(VI) rates shown, both with and without taking the ionic strength at the maximum reported limit into account. Rates used were those recommended in this text.

S(IV) oxidation occurs in the aqueous phase to form S(VI) species (sulfate − ${\text{SO}}_{4}^{2-}$; bisulfate − ${\text{HSO}}_{4}^{-}$; sulfuric acid − H_2_SO_4_) leads to further acidification. S(IV) oxidation can take place via a number of chemical pathways, many of which are pH sensitive (Fig. 6). As a result of the equilibrium reactions described by Reactions (R1) and (R2), the effective solubility of SO_2_ in aqueous solutions increases rapidly with increasing pH (see Eq. 2a). Partly for this reason, and because of their relatively small liquid water content (~10^−9^ cm^3^ cm^−3^), sulfate formation in aerosols is generally believed to be less significant than in clouds and fog (Schwartz, 1986). Only S(VI) formation in the gas phase and in clouds is included in most large-scale atmospheric chemistry models. Globally, in-cloud formation is thought to be the dominant sulfate production pathway (~ 60 %), particularly over the oceans (generally > 75 %; Barth et al., 2000; Barrie et al., 2001; Manktelow et al., 2007; Alexander et al., 2009; Faloona, 2009; Alexander et al., 2012). However, there is evidence that significant sulfate formation also occurs in polluted urban areas during periods of high aerosol surface area and few clouds (Hering and Friedlander, 1982; Wang et al., 2014; P. He et al., 2018). This suggests that aerosol chemistry is also an important source of sulfate under some conditions.

In the aqueous phase, S(VI) species exist in acid–base equilibrium, according to the following:

(R3) $${\text{H}}_{2}{\text{SO}}_{4}\overset{K_{\text{a}3}}{⇋}{\text{HSO}}_{4}^{-}+{\text{H}}^{+}$$

(R4) $${\text{HSO}}_{4}{}^{-}\overset{K_{\text{a}4}}{⇋}{\text{SO}}_{4}{}^{2-}+{\text{H}}^{+}.$$

Since sulfuric acid is a very strong acid (*K*_a,R4_ ≅ 1000 mol L^−1^ at 298 K; Graedel and Weschler, 1981), almost no unionized H_2_SO_4_ exists in aqueous solution, and ${\text{HSO}}_{4}^{-}$ is significant only at pH < 3. As a consequence, the conversion of S(IV) to S(VI) in the aqueous phase increases the acidity of the cloud or aerosol particle not only by the initial acidification through the SO_2_ reaction with water but, additionally, through the dissociation of sulfuric acid. Some S(IV) oxidation reactions have other acidic byproducts, such as halous acid species HX (with X equal to Cl and Br) or HONO, and, thus, may contribute additional acidity to the aerosol (Fig. 6). Figure 6 illustrates that S(IV) oxidation under urban haze conditions can significantly contribute to the acidification of aerosols on a very short timescale. After a short period of chemical processing, aerosols are expected to reach pH 4.5 or lower. Particularly for haze particles with initial pH conditions above 4, a fast acidification can be modeled as a consequence of the higher initial S(IV) oxidation rates under less acidic conditions. Figure 6 shows that higher S(IV) to S(VI) oxidation rates under weakly acidic conditions (pH > 5) quickly generate sufficient H^+^ (after only 10 s), resulting in a significant decrease in the pH compared to the initial pH. Thus, in the absence of buffering, or a chemical OH^−^ source compensating for acidification, less acidic or even slightly basic particles are rapidly acidified in the troposphere. This is also known for freshly formed sea salt particles, which rapidly become acidified within minutes after their emission, characterized by a pH drop by about 4 pH units (Angle et al., 2021). Furthermore, Fig. 6 illustrates that processes that are initially important under low acidity conditions quickly become less important as the aerosol acidifies. For example, the importance of the O_3_ and HNO_4_ reaction drops significantly after 10 s, while the H_2_O_2_ oxidation is still at a similar level. To better understand this issue, in the next subsections, we outline the major S(IV) oxidation pathways, their sensitivity to the pH of the aqueous medium, and their potential to alter pH through the formation of acidic products.

### 4.2 S(IV) oxidation through O_3_, H_2_O_2_, ROOH, and HOX (with X equal to Cl, Br, and I)

Due to the pH-dependent partitioning of S(IV) species and, hence, solubility of SO_2_, most S(IV) oxidation mechanisms are highly pH dependent. However, S(IV) oxidation by H_2_O_2_ is only weakly pH dependent. At pH values typical of cloud water (pH = 2–7; Pye et al., 2020), S(IV) oxidation by H_2_O_2_ is thought to dominate sulfate production (Faloona, 2009) although other oxidants can be important at higher pH values or if H_2_O_2_ is depleted (e.g., Shen et al., 2012). In-cloud S(IV) oxidation by H_2_O_2_ proceeds via a reaction with ${\text{HSO}}_{3}^{-}$, followed by addition of H^+^ (see Reactions R5 and R6a).

(R5) $${\text{HSO}}_{3}{}^{-}+{\text{H}}_{2}{\text{O}}_{2}⇌{\text{SO}}_{2}{\text{OOH}}^{-}+{\text{H}}_{2}\text{O}.$$

(R6a) $${\text{SO}}_{2}{\text{OOH}}^{-}+{\text{H}}^{+}\to 2{\text{H}}^{+}+{\text{SO}}_{4}^{2-}$$

(R6b) $${\text{SO}}_{2}{\text{OOH}}^{-}+\text{HX}\to 2{\text{H}}^{+}+{\text{SO}}_{4}^{2-}+{\text{X}}^{-}.$$

Therefore, the intrinsic reaction rate decreases rapidly with increasing pH above pH 2 (McArdle and Hoffmann, 1983). This is balanced by the fact that the effective SO_2_ solubility increases with increasing pH. As a result, the overall rate is relatively independent of pH above pH ~ 1.5. The rate expression for S(VI) formation by S(IV) + H_2_O_2_ is given by McArdle and Hoffmann (1983), Lind et al. (1987), and Gunz and Hoffmann (1990) as follows:

(6a) $$R_{{\text{H}}_{2}{\text{O}}_{2}}=k_{6\text{a}}\frac{\left[{\text{H}}^{+}\right]\left[{\text{HSO}}_{3}^{-}\right]}{1+K_{5}\left[{\text{H}}^{+}\right]}\left[{\text{H}}_{2}{\text{O}}_{2}\right],$$

with a recommended temperature-dependent rate constant $k_{6\text{a}}=7.45\times 10^{7}\text{ exp}\left(-4000\left(\frac{1}{T}-\frac{1}{298}\right)\right)\text{L }{\text{mol}}^{-1}{\text{s}}^{-1}$ and *K*_5_ =13 mol L^−1^.

Recently, Liu et al. (2020) investigated S(VI) formation by S(IV) + H_2_O_2_ in a flow reactor under aqueous aerosol conditions (pH equal to 2.5, high ionic strength, and 73 %–90 % relative humidity) and in the presence of malonic acid. This study revealed that, under concentrated aqueous aerosol conditions, the S(VI) formation rate can be significantly increased compared to dilute aqueous conditions like those in clouds. The study demonstrated that ionic strength and general acid catalysis promotes faster S(VI) formation via Reaction (R6b). This additional pathway is expected to contribute to S(VI) missing from model simulations of severe haze episodes (Hering and Friedlander, 1982; Wang et al., 2014; P. He et al., 2018).

The rate expression given by Liu et al. (2020) is as follows:

(6b) $$R_{{\text{H}}_{2}{\text{O}}_{2}}=\left(k+k_{\text{HX}}[\text{HX}]{\left[{\text{H}}^{+}\right]}^{-1}\right)\times K_{\text{a}1}H_{{\text{SO}}_{2}}p_{{\text{SO}}_{2}}H_{{\text{H}}_{2}{\text{O}}_{2}}p_{{\text{H}}_{2}{\text{O}}_{2}}\;(\text{pH}>2),$$

with the following ionic strength dependencies of the reaction rate constant, Henry’s law constants, and dissociation constants (see Liu et al., 2020, and references therein). *k* is as follows:

(6c) $$\text{log}\left(\frac{k}{k^{I=0}}\right)=0.36\cdot I-\frac{1.018\sqrt{I}}{1+1.018\sqrt{I}}\;\left(I_{\text{max}}=5\text{ molal}\right).$$

$H_{{\text{H}}_{2}{\text{O}}_{2}}$ is as follows:

(6d) $$\frac{H_{{\text{H}}_{2}{\text{O}}_{2}}}{H_{{\text{H}}_{2}{\text{O}}_{2}}^{I=0}}=1-1.414\cdot 10^{-3}\cdot I^{2}+0.121\cdot I\;\left(I_{\text{max}}=5\text{ molal}\right).$$

$H_{{\text{SO}}_{2}}$ is as follows:

(6e) $$\frac{H_{{\text{SO}}_{2}}}{H_{{\text{SO}}_{2}}^{I=0}}=\left(\frac{22.3}{T}-0.0997\right)\cdot I\;\left(I_{\text{max}}=6\text{ molal}\right).$$

$K_{\text{a}1}^{*}$ is as follows:

(6f) $$\text{log}\left(\frac{K_{\text{a}1}^{*}}{K_{\text{a}1}^{I=0}}\right)=0.5\cdot \sqrt{I}-0.31\cdot I\;\left(I_{\text{max}}=6\text{ molal}\right),$$

and $K_{\text{a}2}^{*}$ is as follows:

(6g) $$\text{log}\left(\frac{K_{\text{a}2}^{*}}{K_{\text{a}2}^{I=0}}\right)=0.5\cdot \sqrt{I}-0.36\cdot I\;\left(I_{\text{max}}=6\text{ molal}\right).$$

In Eq. (6b), $k=k_{6\text{a}}\cdot 1.3\times 10^{-2}\cdot e^{1960(\frac{1}{T}-\frac{1}{298}})\cdot 6.6\times 10^{-8}.e^{1500\left(\frac{1}{T}-\frac{1}{298}\right)}$ (reaction rate constant of proton-catalyzed pathway Reaction R6a), *k*_HX_ (overall reaction rate constant of the catalysis pathway of a general acid HX – Reaction R6b; *k*_malonic acid_ = 5.61 × 10^5^ mol^2^ kg^−2^ s^−1^ (at *I* = 3.9 mol kg^−1^); *k*_malonate_ = 1.32 × 10^5^ mol^2^ kg^−2^ s^−1^ (at *I* = 6.6 mol kg^−1^)), $K_{\text{a}1}=1.3\times 10^{-2}\cdot {\text{e}}^{1960\left(\frac{1}{T}-\frac{1}{298}\right)}$ (thermodynamic dissociation constant of Reaction R5), $H_{{\text{SO}}_{2}}=1.23\cdot {\text{e}}^{3145.3\left(\frac{1}{T}-\frac{1}{298}\right)}$ (Henry’s law constant of SO_2_), and $H_{{\text{H}}_{2}{\text{O}}_{2}}=1.3\times 10^{5}\cdot {\text{e}}^{7297.1\left(\frac{1}{T}-\frac{1}{298}\right)}$ (Henry’s law constant of H_2_O_2_). Furthermore, $p_{{\text{SO}}_{2}}$ and $p_{{\text{H}}_{2}{\text{O}}_{2}}$ represent the partial pressure of SO_2_ and H_2_O_2_ in the gas phase, respectively. Note that the kinetics of the study by Liu et al. (2020) has been determined for NaCl–NaNO_3_-malonate/malonic acid mixtures only, which could restrict their applicability. Hence, further investigations for other aerosol composition mixtures (e.g., considering ammonium sulfate salts and other general acids), lower pH conditions, and higher ionic strengths are definitely needed to provide even more advanced rate expressions for concentrated aqueous aerosol conditions.

Organic hydroperoxides (ROOH) can also oxidize ${\text{HSO}}_{3}^{-}$ with a similar mechanism to that of H_2_O_2_, although at lower rates (Graedel and Goldberg, 1983; Lind et al., 1987; Drexler et al., 1991). The oxidation of ${\text{HSO}}_{3}^{-}$ by methylhydroperoxide, CH_3_OOH, has methanol as a product, with the overall reaction given as follows (Lind et al., 1987):

(R7) $${\text{HSO}}_{3}^{-}+{\text{CH}}_{3}\text{OOH}+{\text{H}}^{+}\to {\text{SO}}_{4}^{2-}+2{\text{H}}^{+}+{\text{CH}}_{3}\text{OH,}$$

with a third-order rate law as follows:

(7) $$R_{{\text{CH}}_{3}\text{OOH}}=k_{7}\left[{\text{HSO}}_{3}^{-}\right]\left[{\text{CH}}_{3}\text{OOH}\right]\left[{\text{H}}^{+}\right],$$

with $k_{7}=1.7\times 10^{7}\text{ exp}\left(-3800\left(\frac{1}{T}-\frac{1}{298}\right)\right){\text{L}}^{2}\;{\text{mol}}^{-2}{\text{s}}^{-1}$.

The S(IV) oxidation rate for peroxyacetic acid is faster (Lind et al., 1987), and produces acetic acid as a byproduct, thereby further increasing the acidity of the aqueous phase as follows:

(R8) $${\text{HSO}}_{3}^{-}+{\text{CH}}_{3}\text{C}(\text{O})\text{OOH}+{\text{H}}^{+}\to {\text{SO}}_{4}^{2-}+2{\text{H}}^{+}+{\text{CH}}_{3}\text{COOH,}$$

with a third-order rate law as follows:

(8) $$R_{{\text{CH}}_{3}\text{C}(\text{O})\text{OOH}}=k_{8}\left[{\text{HSO}}_{3}^{-}\right]\left[{\text{CH}}_{3}\text{C}(\text{O})\text{OOH}\right]\left[{\text{H}}^{+}\right],$$

with $k_{8}=5.6\times 10^{7}\text{ exp}\left(-3990\left(\frac{1}{T}-\frac{1}{298}\right)\right){\text{L}}^{2}\;{\text{mol}}^{-2}{\text{s}}^{-1}$.

The aerosol- and gas-phase abundances of organic hydroperoxides are poorly constrained, so S(IV) oxidation by ROOH may be more important than previously thought in aerosols containing secondary organic material (J. Ye et al., 2018; Dovrou et al., 2019; S. Wang et al., 2019). Organosulfates have been proposed as being minor products of the S(IV) + ROOH reactions with secondary organic material, with further implications for aerosol pH (S. Wang et al., 2019).

In contrast to S(IV) oxidation by H_2_O_2_, the oxidation of S(IV) by reaction with O_3_ becomes faster with increasing pH. Since S(VI) formation contributes to the acidification of the aerosol, these processes are, therefore, potentially self-limiting, depending on the buffering capacity of the aqueous medium (Fig. 6).

(R9a) $${\text{SO}}_{2}\cdot {\text{H}}_{2}\text{O}+{\text{O}}_{3}\to {\text{HSO}}_{4}^{-}+{\text{O}}_{2}+{\text{H}}^{+}$$

(R9b) $${\text{HSO}}_{3}^{-}+{\text{O}}_{3}\to {\text{HSO}}_{4}^{-}+{\text{O}}_{2}$$

(R9c) $${\text{SO}}_{3}^{2-}+{\text{O}}_{3}\to {\text{SO}}_{4}^{2-}+{\text{O}}_{2}.$$

Each S(IV) species reacts with O_3_, leading to a composite rate expression of the following:

(9) $$R_{{\text{O}}_{3}}=\left(k_{9\text{a}}\left[{\text{SO}}_{3}^{-2}\right]+k_{9\text{b}}\left[{\text{HSO}}_{3}^{-}\right]+k_{9\text{c}}\left[{\text{SO}}_{2}\cdot {\text{H}}_{2}\text{O}\right]\right)\times \left(1+F_{i}I\right)\left[{\text{O}}_{3}\right].$$

Here, *F*_*i*_ is an empirically determined factor accounting for the effect of ionic strength, *I*, on the rate. Lagrange et al. (1994) explored the effects of ionic strength on the oxidation of S(IV) by O_3_ (up to 4 mol L^−1^) and found that *F* = 1.59 ± 0.3 for NaCl and *F* = 3.71 ± 0.7 for Na_2_SO_4_. The rate constant for oxidation of ${\text{SO}}_{3}^{2-}$ by ${\text{O}}_{3}(k_{9\text{a}}=1.5\times 10^{9}\text{ exp}\left(-5280\left(\frac{1}{T}-\frac{1}{298}\right)\right)\text{L }{\text{mol}}^{-1}{\text{s}}^{-1})$ is over 3 orders of magnitude larger than the rate constant for ${\text{O}}_{3}+{\text{HSO}}_{3}^{-}\left(k_{9\text{b}}=3.7\times 10^{5}\text{ exp}\left(-5530\left(\frac{1}{T}-\frac{1}{298}\right)\right)\text{L }{\text{mol}}^{-1}{\text{s}}^{-1}\right)$ (Hoffmann and Calvert, 1985), which is more than 10 times the rate constant for the reaction of O_3_ with SO_2_ ·H_2_O (*k*_9c_ = 2.4 × 10^4^ L^2^ mol^−2^ s^−1^) when the respective maximum values are compared (Hoffman, 1986). Therefore, the overall rate of S(IV) oxidation by O_3_ increases rapidly with increasing pH and is most important above pH 5–6 (Chameides, 1984; Calvert et al., 1985; Turnock et al., 2019).

Sulfate can also form via a reaction of S(IV) with O_3_ on the surface of alkaline aerosols, e.g., freshly emitted sea salt aerosols and some mineral dust aerosols (Sievering et al., 1992; Chameides and Stelson, 1993; Zhang and Carmichael, 1999; Li et al., 2006; Wu et al., 2011; Yu et al., 2017; Zhang et al., 2018b). At pH values typical of fresh sea salt aerosol (pH ≈ 8), the S(IV) loss rate constant for oxidation by O_3_ in these aerosols is 10^5^ times larger than in-cloud oxidation by H_2_O_2_, more than making up for their lower liquid water content (Sievering et al., 1992; Chameides and Stelson, 1993). However, like other S(IV) + O_3_ mechanisms, these processes are potentially self-limiting, as noted above.

Besides S(IV) oxidation by H_2_O_2_ and O_3_, reactions of S(IV) with hypohalous acids (HOBr, HOCl, and HOI; see Reactions R17 and R18) contribute to sulfate formation in the marine boundary layer (Vogt et al., 1996; von Glasow et al., 2002a; Chen et al., 2016). These reactions act as a sink for reactive halogens by converting them to their acidic form (e.g., HOBr → HBr; see Sect. 4.8 for further details; Chen et al., 2016). It should be noted that the significance of these reactions is discussed in more detail in the dedicated section on halogen chemistry (see Sect. 4.8).

### 4.3 Free radical pathways for S(IV) oxidation

The hydroxyl radical (OH) can oxidize S(IV) in the aqueous phase through a radical pathway involving ${\text{SO}}_{3}^{-}$, ${\text{SO}}_{5}^{-}$, ${\text{HSO}}_{5}^{-}$, and ${\text{SO}}_{4}^{-}$. This process is more likely to be important in cloud water than in aqueous aerosol due to the higher liquid water content of clouds and the relatively lower OH concentration in aqueous aerosols (Herrmann et al., 2010; McNeill, 2015). The high concentrations of organic material in aerosols can quench radical and triplet species (Herrmann et al., 2010; McNeill, 2015; Wang et al., 2020). Furthermore, the reaction of OH with ${\text{SO}}_{3}^{2-}$ is somewhat faster than that of OH with ${\text{HSO}}_{3}^{-}$ (*k* = 4.6 × 10^9^ L mol^−1^ s^−1^ vs. 2.7 × 10^9^ L mol^−1^ s^−1^; Buxton et al., 1996). This, along with the pH dependence on the water solubility of SO_2_, suggests that S(IV) oxidation by OH is more efficient at higher pH and in clouds (and is potentially self-limiting). The production of ${\text{SO}}_{4}^{-}$ via this reaction pathway couples S(IV) oxidation to organosulfate production (Perri et al., 2010), although this is a minor pathway (McNeill et al., 2012).

Laboratory studies have demonstrated sulfate production on the surface of acidic aerosols via direct electron transfer from ${\text{HSO}}_{3}^{-}$ to O_2_, followed by a free radical chain oxidation of bisulfite to sulfate (Hung and Hoffmann, 2015); however, the significance of this pathway is not confirmed by field and modeling studies (Shao et al., 2019). Catalytic oxidation of S(IV) by NO_3_ (Exner et al., 1992; Rudich et al., 1998; Feingold et al., 2002), also believed to take place via a free radical mechanism, may be important in the remote troposphere. Recent experimental studies suggest that photolysis of particulate nitrate and hydrolysis of NO_2_ to form nitrate and HONO (Li et al., 2018) may accelerate the oxidation of S(IV) under Beijing conditions by generating NO_2_ and OH radicals (Gen et al., 2019). However, the consumption of OH radicals by organic constituents present in aerosols was ignored in this study, likely leading to an overestimation of the effect.

Another suggested S(IV) oxidation pathway is the reaction of excited triplet states of photosensitizers (PS*) with S(IV) species (see Reaction R10). This pathway potentially involves produced sulfur-containing radicals and/or excited transient species (see, e.g., Wang et al., 2020, and Loeff et al., 1993). Currently, it is also being discussed as a potential S(IV) oxidation pathway under polluted aerosol conditions (Wang et al., 2020).

(R10) $$\text{S}(\text{IV})+{\text{PS}}^{*}\to \text{S}(\text{VI})+\text{products }\left(\text{S}(\text{IV})={\text{SO}}_{2}\cdot {\text{H}}_{2}\text{O}+{\text{HSO}}_{3}^{-}\right).$$

The exact reaction pathway is still uncertain, particularly with respect to the involved sulfur-containing radicals or excited transient species. Some studies (Loeff et al., 1993; Wang et al., 2020) already determined the chemical reaction rate constants for certain PS* species, such as acetophenone, flavone, xanthone, 4-(benzoyl)benzoic acid, and anthraquinone-1-sulfonate. The second-order reaction rate constants of PS* with S(IV) species measured in the laboratory are between 6.0 × 10^7^ and 1.0 × 10^9^ mol L^−1^ s^−1^. Kinetic measurements of the reactive PS* quenching by S(IV), using ambient filter extracts taken during Chinese winter haze conditions, revealed a rate constant of 1.3 × 10^8^ mol L^−1^ s^−1^ (Wang et al., 2020). Note that the kinetic investigations of Wang et al. (2020) assumed that the initial reaction step is the rate-limiting step in this reaction sequence, and the reaction rate constant is pH independent. So, based on Wang et al. (2020), the rate expression is as follows:

(10) $$R_{{\text{PS}}^{*}}=k_{10}\left[{\text{PS}}^{*}\right][\text{S}(\text{IV})].$$

Due to the presently strong uncertainties in the existing kinetic data and mechanistic understanding of the Reaction (R10), a recommendation of a proper kinetic reaction rate constant is rather difficult. Thus, we preliminarily recommend the chemical rate constant of *k*_10_ = 1.3 × 10^8^ mol L^−1^ s^−1^. Finally, it should be noted that great care is needed when estimating the rate of Reaction (R10) because of (i) lacking knowledge about the present PS* concentrations in ambient aerosols and cloud droplets, as well as (ii) the very rapid quenching and deactivation triplet species by water, dissolved oxygen, as well as organic and inorganic aerosol constituents. The latter might lead to very low PS* concentrations, which can strongly limit or inhibit this pathway (similarly to the S(IV) oxidation by free radicals). This oxidation pathway can be effectively inhibited by particle constituents other than S(IV), as described earlier in the present section.

### 4.4 S(IV) oxidation catalyzed by transition metal ions

The oxidation of S(IV) by O_2_, as catalyzed by transition metal ions (TMI; mainly Fe(III) and Mn(II); see Reactions R11 and R12; Humphreys, 1964; Martin and Hill, 1987b, a; Brandt and van Eldik, 1995; Alexander et al., 2009; Harris et al., 2013), is an efficient pathway for S(VI) formation, especially under conditions where photochemistry is limited, e.g., wintertime at high latitudes (Simpson et al., 2019).

(R11) $$\text{S}(\text{IV})+\frac{1}{2}{\text{O}}_{2}\overset{\text{Fe}(\text{III})}{\to }\text{S}(\text{VI})$$

(R12) $$\text{S}(\text{IV})+\frac{1}{2}{\text{O}}_{2}\overset{\text{Mn}(\text{II})}{\to }\text{S}(\text{VI}).$$

The solubility and speciation of the TMI (Deguillaume et al., 2005), as well as the reaction rates, all depend on pH. As primary pollutants, TMI concentrations are higher in aerosols than in cloud water, but this effect is limited by the pH-dependent solubility of the active species. The TMI-S(IV) reactions (Reactions R11 and R12) are also reported to be inhibited by ionic strength (Martin and Hill, 1987b, a), although this dependence is only known under relatively diluted conditions which are accessible in bulk solutions. This introduces considerable additional uncertainty to estimates of the aerosol-phase TMI catalyzed S(IV) oxidation rate.

TMI-mediated S(IV) oxidation has been proposed to proceed through radical intermediates (Grgić and Berčič, 2001), at least for pH > 3.6 (Martin et al., 1991). A detailed discussion of the mechanisms can be found in Brandt and van Eldik (1995) and Rudziński et al. (2009). A pH-dependent synergistic effect has been reported when multiple transition metal ions are present in a solution (Ibusuki and Takeuchi, 1987; Martin and Good, 1991; Harris et al., 2013). Martin et al. (1991) observed that water-soluble organic material inhibits Fe(III)-catalyzed S(IV) oxidation for pH ≥ 5. Given this pH range, the effect is not expected to be significant for atmospheric aerosols, although interactions with organics, for example complexation with oxalate, may impact TMI chemistry in other ways (e.g., Okochi and Brimblecombe, 2002; Passananti et al., 2016).

Given the current focus on sulfate formation in atmospheric aerosols, our recommendations for the kinetics of S(IV) oxidation by TMI favor studies which included the ionic strength and pH effects. For Fe(III)-catalyzed S(IV) oxidation, the expression from Martin and Hill (1987a) and Martin et al. (1991) is as follows:

(11a) $$R_{\text{Fe},10\text{a}}=\{\begin{array}{ll} k_{11\text{a}}\frac{[\text{Fe}(\text{III})][\text{S}(\text{IV})]10^{-2\sqrt{\text{I}}/(1+\sqrt{\text{I}})}}{\left[{\text{H}}^{+}\right]\left(1+K_{11}{\left[\text{S}(\text{VI})\right]}^{2/3}\right)} & \text{for pH}<3.6\\ k_{11\text{b}}{\left[\text{Fe}(\text{III})\right]}^{2}[\text{S}(\text{IV})] & \text{for }3.6\leq \text{pH}\leq 5\\ k_{11\text{c}}[\text{S}(\text{IV})] & \text{for }5<\text{pH}\leq 6\\ k_{11\text{d}}[\text{S}(\text{IV})] & \text{ for pH}>6 \end{array}.$$

Here, *k*_11a_ = 6 s^−1^, *K*_11_ = 150 (mol L^−1^)^−2/3^, *k*_11b_ = 10^9^ L^2^ mol^−2^ s^−1^, *k*_11c_ = 10^−3^ s^−1^, and *k*_11d_ = 10^−4^ s^−1^. However, the dependence of Eq. (11a) on ionic strength (*I*) is only known up to 1 mol L^−1^, and unfortunately, the rate law is valid for a limited range of conditions only ([Fe^3+^] > 10^−7^ mol L^−1^, [S(IV)] < 10^−5^ mol L^−1^, [S(VI)] < 10^−4^ mol L^−1^, and *I* < 10^−2^ mol L^−1^). Moreover, note that the ionic strength effect was verified at pH = 2 and *T* = 25°C only. Additionally, the study implied that the effect of higher S(IV) and S(VI) concentrations may be more important than the ionic strength effect (see Martin et al., 1991, for details). Due to the limited range of conditions in which the expressions of Martin and Hill (1987a) and Martin et al. (1991) are valid and the existing gaps in the understanding of this reaction, we recommend the rate expression by Hoffmann and Calvert (1985).

(11b) $$R_{\text{Fe},11\text{b}}=k_{10\text{e}}[\text{Fe}(\text{III})]\left[{\text{SO}}_{3}^{2-}\right]\;(\text{for pH}<5),$$

with *k*_10e_ = 1.2 × 10^6^ L mol^−1^ s^−1^.

The rate for Mn(II)-catalyzed S(IV) oxidation from Martin and Hill (1987b) is recommended as follows:

(12) $$R_{\text{Mn}}=\{\begin{array}{ll} k_{12\text{a}}[\text{Mn}(\text{II})][\text{S}(\text{IV})] & \text{for S }(\text{IV})<10^{-4}\text{mol }{\text{L}}^{-1}\\ k_{12\text{b}}{\left[{\text{Mn}}^{2+}\right]}^{2} & \text{for S }(\text{IV})>10^{-4}\text{mol }{\text{L}}^{-1} \end{array}$$

where $k_{12\text{a}}=k_{12\text{a},0}10^{-4.07\sqrt{I}/(1+\sqrt{I})}\text{L }{\text{mol}}^{-1}{\text{s}}^{-1}$ and $k_{12\text{b}}=k_{12\text{b},0}10^{-4.07\sqrt{I}/(1+\sqrt{I})}\text{L }{\text{mol}}^{-1}{\text{s}}^{-1}$, with *k*_12a,0_ = 10^3^ L m^−1^ s^−1^ and *k*_11b,0_ = 680 L mol^−1^ s^−1^. Note that, while Martin and Hill (1987a, b) observed strong inhibition with increasing ionic strength, *k*_12a_ is only reported for ionic strength up to 1 mol L^−1^. Overall, TMI-catalyzed reactions are still not very well understood, and further studies of these reactions particularly under aerosol conditions are needed.

A synergistic effect has been reported in laboratory studies when Fe(III) and Mn(II) are both present in a solution (Martin, 1984; Ibusuki and Takeuchi, 1987; Martin and Good, 1991; Grgić et al., 1992), but more work must be done to reconcile the rates of Reaction (R13) from those studies with single-ion studies, and the effect of ionic strength is not known.

(R13) $$\text{S}(\text{IV})+1/2{\text{O}}_{2}\overset{\text{Fe}(\text{III})+\text{Mn}(\text{II})}{\to }\text{S}(\text{VI}).$$

The recommended rate of Reaction (R13) is from Ibusuki and Takeuchi (1987), who investigated the effect as a function of pH and temperature:

(13) $$R_{\text{TMI}-\text{Syn}}=\{\begin{array}{ll} k_{13\text{a}}{\left[{\text{H}}^{+}\right]}^{-0.74}\;[\text{Mn}(\text{II})][\text{Fe}(\text{III})][\text{S}(\text{IV})] & \text{for }2.6\leq \text{pH}\leq 4.2\\ k_{13\text{b}}{\left[{\text{H}}^{+}\right]}^{0.67}\;[\text{Mn}(\text{II})][\text{Fe}(\text{III})][\text{S}(\text{IV})] & \text{for }4.2<\text{pH}\leq 6.5 \end{array},$$

where *k*_13a_ = 3.72 × 10^7^ L mol^−1^ s^−1^, and *k*_13b_ = 2.51 × 10^13^ L mol^−1^ s^−1^.

A more comprehensive literature overview on reaction rate constants related to TMI-catalyzed S(IV) oxidation kinetics is given in Radojevic (1992) and Brandt and van Eldik (1995).

### 4.5 NO_2_ and HNO_4_

NO_2_ can oxidize ${\text{HSO}}_{3}^{-}$ in the aqueous phase (Lee and Schwartz, 1983) through adduct formation, followed by decomposition, to eventually form ${\text{SO}}_{3}^{-}$ and the weak acid HONO. The thermodynamic driving force for this process is small (Spindler et al., 2003). The reaction favors basic conditions and, therefore, is unlikely to be significant for most atmospheric aerosols and self-limiting. Early studies by Lee and Schwartz (1983) reported relatively high reaction rates which decreased rapidly with decreasing pH. Spindler et al. (2003) demonstrated that, based on coupled gas- and aqueous-phase measurements together with the direct measurement of NO_2_ in an aqueous solution, the reaction between NO_2_ and S(IV) proceeds first by an adduct formation equilibrium (Reactions R14a and R14b), followed by the adduct’s unimolecular decomposition (Reactions R15a and R15b) to the products of nitrite and ${\text{SO}}_{3}^{-}$.

(R14a) $${\text{NO}}_{2(\text{aq})}+{\text{SO}}_{3(\text{aq})}^{2-}\underset{k_{-14\text{a}}}{\overset{k_{14\text{a}}}{⇌}}{\left[{\text{NO}}_{2}-{\text{SO}}_{3}\right]}^{2-}$$

(R15a) $${\left[{\text{NO}}_{2}-{\text{SO}}_{3}\right]}^{2-}\overset{k15\text{a}}{\to }{\text{NO}}_{2}^{-}+{\text{SO}}_{3(\text{aq})}^{-}$$

(R14b) $${\text{NO}}_{2(\text{aq})}+{\text{HSO}}_{3(\text{aq})}^{-}\underset{k_{-14\text{b}}}{\overset{k_{14\text{b}}}{⇌}}{\left[{\text{NO}}_{2}-{\text{HSO}}_{3}\right]}^{-}$$

(R15b) $${\left[{\text{NO}}_{2}-{\text{HSO}}_{3}\right]}^{2-}\overset{k15\text{b}}{\to }{\text{H}}^{+}+{\text{NO}}_{2}^{-}+{\text{SO}}_{3(\text{aq})}^{-}.$$

This mechanism (Reactions R14a–R15b) was invoked to explain the formation of “artifact HONO” in a wet denuder when both NO_2_ and SO_2_ are present in the ambient gas phase. The study of Spindler et al. (2003) aimed at measuring gas-phase HONO. However, chemical interactions of dissolved NO_2_ and SO_2_ at wetted denuder walls can lead to the formation of the two long-lived intermediates of [NO_2_ − SO_3_]^2−^ and [NO_2_ − HSO_3_]^−^ (see Reactions R14a and R14b), which decay into ${\text{NO}}_{2}^{-}$ and ${\text{SO}}_{3}^{2-}$, respectively. In order to quantify this artificial HONO formation and, subsequently, correct the measured HONO, kinetic data of this reaction system (Reactions R14a–R15b) were experimentally determined in the study of Spindler et al. (2003) by measuring NO_2_ in aqueous solution with a laser photolysis broadband optical absorption experimental setup. For this review, the kinetic data of Spindler et al. (2003) have again been kinetically analyzed in more detail. The measurements of Spindler et al. (2003) were performed at pH = 4.5 and pH = 10 to investigate either the ${\text{HSO}}_{3}^{-}$ or the fully deprotonated form ${\text{SO}}_{3}^{2-}$. From the *T* -dependent rate constants (see Table S2) of the forward (*k*_14a_, *k*_14b_) and backward reaction (*k*_−14a_, *k*_−14b_), the equilibrium constants (*K*_14a_, *K*_14b_) were calculated, and the Arrhenius expressions were derived at pH 10.0, as follows:

- *k*_14a_(*T*) = (1.4 ± 0.2)10^7^ L mol^−1^ s^−1^ (288 K ≤ *T* ≤ 328 K)
- *k*_−14a_(*T*) = (3.5 ± 0.5)10^6^ exp[−(2440 ± 710) *K*/*T*]s^−1^
- *K*_14a_(*T*) = (1.9 ± 15) exp[−(−2700 ± 1600)*K*/*T*] L mol^−1^,

and at pH 4.5, as follows:

- *k*_14b_(*T*) = (8.5 ± 1.9)10^12^ exp[−(4670 ± 2010)*K*/*T*] L mol^−1^ s^−1^
- *k*_−14b_(*T*) = (3.8 ± 0.5)10^7^ exp[−(3560 ± 680)*K*/*T*]s^−1^
- *K*_14b_(*T*) = (2.2 ± 0.1)10^5^ exp[−(2270 ± 150)*K*/*T*] L mol^−1^ (298 K ≤ *T* ≤ 328 K).

Finally, from the measurements of artifact HONO in the Spindler et al. (2003) publication, the unimolecular rate of decomposition for the adduct was determined as *k*_15a_(*T*) = (8.4 ± 0.1) 10^−3^ s^−1^ (*T* = 298 K).

The most significant difference between the results of Spindler et al. (2003) and earlier studies is that the mechanism identified by Spindler et al. (2003) includes the adduct formation with a slow adduct decomposition (see Reactions R14a–R15b), which considerably limits the potential for S(VI) formation via this mechanism under environmental conditions. Here, from the viewpoint of aqueous-phase thermochemistry, it should also be noted that such high rate constants for a prompt bimolecular reaction with a concerted single electron transfer from ${\text{HSO}}_{3}^{-}$ to NO_2_ would not be feasible. The one-electron reduction potentials of NO_2(aq)_ and ${\text{HSO}}_{3(\text{aq})}^{-}$ are very similar, with $\text{E}{}^{\circ}\left({\text{SO}}_{3}^{-}/{\text{HSO}}_{3}^{-}\right)=0.84\text{ V}$ vs. NHE at pH = 3.6 (Huie and Neta, 1984) and $\text{E}{}^{\circ}\left({\text{NO}}_{2}/{\text{NO}}_{2}^{-}\right)=1.04\pm 0.02\text{ V}$ vs. NHE (Armstrong et al., 2013), and, as a consequence, a fast reaction would not be in line with the very limited energetic driving force of the reaction as its Gibbs free enthalpy of reaction. For comparison, the redox potential $\text{E}{}^{\circ}\left({\text{SO}}_{3}^{-}/{\text{SO}}_{3}^{2-}\right)$ is 0.63 V vs. NHE at pH > 7 (Huie and Neta, 1984; Wardman, 1989), implying a faster reaction rate at higher pH.

The oxidation of S(IV) by NO_2_ in aerosol water was previously proposed to be important during wintertime haze episodes in Beijing (Cheng et al., 2016; Wang et al., 2016). The significance of this S(IV) oxidation pathway rests on (a) the hypothesis that aerosols in Beijing have an unusually high pH of about 7 (Wang et al., 2016), which is not supported by thermodynamic models (see Pye et al., 2020; with an average pH value of approximately 4 for China), and (b) the mechanism and relatively fast kinetic parameters of earlier studies by Lee and Schwartz (1983) and Clifton et al. (1988), without considering the more recent findings of Spindler et al. (2003) and the underlying thermochemistry. For completeness, the significantly different S(VI) rates resulting from the different kinetic parameters of Lee and Schwartz (1983), Clifton et al. (1988), and Spindler et al. (2003), considering the NO_2_ and SO_2_ conditions for wintertime haze conditions based on Cheng et al. (2016), are shown in Fig. S1 in the Supplement.

Recent isotopic studies provide further evidence that this reaction is not important in Beijing (Au Yang et al., 2018; P. He et al., 2018; Shao et al., 2019; Li et al., 2020a), which is in line with the aforementioned mechanistic and thermodynamic considerations.

The importance of the ${\text{NO}}_{2}+{\text{HSO}}_{3}^{-}$ reaction has also been highlighted for fogs in China, with pH > 5 (Xue et al., 2016, 2019). However, as with the aerosol aqueous chemistry, this sulfate production pathway should be self-limiting due to its production of H^+^.

Peroxynitric acid (HNO_4_), a product of the gas-phase reaction of HO_2_ and NO_2_, also oxidizes ${\text{HSO}}_{3}^{-}$, primarily in cloud water, with a rate constant of 3.3 × 10^5^ L mol^−1^ s^−1^ (Amels et al., 1996; Warneck, 1999; Dentener et al., 2002). The reaction rate increases with increasing aqueous pH due to the increased solubility of S(IV) and HNO_4_. Besides the acidifying effect of S(IV) to S(VI) conversion, the reaction yields nitric acid (HNO_3_) as an acidic byproduct. The significance of this pathway depends on gas-phase HO_*x*_ and NO_*x*_ levels and the relative abundance of other competing S(IV) oxidants.

### 4.6 Overall S(IV) oxidation considerations

**Figure 7.**
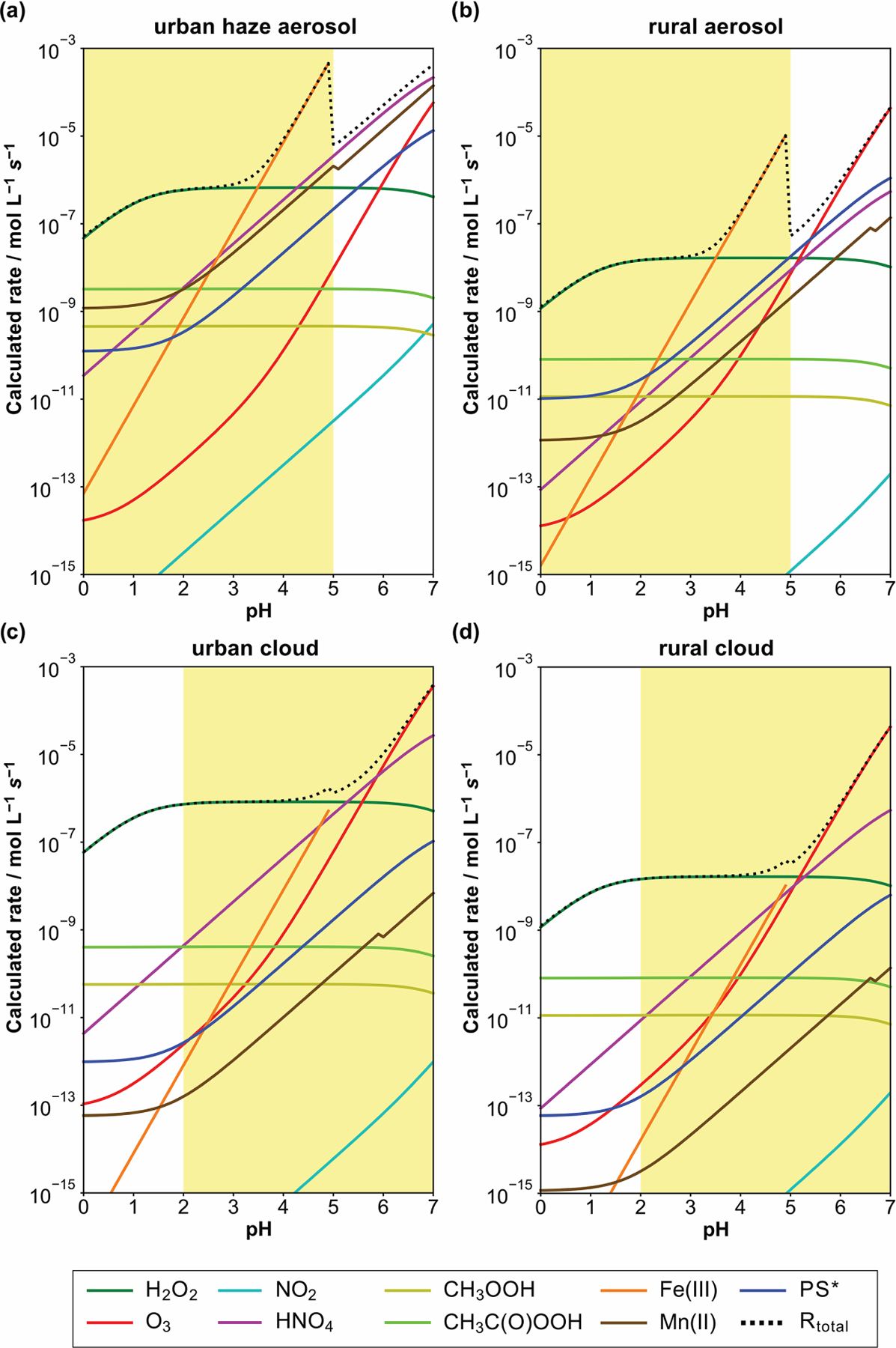
Calculated S(IV) oxidation rates of different reaction pathways in mol L^−1^ s^−1^ for urban winter haze (**a**) and rural aerosol (**b**) conditions, as well as urban (**c**) and rural (**d**) cloud conditions at 298 K. Applied conditions are given in Table 1, and the rate expressions used were those given in this text. The atmospherically relevant acidity range in the different cases is marked in yellow.

To compare the potential atmospheric relevance of the different S(IV) to S(VI) conversion pathways with respect to different environmental and acidity regimes in aerosols, haze, and clouds, initial S(IV) oxidation rates of the different pathways discussed up to this point were calculated. Figure 7 shows the resulting calculated S(IV) oxidation rates of these reaction pathways in moles per liter water per second (hereafter, mol L^−1^ s^−1^) for continental urban haze and rural aerosol conditions, as well as continental urban and rural cloud conditions. These rates were calculated with the rate expressions from the subsections above (Eqs. 6a, 7, 8, 9, 10, 11b, and 12) and are based on the typical conditions as summarized in Table 1. For the NO_2_, kinetic rates were calculated applying the pseudo-steadystate approximation ($k_{\text{PSSA},{\text{HSO}}_{3}^{-}}=1.3\times 10^{1}\text{ L }{\text{mol}}^{-1}{\text{s}}^{-1}$; $k_{\text{PSSA},{\text{SO}}_{3}^{2-}}=2.7\times 10^{2}\text{ L }{\text{mol}}^{-1}{\text{s}}^{-1}$). For HNO_4_, the reaction rate was calculated with a rate constant of 3.3 × 10^5^ L mol^−1^ s^−1^ (Amels et al., 1996; Warneck, 1999; Dentener et al., 2002). For Fe(III) and Mn(II), the rate expressions by Hoffmann and Calvert (1985) and Martin and Hill (1987b) were applied, respectively. Note that the synergistic rates of Ibusuki and Takeuchi (1987) (Eq. 13) were not used due to the still large uncertainties in this oxidation pathway.

For diluted aqueous solution (cloud) conditions, the S(IV) oxidation by dissolved H_2_O_2_, O_3_, HNO_4_, and the iron-catalyzed pathway are the most important oxidation pathways (see Fig. 7c and d). The reaction with dissolved H_2_O_2_ is the major oxidation pathway under acidic cloud conditions. Under less acidic cloud conditions (pH > 5), the other reaction pathways are able to contribute significantly to the S(VI) formation. Figure 7 also shows that the oxidation rates of other oxidants, such as NO_2_, excited triplet states of photosensitizers (PS*), and organic hydroperoxides (CH_3_COOH and CH_3_C(O)OOH), are unimportant under cloud conditions, mainly because of their low in-cloud concentrations.

On the other hand, under more concentrated aqueous solution conditions (haze and deliquesced aerosol), the molar concentrations of TMIs are significantly higher. Thus, the contributions of TMI-catalyzed S(IV) oxidation pathways are elevated against cloud conditions. From the calculation output in Fig. 7a and b, it can be seen that the S(IV) oxidation by dissolved H_2_O_2_ is still predominant below pH ≤ 3. However, already at quite low acidity conditions with pH ≈ 3.5, the TMI-catalyzed pathways can become the main oxidation route for S(IV). Note that the synergistic rate of Ibusuki and Takeuchi (1987) (Eq. 13) was not included in the current study, so even higher contributions of TMI-catalyzed S(IV) oxidation pathways can be possible. Moreover, it should be noted that the S(IV) oxidation rates in Fig. 7a and b appear a bit unnatural because of the applied constants of the S(IV) oxidation by Fe(III) (Hoffmann and Calvert, 1985) as reported in Eq. (11b). This rate expression is only valid for pH conditions < 5. However, the efficiency of the iron(III)-catalyzed oxidation of S(IV) to S(VI) strongly depends on speciation of iron(III), i.e., the concentration of inorganic and organic complexing agents (see Deguillaume et al., 2005), which is not considered in the rate intercomparison. At higher pH values, the *pK*_a_ values of important complexing agents are exceeded. Accordingly, these compounds will be present in their dissociated forms, thus enabling a stronger iron(III) complexation and inhibiting the iron-catalyzed S(IV) oxidation. This strong inhibiting effect on iron(III)-catalyzed S(IV) oxidation is well known, for example, for organic acids such as oxalate (see, e.g., Grgić et al., 1998). Thus, the iron(III)-catalyzed S(IV) oxidation becomes less important at pH > 5 as many *pK*_a_ values of organic acids are typically < 5.

Figure 7a and b show that, besides the TMI-catalyzed S(IV) oxidation pathways, S(IV) oxidations by dissolved HNO_4_ and O_3_, as well as, to some extent, PS*, can also be important under polluted haze and rural aerosol conditions when pH > 5. Importantly, the current comparison clearly shows that the NO_2_-driven S(IV) oxidation route, even under very high NO_*x*_ conditions (66 ppb) applied in the urban haze case, still remains of minor importance. Only by the combination of applying unusually high aerosol pH values, artificially low H_2_O_2_ and O_3_ concentrations, and unrealistically fast kinetic parameters from earlier studies by Clifton et al. (1988; see Sect. 4.5 above) can NO_2_ rates fall into the range of other key oxidants discussed here (see Cheng et al., 2016). In detail, the used H_2_O_2_ and O_3_ concentrations of 0.01 and 1 ppb, used by Cheng et al. (2016) for urban haze conditions, are far too low. Recent measurements of H_2_O_2_ and O_3_ concentrations under haze conditions in the North China Plain (C. Ye et al., 2018; Fan et al., 2020; Ye et al., 2021) showed substantially higher values of about 0.5 and 10 ppb, respectively.

In conclusion, the outcomes of this comprehensive comparison are in agreement with the findings of isotope field investigations (see, e.g., Harris et al., 2013; Au Yang et al, 2018; P. He et al., 2018; Shao et al., 2019; Li et al., 2020a; Hattori et al., 2021; Wang et al., 2021) which have implied that mainly H_2_O_2_, O_3_, and TMI-catalyzed pathways are responsible for the S(IV) to S(VI) conversion in atmospheric aqueous-phase cloud and aerosol solutions. However, due to the uncertainties still existing with regard to kinetics and mechanisms, further acidity-dependent investigations appear warranted.

### 4.7 Sequestering of S(IV) as hydroxymethanesulfonate

${\text{HSO}}_{3}^{-}$ or ${\text{SO}}_{3}^{-2}$ can react with a variety of aldehydes to form hydroxyalkylsulfonates (Olson and Hoffmann, 1989). Of particular interest has been the S(IV) reaction with HCHO to produce hydroxymethanesulfonate (HMS; ${\text{HOCH}}_{2}{\text{SO}}_{3}^{-}$; Munger et al., 1986). The formation of HMS is strongly dependent on drop acidity, which increases rapidly at higher pH values due to the increased partitioning of S(IV) to ${\text{HSO}}_{3}^{-}$ and ${\text{SO}}_{3}^{2-}$ (Rao and Collett, 1995). Furthermore, the reaction rate increases with increasing pH due to the fact that the rate coefficient for ${\text{SO}}_{3}^{2-}$ (*k* = 2.5 × 10^7^ L mol^−1^ s^−1^) is more than 4 orders of magnitude higher than that for ${\text{HSO}}_{3}^{-}$ (*k* =790 L mol^−1^ s^−1^; Boyce and Hoffmann, 1984; Olson and Hoffmann, 1989). At pH values > 6, HMS formation becomes so fast that it can limit aqueous sulfate production in large droplets where mass transport limits SO_2_ uptake from the gas phase (Reilly et al., 2001). Since the oxidation of HMS is slow (Hoigne et al., 1985; Kok et al., 1986; Barlow et al., 1997b, a), its formation effectively protects S(IV) from oxidation to S(VI) by non-radical oxidants such as H_2_O_2_, O_3_, and others. Whiteaker and Prather (2003) demonstrated the utility of HMS measurements in single particles as a tracer for fog processing. Recent field and modeling studies have suggested that HMS production may also be an important contributor to fine particle sulfur content under polluted haze conditions (Moch et al., 2018; Song et al., 2019b; Ma et al., 2020; Moch et al., 2020). Sulfur in particles may exist in the form of other sulfonates $\left({\text{R-C-SO}}_{3}^{-}\right)$ besides organosulfates (${\text{R-C-O-SO}}_{3}^{-}$; Le Breton et al., 2018; Brüggemann et al., 2020).

### 4.8 Acid-driven production of tropospheric reactive halogens: multiphase halogen activation

**Figure 8.**
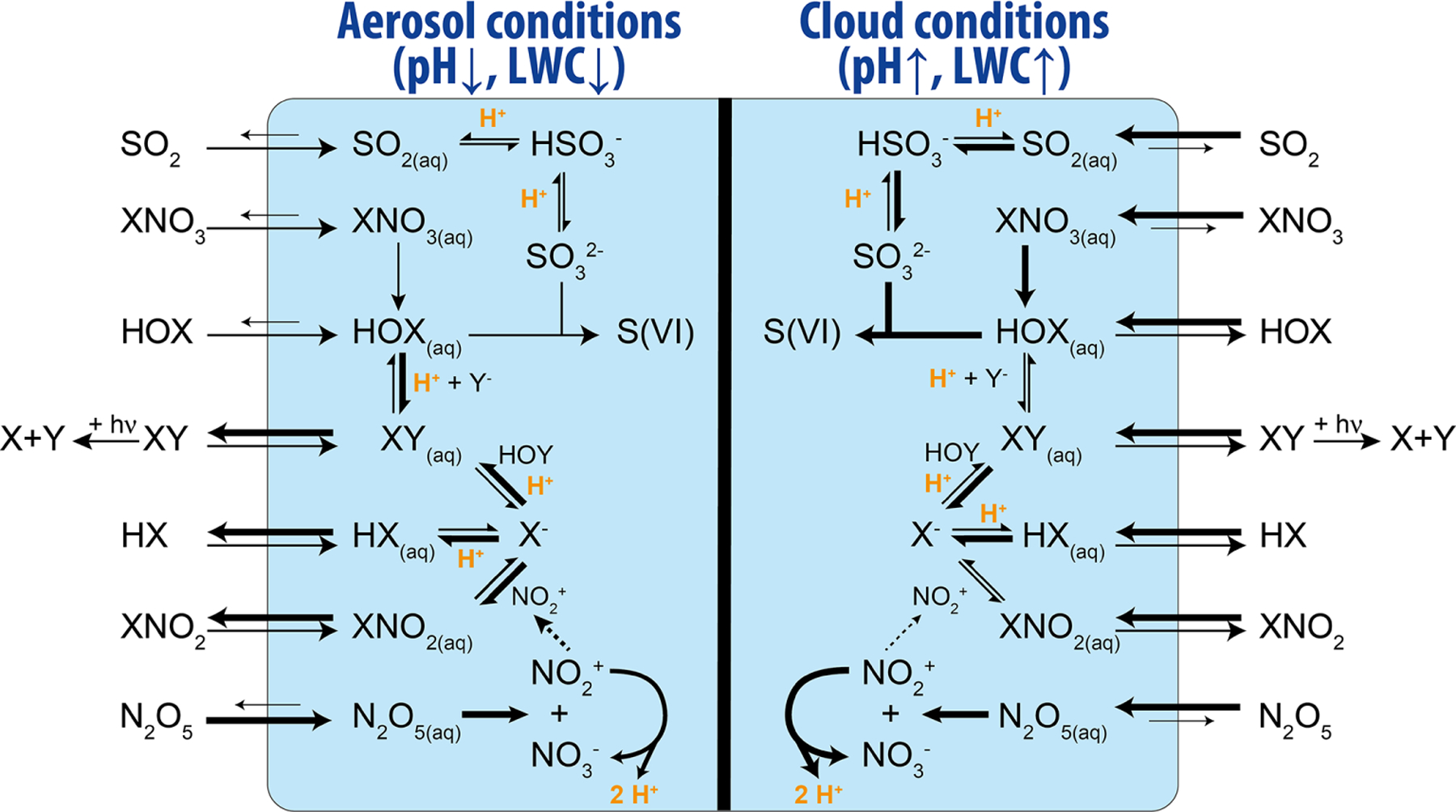
Simplified scheme of the reactive halogen chemistry and their differences between diluted less acidic cloud conditions and more concentrated and acidic aerosol conditions. Differences in the chemical rates and uptake fluxes are indicated by thinner and thicker arrows, respectively.

Of the many acid-catalyzed reactions in the atmosphere, the acid-catalyzed formation of reactive halogens (Br, Cl, and I) in the troposphere has the potential to render acidity as an influencer of the oxidative capacity of the atmosphere, although its influence has yet to be fully quantified. Reactive halogens and halogen reservoir species are of the form Br_*y*_ (= Br + 2Br_2_ + HOBr + BrO + HBr + BrNO_2_ + BrNO_3_ + IBr + BrCl), Cl_*y*_ (= Cl + 2Cl_2_ + HOCl + ClO + HCl + ClNO_2_ + ClNO_3_ + ICl + BrCl + ClOO + OClO + 2Cl_2_O_2_), and I_*y*_ (I + 2I_2_ + HOI + IO + OIO + HI + HIO_3_ + INO + INO_2_ + INO_3_ +2I_2_O_2_ + 2I_2_O_3_ + 2I_2_O_4_). Tropospheric reactive halogens can impact the oxidation capacity of the atmosphere by (i) acting as an effective sink for ozone (O_3_), e.g., during bromine explosion events in the Arctic, (ii) acting as an effective sink for nitrogen oxides (NO_*x*_ = NO + NO_2_), and (iii) by influencing the HO_*x*_ (= OH + HO_2_) (Oltmans et al., 1989; Simpson et al., 2015; Schmidt et al., 2016; Sherwen et al., 2016; Hoffmann et al., 2019a). Reactive halogens also directly impact the lifetime of reduced trace gases such as methane (CH_4_) and non-methane volatile organic compounds (VOCs), dimethylsulfide (DMS), and mercury in the atmosphere (Barnes et al., 2006; Saiz-Lopez and von Glasow, 2012; Ariya et al., 2015; Simpson et al., 2015). Sources of tropospheric reactive halogens include the oxidation of organohalogens (e.g., CH_3_Br and CH_3_I; Saiz-Lopez et al., 2012b; Saiz-Lopez and von Glasow, 2012), deposition of ozone to the ocean surface to yield HOI and I_2_ (Carpenter et al., 2013), release from sea salt aerosols (Parrella et al., 2012; Schmidt et al., 2016; Sherwen et al., 2017), and, to a minor extent, transport from the stratosphere (Schmidt et al., 2016; X. Wang et al., 2019). Liberation of halogens to their reactive form via acid-catalyzed reactions on sea salt aerosols (see Fig. 8) is the largest source of reactive bromine in the troposphere (Vogt et al., 1996; von Glasow et al., 2002a; Pechtl et al., 2007; Pechtl and von Glasow, 2007; Parrella et al., 2012; Chen et al., 2017). As shown in Fig. 8, the formation and processing of reactive halogens strongly depends on the aqueous-phase conditions, i.e., the LWC and the acidity of the solution.

Formation of reactive halogens (Br, Cl, and I) from sea salt aerosols proceeds in pristine environments via the uptake of hypohalous acid species (HOX; where X is equal to Br, Cl, or I) from the gas phase (von Glasow et al., 2002b) or in more polluted environments via the hydrolysis of N_2_O_5_ forming ClNO_2_ (Finlayson-Pitts et al., 1989; Roberts et al., 2009; Sarwar et al., 2014), as well as via the hydrolysis of XNO_3_ forming HOX (Schmidt et al., 2016; Hoffmann et al., 2019b). See Reactions (R16a)–(R16c) as follows:

(R16a) $${\text{HOX}}_{\left(\text{aq}\right)}+{\text{Y}}^{-}+{\text{H}}^{+}\to {\text{XY}}_{\left(\text{aq}\right)}+{\text{H}}_{2}\text{O}$$

(R16b) $${\text{N}}_{2}{\text{O}}_{5(\text{aq})}+{\text{Cl}}^{-}\to {\text{ClNO}}_{2(\text{aq})}+{\text{NO}}_{3(\text{aq})}^{-}$$

(R16c) $${\text{XNO}}_{3(\text{aq})}+{\text{H}}_{2}{\text{O}}_{\left(\text{aq}\right)}\to {\text{HOX}}_{\left(\text{aq}\right)}+{\text{HNO}}_{3(\text{aq})},$$

where X equals Br, Cl, or I. If two different halogens are involved, Y denotes the second halogen atom. The formed XX or XY species then either reacts further or partitions to the gas-phase, where it is photolyzed and participates in gas-phase oxidation chemistry to ultimately regenerate HOX or XNO_3_. Nonlinear reactive halogen production proceeds via the uptake of one molecule HOX or XNO_3_, by carrying one halogen atom and yielding two halogen atoms released back to the gas phase (see Fig. 8). Note that this is an acid-driven process which consumes H^+^ in the aqueous-particle phase without recycling it, and one halogen anion Y^−^ is also consumed. When Br is a participant, this auto-catalytic reaction cycle can lead, under high bromide concentrations, to so-called bromine explosion events characterized by high concentrations of BrO (Evans et al., 2003) resulting from the gas-phase reaction of Br with O_3_.

Changing atmospheric acidity due to changes in anthropogenic emissions of acid precursor gases may influence the formation of reactive halogens via Reaction (R16a) (Keene et al., 1998). However, lower acidity conditions might also result in the stronger aqueous-phase partitioning of hydrogen halides, which might partly compensate for the reduced acidity effect via Reaction (R16a). Changes in sulfur dioxide (SO_2_) may contribute to sources or sinks of reactive halogens. The formation of sulfate, from the oxidation of SO_2_, is typically the largest source of acidity in the atmosphere (see Sect. 3). However, reactions of HOX with dissolved S(IV) $\left({\text{HSO}}_{3}^{-}+{\text{SO}}_{3}^{2-}\right)$ in aqueous aerosols can convert halogens to their less-reactive acid form (HX) via Reactions (R17) and (R18) (Fogelman et al., 1989; Troy and Margerum, 1991; von Glasow et al., 2002a; Chen et al., 2017; Liu and Abbatt, 2020). Here, especially the reaction with HOI can be very significant (Pechtl and von Glasow, 2007; Bräuer et al., 2013; Hoffmann et al., 2019a).

(R17) $${\text{HSO}}_{3}^{-}+\text{HOX}\to {\text{SO}}_{4}^{2-}+\text{HX}+{\text{H}}^{+}$$

(R18) $${\text{SO}}_{3}^{2-}+\text{HOX}\to {\text{SO}}_{4}^{2-}+\text{HX}.$$

The rate expression for S(VI) formation by S(IV) + HOX is given by the following:

(17) $$R_{\text{HOX},1}=k_{17,\text{HOX}}\left[{\text{HSO}}_{3}^{-}\right][\text{HOX}]$$

(18) $$R_{\text{HOX},2}=k_{18,\text{HOX}}\left[{\text{SO}}_{3}^{2-}\right][\text{HOX}],$$

with recommended rate constants for HOCl of *k*_17,HOCl_ = 2.8 × 10^5^ L mol^−1^ s^−1^ (Liu and Abbatt, 2020) and *k*_18,HOCl_ = 7.6 × 10^8^ L mol^−1^ s^−1^ (Fogelman et al., 1989) and for HOBr of *k*_17,HOBr_ = 2.6 × 10^7^ L mol^−1^ s^−1^ (Liu and Abbatt, 2020) and *k*_18,HOBr_ = 5.0 × 10^9^ L mol^−1^ s^−1^ (Troy and Margerum, 1991), respectively. Unfortunately, reaction rate constants for HOI with dissolved S(IV) $\left({\text{HSO}}_{3}^{-}+{\text{SO}}_{3}^{2-}\right)$ have not been measured yet. However, following the augmentation of Pechtl et al. (2007), the reaction rate constants of HOI with ${\text{HSO}}_{3}^{-}$ and ${\text{SO}}_{3}^{2-}$ should be even faster than the reaction rate constants of HOCl and HOBr, or it is likely diffusion limit controlled.

Finally, the overall impact of changes in anthropogenic emissions of SO_2_ or other acid gas precursors on tropospheric reactive halogen production remains unknown. Because of the impact of reactive halogens on the radiative forcing of the powerful greenhouse gas ozone (Saiz-Lopez et al., 2012a), as well as aerosol particle composition (Hoffmann et al., 2016; Lee et al., 2019), their chemistry can be of crucial importance for climate predictions. Therefore, more laboratory investigations, chamber studies, and accompanied modeling efforts are needed to determine chemical reaction rate constants of crucial halogen processes, such as the oxidation of S(IV) by HOI, and to better characterize the overall reactive cycling of halogens including its sensitivity to aerosol particle and cloud acidity.

### 4.9 Discussion and outlook: atmospheric multiphase chemistry of inorganic species

Multiple reactive pathways for the conversion of S(IV) to S(VI) have been discussed here. Many of these processes are limited in atmospheric aerosols by acidic conditions and the presence of particle-phase organics, which quench highly reactive radical and triplet species. Studies from the past 4 decades have shown that, under polluted conditions, such as those found in urban areas worldwide or in the North China Plain (NCP), only relatively stable oxidants or TMI catalysis may lead to the required rate of S(IV) for S(VI) conversion to explain the observed S(VI) budgets (Jacob and Hoffmann, 1983; Chameides, 1984; Saxena and Seigneur, 1987; Seigneur and Saxena, 1988; Pandis et al., 1992; Amels et al., 1996; Berglund and Elding, 1996). That being said, our understanding of atmospheric multiphase sulfate production, especially in the aerosol phase, is still incomplete, despite more than a century of studies on aqueous sulfur oxidation. S(IV) conversion explaining the aerosol sulfate budgets encountered today, especially under urban or semi-urban polluted conditions, still need further elucidation from the basic aqueous-phase processes to concrete field measurements. This includes the role of acidity in these processes, which could be decisive regarding whether or not a process can really be important in the environment.

Areas of focus should include the following:

- Laboratory studies of S(IV) oxidation by all pathways under atmospheric aerosol conditions, i.e., in aerosol flow tube reactors, to assess the impact of high ionic strength and other factors specific to the aerosol phase
- Advanced sulfur isotope measurements of ambient aerosol and cloud water samples to identify driving sulfur oxidation pathways under various atmospheric conditions
- Advanced knowledge of TMI-catalyzed S(IV) oxidation pathways, including the investigation of synergy effects and the role of other metal catalysts, besides Fe and Mn, present in aqueous atmospheric solutions. The impact of acidity and ionic strength on both the speciation of TMIs, i.e., their presence in free and complexed forms, and the specific chemical reaction rates of single TMIs have to be studied.
- Kinetic and mechanistic investigations on other potential oxidants, especially comparatively stable oxidants such as ROOHs and HOI
- Investigations of the pH-dependent in situ formation of key S(IV) oxidants, such as H_2_O_2_ and ROOH, resulting from TMI–HO_*x*_–DOM (dissolved organic matter) chemistry.

## 5 Interactions of acidity and chemical processes: organic systems

Acidity in aerosol particles can strongly enhance secondary organic aerosol (SOA) formation (Jang and Kamens, 2001; Jang et al., 2002, 2003; Iinuma et al., 2004; Jang et al., 2004; Liggio and Li, 2006; Surratt et al., 2007b). These early observations triggered immense research interest in investigating aqueous-phase reactions, leading to the accumulation of organic particle constituents. These so-called “accretion reactions” are often acid driven or even acid catalyzed. In the following, the most important organic compound families, and the influence of acidity on their aqueous-phase chemistry, are discussed. In this section, we discuss the role of acidity on the gas-particle partitioning of semi-volatile organic compounds through its influence on the hydration of carbonyls and dicarbonyls. We then discuss in detail the impact of acidity on the multiphase oxidation of organic material. Oxidative organic chemistry can be influenced by acidity because this influences the reactant speciation, such as in acid and diacid oxidations by radicals and non-radical oxidants, such as dissolved ozone. Finally, we discuss accretion reactions.

### 5.1 Acidity and hydration of aldehydes or ketones

Aldehydes or ketones are omnipresent in the tropospheric gas and aqueous phase, result from primary emissions, or are secondary oxidation products. The photolysis of aldehydes or ketones can be important for both their degradation in the troposphere and gas-phase oxidant production. Water-soluble aldehydes or ketones may partition into the aqueous phase of deliquesced aerosols, cloud droplets, and fog droplets. Once in the aqueous phase, these compounds can undergo hydration, leading to the conversion of the carbonyl group into gem-diol moieties. As hydration processes are typically acid or base catalyzed, the acidity of an aqueous solution can affect the hydration and, consequently, all other processes linked to it. With regard to phase partitioning, the hydration equilibria increase the effective partitioning of the carbonyl-containing compound towards the aqueous phase (Sumner et al., 2014). Moreover, compared to the carbonyl group, the diol functionality is photochemically inactive. Thus, partitioning to the aqueous phase and subsequent hydration can, in part, protect aldehydes or ketones from photolysis and shut off the possible photochemistry of the carbonyl group (George et al., 2015; Herrmann et al., 2015; McNeill and Canonica, 2016). However, hydrated aldehydes are often characterized by a somewhat lower reactivity with radical oxidants, such as OH, compared to the unhydrated carbonyl species (Schuchmann and von Sonntag, 1988).

The next subsection summarizes the present knowledge on the acidity dependence of carbonyl group hydration constants and the implications for the chemical conversions of aldehydes or ketones in atmospheric aqueous media.

#### The influence of acidity on hydration constants and its implications

**Figure 9.**
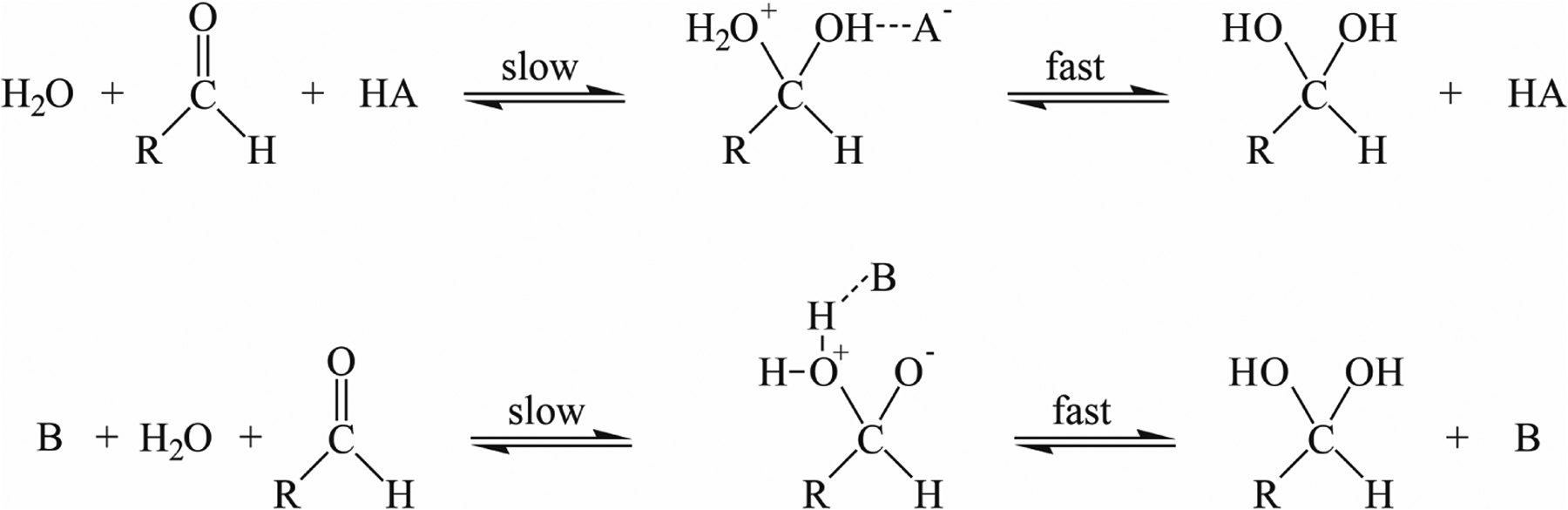
General mechanism of the acid- or base-catalyzed (A and B, respectively) formation of diols resulting from the hydration of the carbonyl group.

The reversible hydration and dehydration of the carbonyl group of an aldehyde or ketone in the aqueous phase is illustrated in Fig. 9 (Bell and Darwent, 1950; Bell, 1966; Ogata and Kawasaki, 1970; Lowry and Richardson, 1976).

Simple aldehydes, such as formaldehyde (Zavitsas et al., 1970; Li et al., 2011; Rivlin et al., 2015), acetaldehyde (Ahrens and Strehlow, 1965; Kuschel and Polarz, 2010), and glyoxal (Liggio et al., 2005a; Loeffler et al., 2006), tend to self-oligomerize, e.g., via hemiacetal formation or aldol condensation, this further influences the hydration equilibrium.

The ratio of the hydrated and dehydrated fraction under equilibrium conditions is described by the equilibrium constant *K*_hyd_ and is defined as follows for a dilute aqueous solution:

(19) $$K_{\text{hyd}}=\frac{\left[\text{diol compound}\right]}{\left[\text{carbonyl compound}\right]}=\frac{k_{\text{hyd}}}{k_{\text{dehyd}}},$$

where *k*_hyd_ is the rate constant for hydration, and *k*_dehyd_ is the rate constant for dehydration. In general, the hydration constants *K*_hyd_ decrease with decreasing the electronwithdrawing power of the substituent in a substituted aldehyde or ketone (Clayden et al., 2012). The equilibrium constants of simple aldehydes or ketones generally show no pH dependence but are dependent on temperature.

For most carbonyls, the hydration reaction with H_2_O under neutral conditions is slow. In the presence of hydrogen ions, hydroxyl ions, undissociated acid molecules, and anion bases, the hydration reaction proceeds faster. The overall hydration rate, considering all acid and base dependencies, can be calculated by means of Eq. (20) as follows (Ogata and Kawasaki, 1970; Lowry and Richardson, 1976):

(20) $$k_{\text{hyd}}=k_{0}+k_{{\text{H}}^{+}}\left[{\text{H}}^{+}\right]+k_{\text{OH}}\left[{\text{OH}}^{-}\right]+\left(k_{\text{a}}+k_{\text{b}}\frac{\left[\text{B}\right]}{\left[\text{HA}\right]}\right).$$

The catalytic constants of the hydration rate in Eq. (20) are described as follows: *k*_0_ for the solvent influence, $k_{{\text{H}}^{+}}$ for the effect of the H^+^ ion, *k*_OH_ as influence of the OH^−^ ion, and *k*_a_ and *k*_b_ as general acid or base contribution (Ogata and Kawasaki, 1970). An overview of the acid- or base-catalytic constants for the hydration of formaldehyde and acetaldehyde by a few different organic acids is presented in Table 2. As can be seen from the data compiled in Table 2, the presence of acids clearly influences the hydration rate of the carbonyl group.

Experimentally determined values for *K*_hyd_ and our recommended values are presented for several atmospherically relevant simple aldehydes, ketones, and *α*-oxocarboxylic acids in Tables S3 and S4. The general trend in *K*_hyd_ is glyoxal (second hydration) > formaldehyde > methylglyoxal (CHO group hydration) > glyoxylic acid > glyoxal (first hydration) > glyoxylate > glycolaldehyde > pyruvic acid > biacetyl > acetalydehyde > propanal > butanal > pivealdehyde > pyruvate > acetone. These data are discussed in more detail in the Supplement.

It is important to note that the hydration of simple aldehydes, ketones, and dicarbonyls is unaffected by pH. For multifunctional carbonyl compounds, the hydration equilibrium constant of the carbonyl group is strongly influenced by the electronic effects of the adjacent group. The hydration of carbonyl groups in compounds that also contain pH-sensitive moieties, such as *α*-oxocarboxylic acids, is highly influenced by the acidity of the surrounding environment.

**Figure 10.**
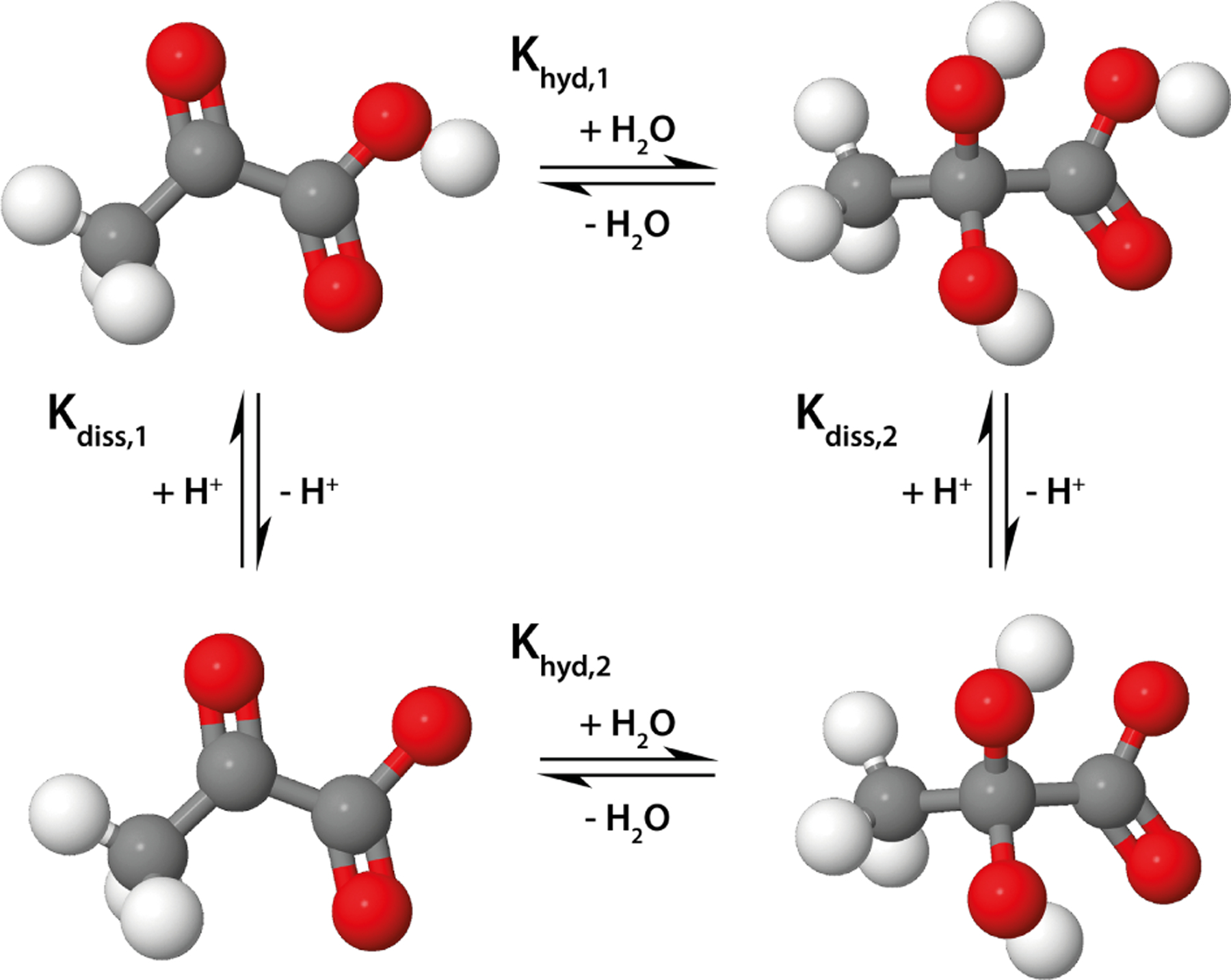
Scheme describing the four equilibria of pyruvic acid, a representative *α*-keto carboxylic acid, in aqueous solution.

Besides the hydration and dissociation equilibria, condensation (dimerization or polymerization) equilibria, as well as keto-enol equilibria, could influence these compounds (Fig. 10). The equilibria are related by *K*_hyd.1_ × *K*_diss.2_ = *K*_diss.1_ × *K*_hyd.2_, while the apparent dissociation constant is given by the following:

(21) $$K_{\text{Hyd}}=\frac{\left[{\text{H}}^{+}\right]\times K_{\text{diss},\text{1}}+K_{\text{hyd},2}\times K_{\text{diss},1}}{\left[{\text{H}}^{+}\right]+K_{\text{diss },1}}.$$

**Figure 11.**
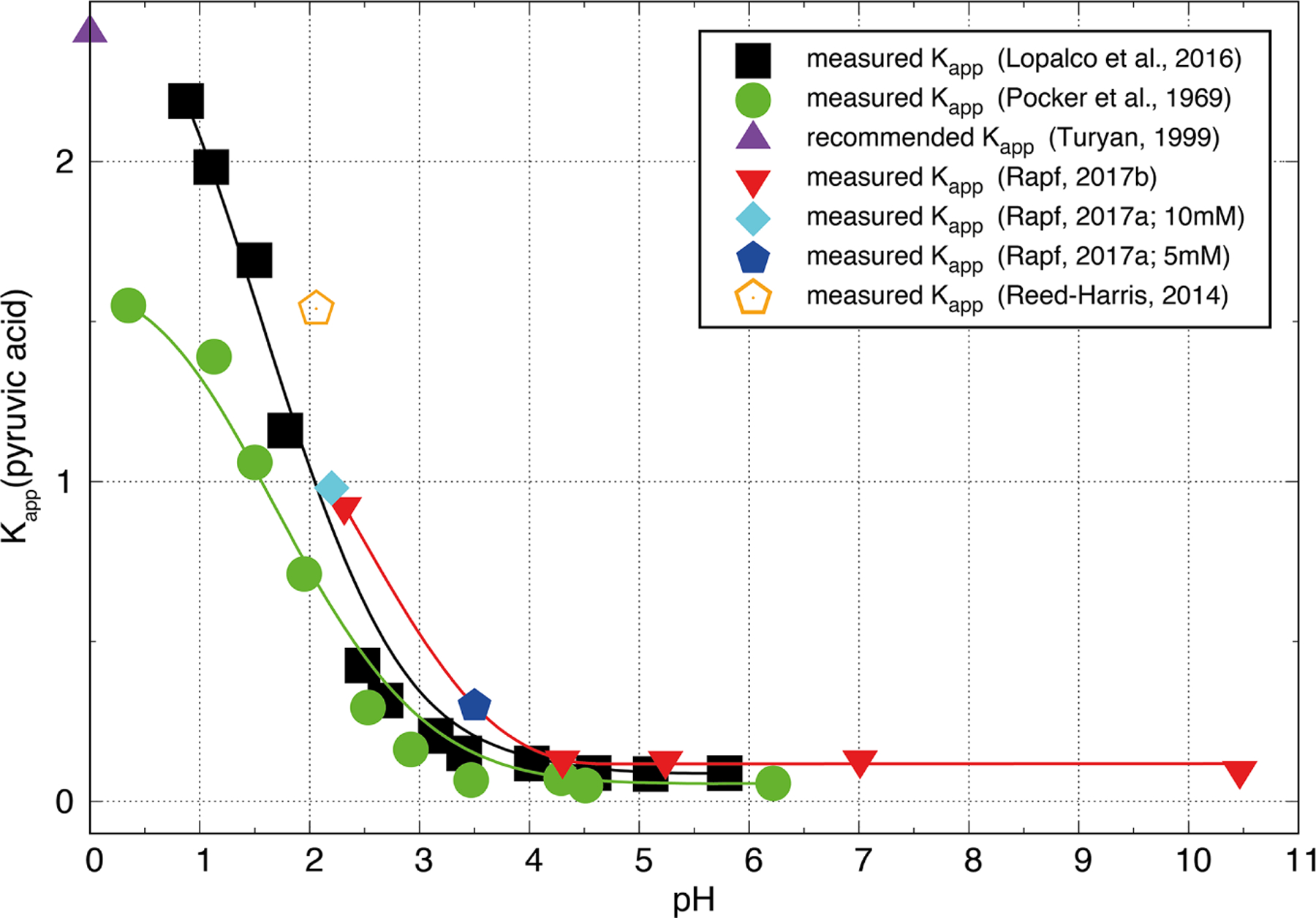
Measured pH dependency of the apparent hydration equilibria (see Eq. 21) of pyruvic acid in an aqueous solution. Experimental data are shown from the literature (Pocker et al., 1969; Reed Harris et al., 2014; Lopalco et al., 2016; Rapf et al., 2017a, b).

An overview of the determined *K*_hyd_ values for atmospherically relevant *α*-oxocarboxylic acids is given in Table S4, and the existing data are separately outlined for each of the listed chemical compounds in the Supplement. The most prominent *α*-oxocarboxylic acid compounds in the atmosphere are glyoxylic acid and pyruvic acid. Special emphasis in the recent literature was put on pyruvic acid. The recent data on pyruvic acid are summarized in Fig. 11.

As shown in Fig. 11, *K*_hyd_ for pyruvic acid increases rapidly with decreasing pH for pH < 3. Note that the formation of the hydrated pyruvic acid (2,2-dihydroxypropanoic acid) is also dependent on the water concentration (Pocker et al., 1969; Maron et al., 2011), which may have implications for aqueous aerosol chemistry.

**Figure 12.**
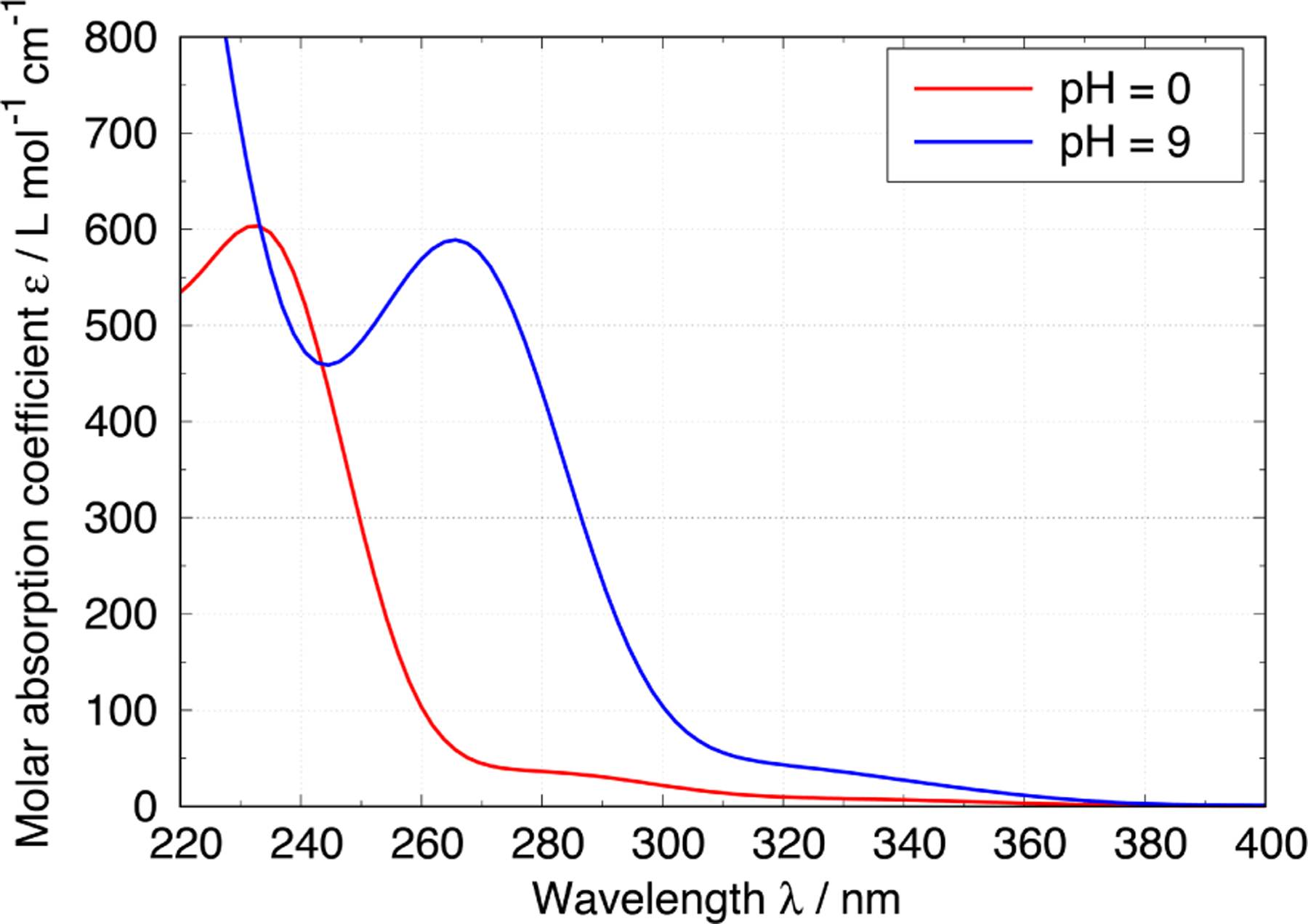
Measured UV absorption coefficient spectra of pyruvic acid in water under acidic (pH = 0; red line) and alkaline (pH = 9; blue line) conditions.

The impact of acidity and its feedback on the hydration, as well as their impact on the photochemistry of pyruvic acid, have been examined by spectroscopic investigations performed at the Leibniz Institute for Tropospheric Research (TROPOS) by Schaefer (2012). These investigations have shown that the molar absorption coefficient spectra of pyruvic acid are rather different under low- and high-acidity conditions. Measured absorption coefficient spectra of pyruvic acid at pH = 0 and pH = 9 (Fig. 12) show higher absorption coefficients under pH = 9 conditions.

At 300 nm wavelength, the measured absorption coefficient is about 4 times larger at pH = 9 than at pH = 0. Under high pH conditions (pH = 9), a large fraction of pyruvic acid is present in its unhydrated form, and, consequently, higher absorption coefficients are observed, compared to very acidic conditions, where pyruvic acid is mainly present in its photochemically inactive hydrated form. This difference has implications for the photochemistry of pyruvic acid, which will become less efficient in more acidic solutions compared to less acidic ones. Such effects should be implemented into aerosol liquid water chemistry models. Finally, hydration processes can be characterized by both temperature and acidity dependencies, particularly for *α*- and *β*-keto carboxylic acids such as pyruvic acid. These dependencies need to be included in future models to be able to accurately investigate their impact on the partitioning and the multiphase chemistry. For that reason, more laboratory studies on pH-dependent hydrations are needed to extend the available database for other atmospherically relevant functionalized *α*- and *β*-keto carboxylic acids. In concrete terms, more studies appear desirable for glyoxylic acid, mesoxalic acid, oxalacetic acid, and oxalglycolic acid.

### 5.2 pH-sensitive organic accretion reactions

Organic accretion reactions are considered to be a source of high molecular weight organic material in atmospheric aerosols, which plays a key role in the formation of secondary organic aerosol material (Barsanti and Pankow, 2004, 2005, 2006). These reactions are typically multistep, bondforming reactions and are highly pH sensitive. Many organic accretion reactions are acid or base driven or, in some cases, even acid catalyzed. In these acid-driven reactions, the protons (H^+^) in the reactions are incorporated into the reaction products formed (e.g., the ring opening of epoxides; cf. Sect. 5.3) and are therefore not “acid catalyzed”. Examples of atmospherically important accretion reactions include (i) aldol condensation, (ii) hemiacetal and acetal formation, and (iii) esterification of carboxylic acids, which will be treated in the following subsections.

The current kinetic and mechanistic knowledge on tropospheric accretion reactions has recently been summarized in a review by Herrmann et al. (2015) and a book by Barker et al. (2016). Accordingly, the present subsection only briefly outlines the mechanisms and emphasizes their dependence on acidity. For further specific details on organic accretion reactions and other linked important pH-dependent reactions of organic compounds, such as hydrolysis reactions, please see Larson and Weber (1994) Herrmann et al. (2015), Zhao et al. (2016), Ng et al. (2017), and Brüggemann et al. (2020) and references therein.

#### 5.2.1 Aldol condensation and ammonium catalyzed reactions

##### Overview

The aldol (short form of “aldehyde alcohol”) condensation is a carbon–carbon bond formation requiring the participation of an enolizable carbonyl compound (e.g., ketones or aldehydes with an *α* hydrogen; Loudon, 1995). Under acidic conditions, the enol reacts with a protonated carbonyl compound to form the aldol addition product. The product may be dehydrated to form the aldol condensation product, a conjugated enone compound. The acid- or base-catalyzed nature of the enol formation, as well as the role of the protonated carbonyl reactant under acidic conditions, makes this family of reactions pH sensitive. This pathway has been suggested as being a source of light-absorbing secondary organic material (i.e., brown carbon) in atmospheric aerosols (Laskin et al., 2015; Nozière et al., 2015), which was discussed in more detail for acetaldehyde, glyoxal, and methylglyoxal (Noziere and Esteve, 2005; Nozière et al., 2007; Noziere and Esteve, 2007; Noziere and Cordova, 2008; De Haan et al., 2009a; Shapiro et al., 2009; Bones et al., 2010; Sareen et al., 2010; Li et al., 2011; Yu et al., 2011; Kampf et al., 2012; Nguyen et al., 2012; Laskin et al., 2015; Lin et al., 2015; Maxut et al., 2015; Nozière et al., 2015; Van Wyngarden et al., 2015; Aiona et al., 2017; Rodriguez et al., 2017). Several studies which focused on sulfuric-acid-catalyzed aldol formation have shown that this chemistry only occurs efficiently under strongly acidic conditions with pH < 2 (Duncan et al., 1999; Kane et al., 1999; Imamura and Akiyoshi, 2000; Nozière and Riemer, 2003; Esteve and Noziere, 2005; Noziere and Esteve, 2005; Liggio and Li, 2006; Noziere et al., 2006; Casale et al., 2007; Noziere and Esteve, 2007; Krizner et al., 2009).

##### Surface films

Van Wyngarden et al. (2015) reported on the formation of surface films from H_2_SO_4_ propanal mixtures with or without glyoxal and/or methylglyoxal. These films tended to form faster when the acidity was increased up to 48 wt % H_2_SO_4_, but, with an acidity of 76 wt % H_2_SO_4_, the film formation slowed down or even stopped in all mixtures except propanal/glyoxal ones.

##### Mechanistic and kinetic considerations

Yasmeen et al. (2010) suggested that the favorable mechanism under acidic conditions (pH < 3.5) is the acetal formation, while the aldol condensation only occurs at a pH = 4–5, which is in contrast to the abovementioned conditions. Ammonium-catalyzed or amine-catalyzed aldol condensation proceeds under higher, but still acidic, pH values typical for tropospheric aerosol particles (Noziere and Cordova, 2008; De Haan et al., 2009a; Noziere et al., 2010; Sareen et al., 2010; Li et al., 2011; Sedehi et al., 2013; Powelson et al., 2014; Aiona et al., 2017). The pH-dependent rate constants for aldol condensation reactions of glyoxal and methylglyoxal with ammonium sulfate and amines have been further investigated in several studies (Noziere et al., 2009; Sareen et al., 2010; Yu et al., 2011; Kampf et al., 2012; Sedehi et al., 2013; Powelson et al., 2014; Yi et al., 2018). Noziere et al. (2009) observed that the pathway via the iminium ion is faster at a higher pH, whereas the aldol condensation is favored at lower pH values, which suggests a pH dependency incorporation of N-containing products. The results of the pH dependency appear to be contradictory. On the one hand, Sareen et al. (2010) reported an enhanced product formation by decreasing the pH and concluded that there was an acid-catalyzed aldol formation of the light-absorbing product at 280 nm. On the other hand, Yu et al. (2011) reported an exponential increase in the formation rate of condensation products, e.g., imidazole, by increasing the pH and concluded that there was an ammonium-catalyzed reaction. Similarly, Kampf et al. (2012) observed a higher production rate of the imidazole bicycle with increasing pH values. Furthermore, Sedehi et al. (2013) showed a strong pH dependence with an increasing reaction rate proportional to the concentration of the deprotonated amine or, in other words, an increase in the pH value. Nevertheless, the pH-dependent character of the reaction of ammonium sulfate or amine reaction with glyoxal is stronger than for methylglyoxal. A study by Yi et al. (2018) describes an acceleration of the pH-dependent reaction of ammonium sulfate or amine in the presence of glycolaldehyde, whereas no cyclic compounds (e.g., imidazole) were formed in this reaction. Powelson et al. (2014), Grace et al. (2019), and Li et al. (2019) reported the formation of heterocyclic compounds under similar conditions. Hawkins et al. (2018) reported an increasing formation of pyrazine-based chromophores in an aqueous mixture containing methylglyoxal and ammonium sulfate by increasing the pH from 2 to 9. This indicates that nitrogen, as a nucleophile, is more important than the acid-catalyzed aldol condensation, which is consistent with the observation of Kampf et al. (2012, 2016) and Yi et al. (2018). A theoretical analysis of glyoxal condensation in the presence of ammonia conducted by Tuguldurova et al. (2019) describes different imidazole formation pathways by the formation of key intermediates, namely ethanediimine, diaminoethanediol, and aminoethanetriol, required for the imidazole ring cyclization. These authors reported that the imine concentrations are very low due to the high-energy barriers for imine formation. Although a pH decrease due to amino alcohol dehydration leads to higher imine concentrations, but also to higher ammonium cation formation, which hinders the ammonium addition to the carbonyl group, due to the electrostatic interactions. Hence, the reaction pathway via the ethandiimine as intermediate is not the main reaction pathway. The second proposed pathway of glyoxal condensation in the presence of ammonia, which includes the formation of the intermediate aminoethanetriol, has a lower energy and is apparently kinetically more favorable due to the higher concentration of this intermediate. Finally, imidazole formation is determined by the glyoxal concentration, the ratio of glyoxal / (amine or ammonium), the composition of the solvent, and the pH value.

#### 5.2.2 Hemiacetal and acetal formation

Hemiacetal and acetal formation are the addition of an organic molecule containing either one or two hydroxyl groups (e.g., alcohols) to a carbonyl compound, leading to the formation of one (hemiacetal) or two (acetal) ether-type C−O−C bonds. This type of accretion reaction is significant for aqueous secondary organic aerosol formation involving glyoxal, methylglyoxal, acetaldehyde, formaldehyde, and other common atmospheric carbonyl-containing compounds (Schweitzer et al., 1998; Tobias and Ziemann, 2000; Jang et al., 2002; Kalberer et al., 2004; Hastings et al., 2005; Liggio et al., 2005b, a; Loeffler et al., 2006; Zhao et al., 2006; De Haan et al., 2009a, b; Shapiro et al., 2009; Sareen et al., 2010; Yasmeen et al., 2010; Li et al., 2011). Hemiacetal formation is initiated by the protonation of a carbonyl group, followed by the nucleophilic addition of the alcohol (Loudon, 1995). After the deprotonation of the attacking alcohol, the hemiacetal is formed. Promoted by acidity, the hemiacetal can react further to a full acetal, by the protonation of the alcohol group of the hemiacetal, to eliminate water again under the formation of a carbocation. This carbocation can react in a subsequent reaction with another alcohol molecule to form the full acetal by the deprotonation of the hydroxyl group. Hemiacetal and acetal formation are reversible. In addition to the aldol condensation product, Liggio et al. (2005a, b) and Liggio and Li (2006) reported on an acetal formation during the reactive uptake of glyoxal and pinonaldehyde on acidic aerosols. It has been reported by De Haan et al. (2009b) that glyoxal is more prone to undergoing the acetal formation, while methylglyoxal reacts mainly by the aldol condensation reaction mentioned above, whereas the contribution of oligomer formation was strongly dependent on the relative humidity and, hence, the particulate water concentration. Holmes and Petrucci (2006, 2007) observed the formation of hemiacetals in the oligomerization process of levoglucosan induced by Fenton chemistry. Noziere et al. (2009, 2010) observe a pH-dependent ammonium-catalyzed acetal formation from glyoxal and acetaldehyde. The hydration and the subsequent acetal formation involving methylglyoxal is strongly dependent on the pH value and occurs at pH < 3.5 (Yasmeen et al., 2010). Maxut et al. (2015) also investigated the ammonium-catalyzed imidazole formation, with glyoxal in neutral aqueous solution, and concluded that the contribution of acetal oligomer formation pathway is small. Grace et al. (2019) referred to the studies by De Haan et al. (2011) and Kampf et al. (2016), who reported that aldol condensation-type reactions are more important than acetal or hemiacetal formation under atmospheric conditions. In summary, hemiacetal and acetal formation require acidic conditions, but the contribution of this reaction pathway is small compared to aldol formation under atmospheric conditions.

#### 5.2.3 Esterification of carboxylic acids

Esterification is a reversible, acid- or base-catalyzed condensation reaction of carboxylic acids and hydroxyl group containing molecules under the formation of an C(O)−O−C-type bond (Ingold, 1969; Larson and Weber, 1994). The acid-catalyzed mechanism can be described as follows: the carbonyl group of the undissociated carboxylic acid can be protonated under acidic conditions to form a carbocation. The carbocation then is subject to a nucleophilic attack by a hydroxyl-group-containing molecule. The resulting intermediate further reacts by tautomerization (proton shift in the molecule), which subsequently decays in an equilibrium reaction to a protonated ester and a water molecule (Loudon, 1995).

The base-catalyzed mechanism includes the reaction of a carboxylate group (resulting from the deprotonation of carboxylic acid group) and a hydroxyl-group-containing molecule, such as an alcohol. First, a proton transfer from the alcohol to the carboxylate occurs. Second, the deprotonated hydroxyl group reacts in a nucleophilic attack with the carbon atom of the carboxylic acid, forming a metastable intermediate, which subsequently decays to an ester molecule and a hydroxide ion in an equilibrium reaction. In addition to being pH sensitive, esterification reactions are also strongly dependent on the water content. The majority of esters are hydrolyzed in the presence of water. Both the formation and the hydrolysis of esters are slow processes under tropospheric conditions (see Herrmann et al., 2015, for further details). Moreover, the hydrolysis rate of esters will increase with increasing acidity (Mabey and Mill, 1978; Herrmann et al., 2015). Altieri et al. (2006, 2008) reported that the esterification mechanism occurs in the cloud processing of pyruvic acid (pH = 2.7–3.1) and methylglyoxal (pH = 4.2–4.5). The oxidation of organic compounds leads, in general, to carboxylic acids and proceeds through *α*- or *β*-hydroxy acid to esters or oligoesters, similarly to the proposed mechanisms for oligomers in the aerosol phase (Gao et al., 2004; Tolocka et al., 2004; Surratt et al., 2006b, 2007a). Since then, ester formation by the oxidation of organic matter in the troposphere has been the subject of many laboratory investigations (Hamilton et al., 2006; Surratt et al., 2006a; Szmigielski et al., 2007; Altieri et al., 2008; Galloway et al., 2009; Zhang et al., 2011; Birdsall et al., 2013; Kristensen et al., 2013; Strollo and Ziemann, 2013; Claflin and Ziemann, 2019; Mekic et al., 2019) and field studies (Raja et al., 2008, 2009; Kristensen et al., 2013). The work from Birdsall et al. (2013) suggested that esterification by the condensation of carboxylic acids with hydroxyl-group-containing molecules is not efficient enough to explain the oligoesters under realistic aerosol acidities. In a recent study by Zhao et al. (2019), heterogeneous oxidation processes near the gas particle interface open up a further formation pathway of ester-like structures, namely that the dimerization of organic-oxygen-containing radicals dominantly leads to ester formation. In summary, it should be noted that this accretion reaction in the atmospheric aerosol-phase depends more on the hygroscopicity than on the acidity of the aerosols (Zhao et al., 2006; De Haan et al., 2009a), since the hydrolysis competes with the ester formation.

### 5.3 Epoxide reactions

#### 5.3.1 Isoprene-derived epoxides

**Figure 13.**
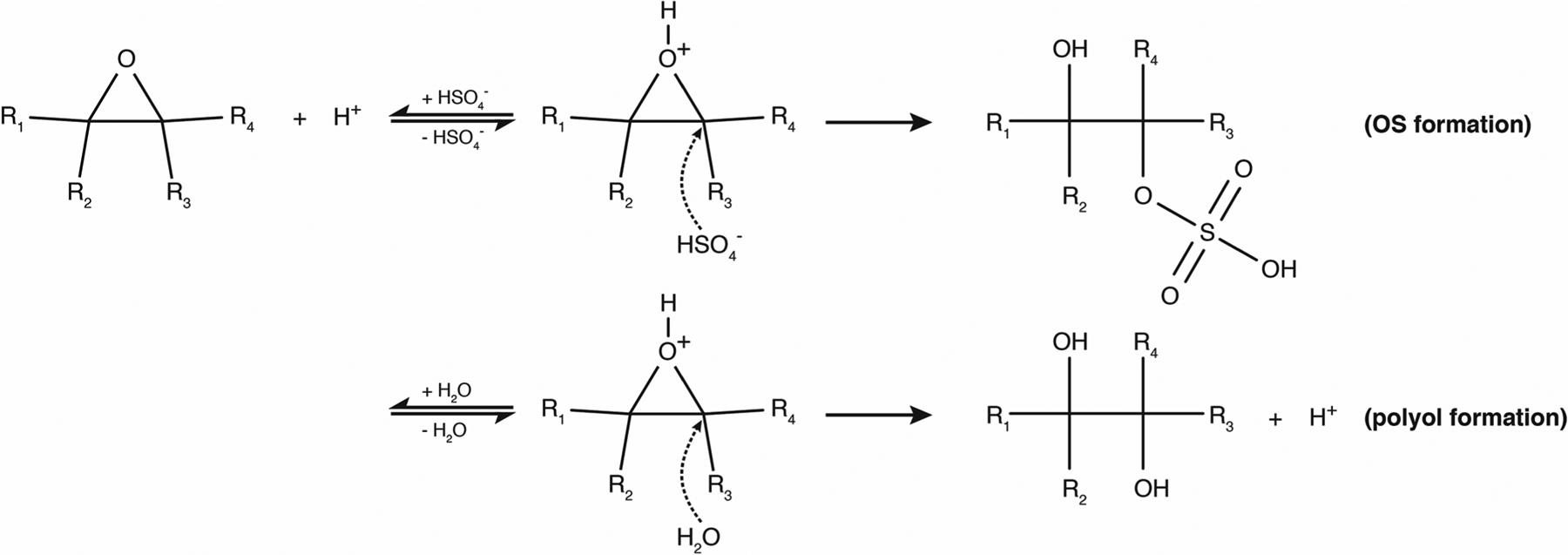
Schematic of the OS and polyol formation via the acid-catalyzed ring-opening reactions of epoxides.

In the last decade, acid-catalyzed ring-opening reactions of epoxides (see Fig. 13) in aqueous aerosols have emerged as a significant source of secondary organic aerosol material. In the aqueous phase, the protonation of the epoxide by an acid (H^+^ or ${\text{NH}}_{4}^{+}$; Minerath et al., 2008; Minerath and Elrod, 2009; Eddingsaas et al., 2010; Nguyen et al., 2014; Noziere et al., 2018) occurs in concert with nucleophilic addition. Typically, the participating nucleophiles are H_2_O, ${\text{HSO}}_{4}^{-}$, and ${\text{SO}}_{4}^{2-}$, although amines (Stropoli and Elrod, 2015) and alcohols (Surratt et al., 2010; Piletic et al., 2013) can also contribute. A nucleophilic attack by H_2_O results in hydrolysis and polyol formation, thus explaining the presence of isoprene-derived tetrols in particles (Kourtchev et al., 2005; Xia and Hopke, 2006; Liang et al., 2012; Zhang et al., 2013). The hydrolysis of epoxides catalyzed by ${\text{NH}}_{4}^{+}$ can only be important in less acidic aerosol solutions due to the orders of magnitude of lower-rate coefficients (Noziere et al., 2018). The addition of ${\text{HSO}}_{4}^{-}$ or ${\text{SO}}_{4}^{2-}$ to the protonated epoxide in the aerosol phase is a more efficient pathway for organosulfate (OS) formation than radical mechanisms (McNeill et al., 2012; Schindelka et al., 2013). While the formation of polyols via the hydrolysis of epoxides may be acid catalyzed (Eddingsaas et al., 2010), OS formation can consume H^+^ (Riva et al., 2019; Brüggemann et al., 2020; e.g., see Fig. 13).

Isoprene epoxydiol (IEPOX), a photooxidation product of isoprene (Paulot et al., 2009; Surratt et al., 2010), is calculated to contribute 34 % to the global SOA mass (Lin et al., 2012) and 28 % the organic aerosol mass in southeastern USA (Marais et al., 2016). The reactive uptake of IEPOX to aqueous media is strongly pH dependent, with the reactive uptake coefficient decreasing rapidly with increasing pH for pH > 1 (Gaston et al., 2014). For this reason, the rate of IEPOX SOA formation is slow in cloud water (McNeill, 2015), but, given the relatively large liquid water content of clouds and the relatively large water solubility of IEPOX, it could be significant in more acidic cloud droplets (pH 3–4; Tsui et al., 2019).

#### 5.3.2 Terpene-derived epoxides

In regions with lower isoprene but higher monoterpene emissions, e.g., in the boreal forests, monoterpene-derived OSs formed via different proposed pathways can also contribute to SOA mass in atmospheric aerosols (see Brüggemann et al., 2020, for details). Their importance for SOA is still not well characterized. Formation of monoterpene-derived OS has been observed in chamber experiments and measured in field samples (Iinuma et al., 2004, 2007; J. Ye et al., 2018; Brüggemann et al., 2019; Cui et al., 2019). However, there are only a few measurements of monoterpene-derived OSs in boreal forest areas.

OS formation via acid-catalyzed ring-opening reactions of several monoterpene epoxides (*β*-pinene oxide, limonene oxide, and limonene dioxide) has been kinetically and mechanistically investigated (Cortes and Elrod, 2017). Investigations demonstrated that monoterpene epoxides react faster than IEPOX in an aqueous solution and might even react in less acidic solutions. However, this study also revealed that the formed OS compounds are not long-lived compounds under aqueous aerosol conditions and may quickly react further – mainly through hydrolysis. Therefore, Cortes and Elrod (2017) concluded that OS-formation mechanisms, other than the acid-catalyzed ring-opening mechanism of monoterpene epoxides, are needed to explain the formation of more long-lived OS from monoterpenes. In agreement with these findings, recent chamber studies on the OS formation from *α*-pinene oxidation (Duporte et al., 2016, 2020) showed that the OS yield, including the subsequent formation of OS dimers and trimers, decreases with increasing relative humidity. Furthermore, these studies revealed that effective formation rates of OS from *α*-pinene are 2 orders of magnitude higher under very acidic aerosol conditions, and that the OS formation under slightly acidic aerosols conditions is limited. Further sensitivity studies showed a strong dependency of the OS formation on the available sulfate, supporting an acid-catalyzed processing of monoterpene epoxides yielding OS. However, it should be noted that regions with high monoterpene emissions are usually not associated with high sulfate aerosol loadings and quite acidic aerosols; hence, their contribution to SOA might be limited and only important in mixed environments.

#### 5.3.3 Other epoxides

Other atmospheric epoxides have been proposed to contribute to SOA formation, including 2-methyl-3-buten-2ol (MBO; Mael et al., 2015), methacrylic acid epoxide (MAE; Lin et al., 2013; Birdsall et al., 2014), and epoxides from toluene oxidation (Baltaretu et al., 2009; McNeill et al., 2012). However, none of these species have the relatively large gas-phase production rate and water solubility of IEPOX (see Brüggemann et al., 2020, and references therein for details), so they probably lead to small SOA mass contributions.

### 5.4 Oxidation reactions of acids and diacids

Acidity changes the speciation of dissociating organic compounds in the atmospheric aqueous phase. More specifically, acidity decreases the degree of dissociation for organic acids, i.e., lowers the fraction of a compound in its deprotonated form. The protonated and deprotonated forms of a dissociating compound are characterized by different molecular properties (e.g., different bond dissociation energies, BDEs). Therefore, key aqueous-phase oxidants, such as the radicals OH and NO_3_ or the non-radical oxidant O_3_, may react via different possible reaction pathways and kinetics with the protonated and deprotonated forms. Accordingly, acidity can strongly affect the chemical processing of dissociating organic compounds.

Within the next subsection, the potential effect of acidity on the chemical processing of dissociating organic compounds in atmospheric aqueous solutions is summarized. The discussion will focus primarily on acids and their respective anions; however, acidity may influence reactivity and partitioning for any dissociating species (including, e.g., imidazoles or phenols).

#### 5.4.1 Reaction pathways of dissociating organic compounds with different oxidants

As in the gas phase, radical oxidants, such as OH and NO_3_, can react with dissociating organic compounds via H abstraction. The oxidation of dissociated organic compounds may also proceed through an electron transfer reaction (ETR). For unsaturated organic compounds, radical addition to the C=C double bond represents a third possible reaction pathway. Overviews on atmospheric aqueous-phase radical oxidants are available in Buxton et al. (1988), Herrmann (2003), and Herrmann et al. (2010, 2015).

**Figure 14.**
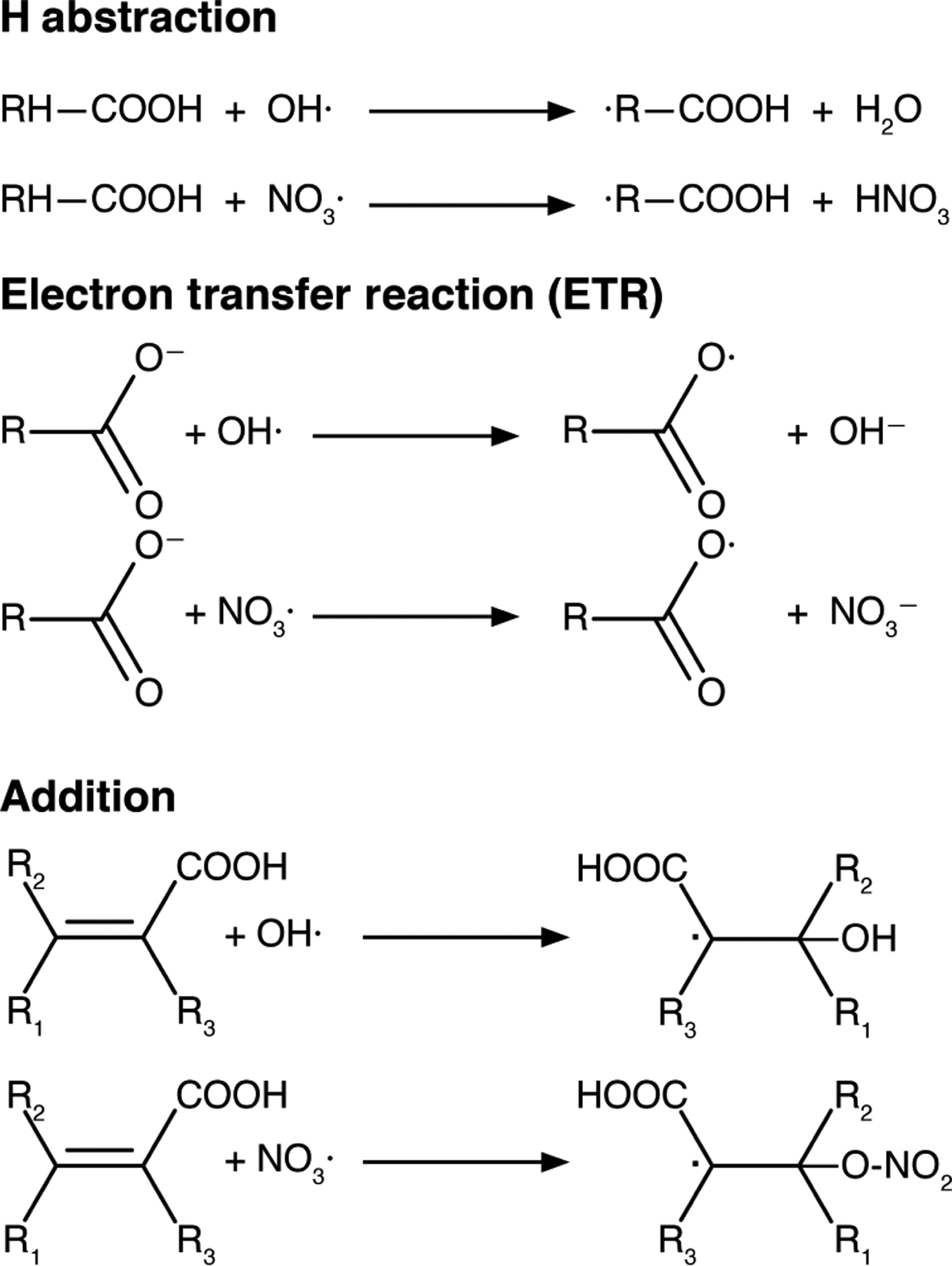
Schematic of the initial reaction steps for the most important radical oxidation pathways of dissociating organic compounds (exemplified for carboxylic acids).

In Fig. 14, the three types of radical-initiated reactions are schematically displayed for carboxylic acids, the most prominent dissociating organic compound class in tropospheric aerosols, and clouds. The H-abstraction-related reactivity of an organic molecule strongly depends on the BDE of the abstractable hydrogen atoms. Carbon–hydrogen bonds (C–H) are typically characterized by lower BDEs (e.g., BDE = 410 ± 5 kJ mol^−1^ for acetone; Herrmann, 2003) than other bonds, such as oxygen–hydrogen bonds (e.g., O–H in acids; BDE = 445 kJ mol^−1^; Luo, 2002). However, please note that the given BDEs are gas-phase BDEs, and that BDEs can be slightly altered by an aqueous solvent. As a consequence of the weaker carbon–hydrogen bonds, the H-abstraction reaction is currently expected to predominantly proceed at the carbon chain of dissociating organic compounds and not on the hydroxyl group, e.g., of the acid group. The H abstraction leads to carbon-centered radicals that further react with oxygen, leading to the formation of peroxy radicals and, subsequently, to functionalized organic compounds with possibly changed dissociation properties. Dissociated organic compounds can also react with radical oxidants via ETR in the aqueous phase, e.g., by removing an electron from a deprotonated and ionized acid group. Particularly for more selective radical oxidants, such as NO_3_ (or others such as ${\text{Cl}}_{2}^{-}$ and ${\text{Br}}_{2}^{-}$), ETR is often preferred over H abstraction. The reaction rate constants of NO_3_ for ETRs are generally larger than those for H abstraction. Overall, the different contributions of ETR and H-abstraction pathways also modify the product distributions as a function of pH. On one hand, the aqueous-phase H-abstraction reactions lead mostly to a functionalization of the acid (see, e.g., Leitner and Doré, 1997; Tan et al., 2012; Otto et al., 2017). On the other hand, ETR reactions of dissociated acids lead to a decarboxylation of the acid (Exner et al., 1994; Chawla and Fessenden, 2002), resulting in a formation of CO_2_ and a smaller carbon chain compound.

The third mentioned pathway of radical oxidants, i.e., the addition reaction, occurs for unsaturated aliphatic and aromatic compounds. This reaction type is typically the fastest radical reaction pathway and proceeds almost at the aqueous-phase diffusion limit (see Sect. 5.4.2), except for double bonds, where the electron density is strongly reduced by electron-drawing substituents, such as halogen atoms.

Besides the radical oxidation reactions, dissociating organic compounds can also be oxidized by ozone. In aqueous solutions, the decomposition of ozone is strongly affected by the acidity, due to its strong chemical interaction with the water matrix (see Herrmann et al., 2015, and references therein). Ozone is known to be an electrophilic and selective oxidant for organic compounds, with particular selectivity for C=C double bonds. Therefore, ozone reacts primarily with both unsaturated aliphatic compounds and aromatic compounds. O_3_ is also known to react slowly with saturated aliphatic compounds, such as hydrated organic acids and carbonyl compounds. Rate constants for these reactions have recently been compiled in Herrmann et al. (2015).

Similar to radical oxidants, ozone reactions are expected to proceed via (i) H abstraction (e.g., from the hydrated carbonyl groups of carboxylic acids; see Schöne and Herrmann, 2014), (ii) addition onto C=C double bonds (e.g., in case of unsaturated aliphatic compounds and aromatic compounds; see Mvula and von Sonntag, 2003, and Leitzke and von Sonntag, 2009), and, finally, (iii) ETR (see Mvula and von Sonntag, 2003). However, current knowledge of the abovementioned ozone oxidation mechanisms remains quite limited. An overview of the proposed oxidation mechanisms of aqueous-phase ozone can be found, e.g., in Hoigne and Bader (1983a, b), Mvula and von Sonntag (2003), Leitzke and von Sonntag (2009), von Sonntag and von Gunten (2012), and Schöne and Herrmann (2014) and references therein.

#### 5.4.2 Comparison of kinetic data of dissociated and undissociated organic compounds

To examine the effect of acidity on the chemical processing of dissociating organic compounds, kinetic data for their oxidation by OH, NO_3_, and O_3_ have been newly compiled for the present review, following several published review articles and data compilations (Buxton et al., 1988; Neta et al., 1988; Ross et al., 1998; Herrmann, 2003; Herrmann et al., 2010, 2015; Bräuer et al., 2019). These data are presented in Tables S5–S7 and Figs. S3–S6 in the Supplement. It should be noted that the tables and figures in the Supplement only show kinetic data for dissociating organic compounds where data for both their protonated and deprotonated form are available. For comparing differences in the kinetic reactivity data of protonated and deprotonated organic compounds, a reactivity ratio *κ*_R_ has been calculated. The calculated ratios are defined as the quotient of the kinetic reaction rate constants of deprotonated and protonated form (see below) with the respective oxidant (OH, NO_3_, and O_3_) as follows:

(22) $$\kappa_{\text{R}}(i)=\frac{k_{\text{deprotonated}}^{298\text{ K}}}{k_{\text{protonated}}^{298\text{ K}}}\;\left(i=\text{OH},{\text{NO}}_{3},{\text{O}}_{3}\right).$$

In brief, values of *κ*_R_(*i*) above 1 imply that the reaction of the deprotonated form will proceed more readily than the reaction of the protonated acid. Furthermore, in case of an acid, a *κ*_R_(*i*) above 1 implies that, at higher pH, with an increased abundance of the deprotonated form, the overall reaction rate of a compound will be increased, i.e., oxidations are favored under decreasing acidic conditions (cf. Sect. 5.4.3).

##### OH radical oxidations

**Figure 15.**
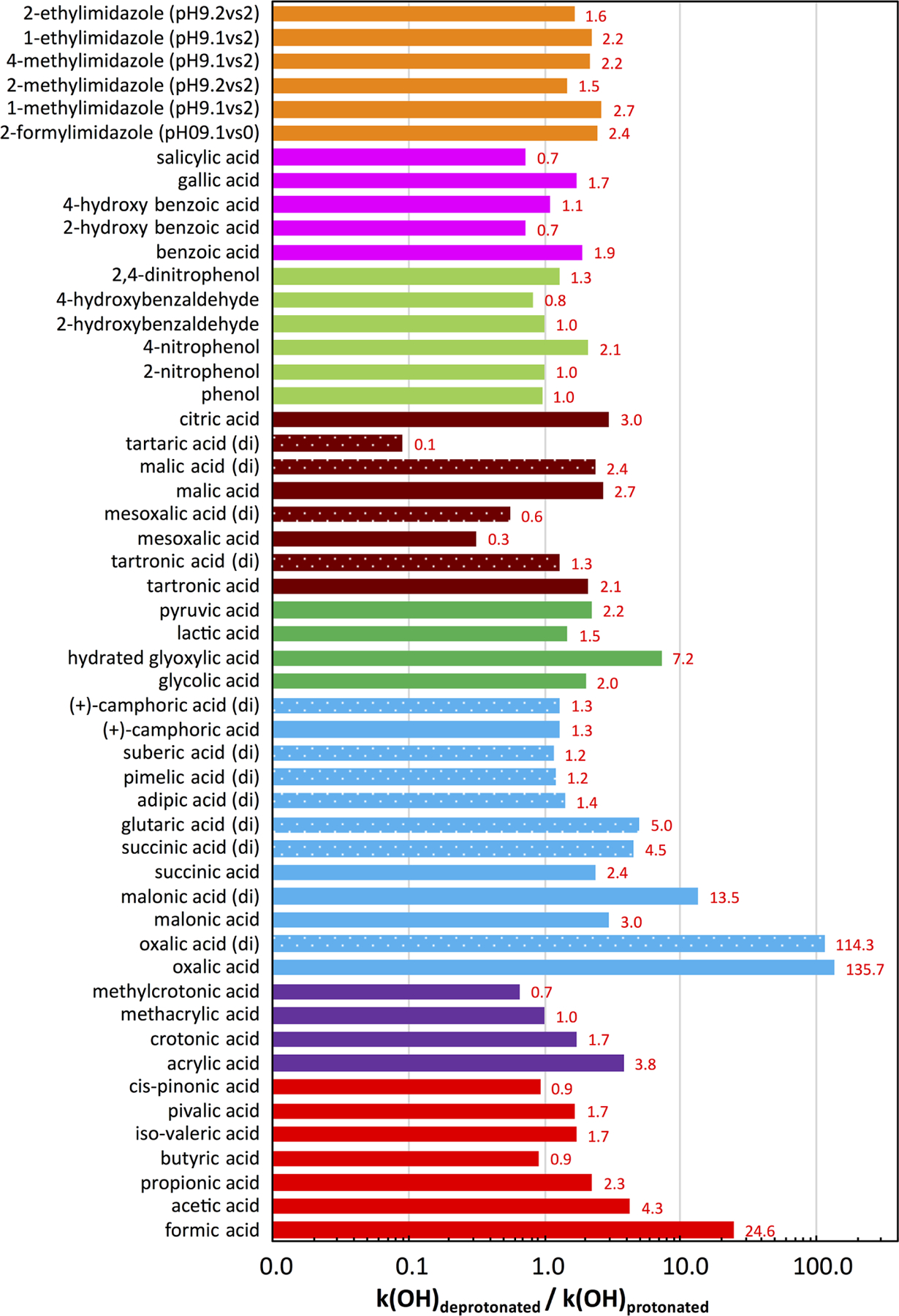
Calculated reactivity ratios *κ*_R_(OH) of different dissociating organic compounds. The *κ*_R_(OH) ratio of the dianion and protonated diacid is indicated by “(di)”. The applied aqueous-phase OH reaction rate constants are provided in Table S5 in the Supplement. Different colors indicate different compound classes, such as unsubstituted saturated monoacids (red), unsubstituted unsaturated monoacids (purple), unsubstituted saturated diacids (blue), substituted saturated monoacids (green), substituted saturated diacids (brown), phenols (light green), aromatic acids (pink), and imidazoles (orange). The dotted bars mark the ratio of the dianions.

Overall, for OH reactions, the impact of acidity on the chemical kinetics is often quite small and only crucial for some specific compounds. Thus, with respect to OH reactions, acidity will mostly alter the lifetime of dissociating compounds mainly because of its impact on the partitioning and, consequently, the affected aqueous-phase concentrations and not so much because of changes in the OH kinetics (see also Sect. 5.4.3). Figure 15 shows the calculated ratios (*κ*_R_(OH)) of OH reactions with several dissociating organic compounds. The calculated reactivity ratios are typically close to unity, i.e., a similar reactivity exists for the undissociated molecule and its corresponding anion. Larger ratios are calculated for a few small carboxylic and dicarboxylic acids. For formic acid and malonic acid, the *κ*_R_(OH) ratios are larger than 10, and for oxalic acid, they are larger than 100. For mono- and dicarboxylic acids, Fig. 15 shows decreasing ratios with an increasing carbon chain. For larger carboxylic acids, calculated ratios are scattered around unity. This result indicates that the reaction mechanism of the carboxylic acid and the corresponding carboxylate is similar for the larger acids, and H abstraction is the dominant reaction pathway. With a longer carbon chain, the impact of the acid functionality decreases, and thus, almost no acidity dependence exists. This also seems to be true for functionalized carboxylic acids. On the other hand, the substantially larger ratios of the smaller carboxylic acids demonstrate a much faster OH degradation of the smaller carboxylates compared to their protonated acids. This implies that the carboxylate group and their steric effects on the surrounding C–H bonds can facilitate an easier H abstraction. This leads to higher H-abstraction rate constants for smaller carboxylates compared to corresponding protonated acids. Besides, former studies have shown that the ETR pathway contributes little, and Schuchmann et al. (1995) reported a contribution of less than 5 %. Thus, the ETR pathway should not be responsible for the reactivity difference.

Acetic acid and acetate show the lowest OH reactivities among the considered unsubstituted monocarboxylic acids, with 1.70 × 10^7^ and 7.30 × 10^7^ L mol^−1^ s^−1^ (Chin and Wine, 1994), respectively. The weakest bond H atoms in these molecules are part of the methyl group. Those primary C–H bonds are much stronger than secondary or ternary C–H bonds. This explains why acetic acid is less reactive towards OH compared to higher non-substituted primary C–H bonds than monocarboxylic acids containing more CH_2_ groups or secondary C–H bonds. This also explains why the reactivity difference of acetic acid and acetate is more distinct, and why the impact of the carboxylate group is higher compared to longer chain acids.

Moreover, it is worth mentioning that the comparison of the kinetic differences of protonated and deprotonated carboxylic acids, given in Fig. 15, is more comprehensive compared to the work of Zhao et al. (2016). In Zhao et al. (2016), the OH oxidation kinetics of formic, glyoxylic, pyruvic acid, lactic acid, malic acid, and oxalic acid have been compared. Based on the limited kinetic data and, particularly, because of the selected compounds, this study concluded that the oxidation of carboxylate forms is much more rapid compared to that of free carboxylic acid, indicating an acidity dependence in the reactivity of carboxylic acids. However, from the present study, it can be concluded that this not true, except for smaller organic acids that are characterized by higher *κ*_R_(OH) ratios, due to their special structure properties, such as less or even no abstractable carbon-bonded H atoms as in case of oxalic acid. Hence, an acidity dependence in the reactivity exists only for these smaller carboxylic acids, and almost no acidity dependence exists for other carboxylic acids with a longer carbon chain. Therefore, the statements of Zhao et al. (2016), regarding the pH dependence in the reactivity of saturated carboxylic acids, are by far too overgeneralized. For the sake of completeness, a more recent study by the same authors (Amorim et al., 2020) shows that larger organic acids indeed do not exhibit much pH dependence.

The rate constants for unsaturated aliphatic organic acids (protonated and deprotonated) reacting with OH are quite high and are in the range of about 10^9^–10^10^ L mol^−1^ s^−1^. The available kinetic data indicate that the considered protonated C4 unsaturated acids (methyl crotonic acid and methacrylic acid) react almost exclusively via OH addition because of the high bond strengths for the C–H bond in the methyl and the O–H bond in the carboxyl groups. For the smaller protonated unsaturated acids, the protonated acid group likely inhibits the OH addition because the −COOH group lowers the electron density of the double bond and, therefore, leads to lower reaction constants. However, it can be concluded from the available kinetic data set that the kinetic acidity effect on unsaturated aliphatic organic acids reacting mostly via OH addition should generally be small.

Aromatic compounds may also dissociate in the case of side chains. Both protonated and deprotonated aromatic compounds are characterized mostly by very high OH reaction rate constants, up to about 10^10^ L mol^−1^ s^−1^, due to the preference of OH radicals of adding onto the aromatic ring (Adams et al., 1965). The probability of an H abstraction from the OH group, or an ETR with deprotonated acid groups, is minor. Therefore, the calculated reactivity ratios of OH reactions with aromatic compounds are around unity (0.7–2.7). This implies that there is only a small kinetic acidity effect for OH reactions with aromatic compounds. A special class of aromatic compounds, considered in Fig. 15, is imidazoles. In contrast to the acids that have been the primary focus of discussion, the partitioning of imidazoles, as a base, increases with higher acidity, and therefore, greater aqueous-phase chemical processing is feasible under more acidic particle conditions. Reaction rate constants of protonated imidazoles (Rao et al., 1975; Felber et al., 2019) are approximately a factor of 2 higher than their deprotonated forms (see Fig. 15). This indicates a minor kinetic effect of acidity.

##### NO_3_ radical oxidations

**Figure 16.**
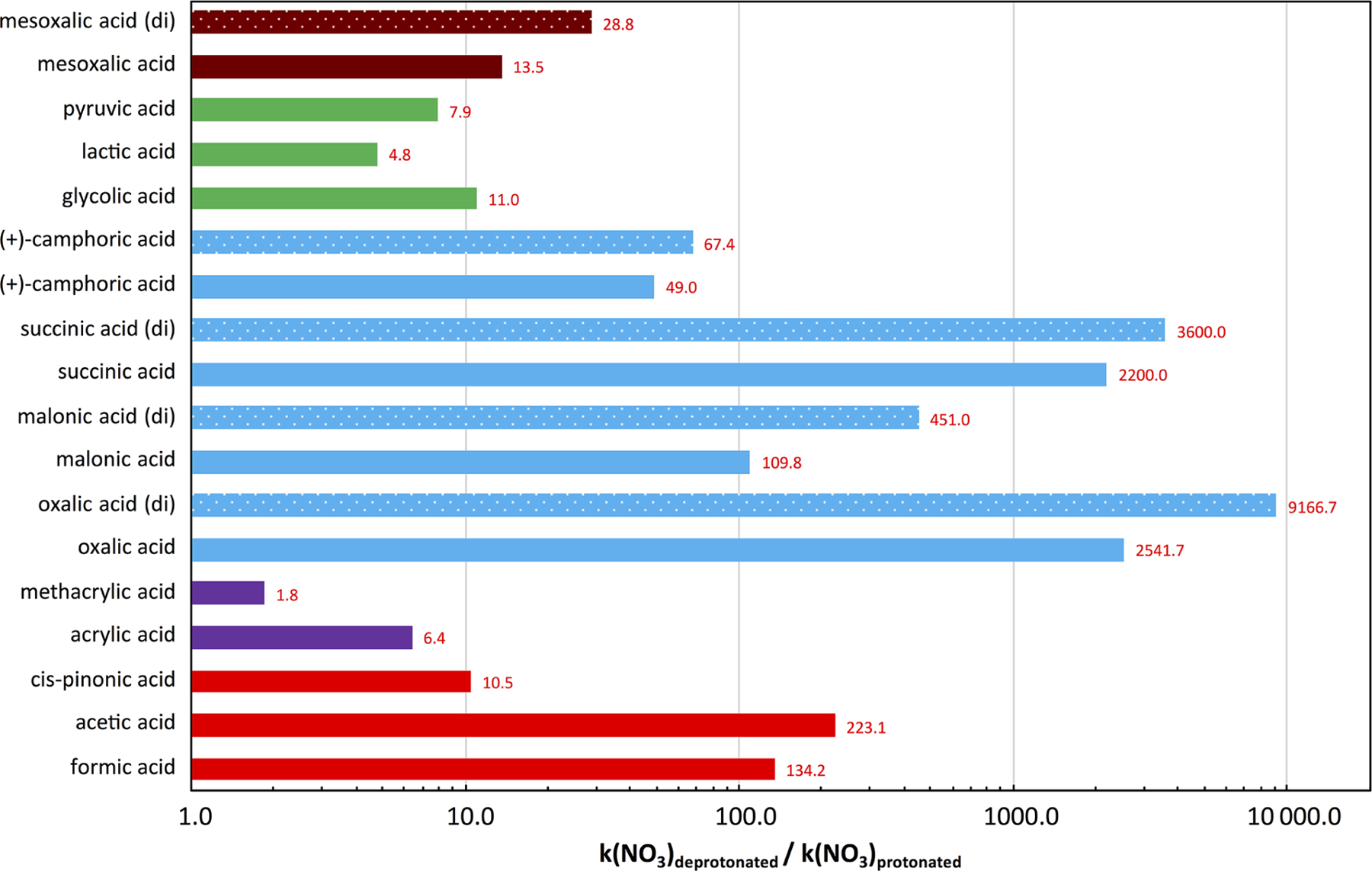
Calculated reactivity ratios *κ*_R_(NO_3_) of different carboxylic acids. The *κ*_R_(NO_3_) ratio of the dianion and protonated diacid is indicated by the add-on, (di), behind the acid name. The applied aqueous-phase NO_3_ reaction rate constants are provided in Table S6 in the Supplement. Different colors indicate different compound classes, such as unsubstituted saturated monoacids (red), unsubstituted unsaturated monoacids (purple), unsubstituted saturated diacids (blue), substituted saturated monoacids (green), and substituted saturated diacids (brown). The dotted bars mark the ratio of the dianions.

In comparison to the OH radical, the NO_3_ radical is commonly characterized by a lower reactivity, especially for plain H-abstraction reactions (Herrmann and Zellner, 1998; Herrmann et al., 2015). However, this could be compensated through the high reactivity of NO_3_ in single electron transfer reactions. As, specifically for the acids, the compiled kinetic data demonstrate, the NO_3_ radical reaction rate constants for the reactions with undissociated and dissociated acids are often quite different (see Fig. 16). Reaction rate constants of the NO_3_ radical with saturated protonated aliphatic mono- and di-carboxylic acids are typically in the range of *k* = 10^4^–10^6^ L mol^−1^ s^−1^, where the higher values correspond to rate constants for the reactions of functionalized acids. In contrast, saturated deprotonated aliphatic monoand di-carboxylic acids are oxidized by NO_3_, with rate constants typically in the range of *k* = 10^6^–10^8^ L mol^−1^ s^−1^. Accordingly, the calculated reactivity ratios *κ*_R_(NO_3_) (see Fig. 16) are often above 10 and, in some cases, up to 10^4^. As a consequence, acidity is a very important parameter when the reactivity of NO_3_ in the atmospheric aqueous phase is to be described.

Compared to saturated aliphatic acids, unsaturated aliphatic acids show higher NO_3_ reactions rate constants (*k* = 10^7^–10^8^ L mol^−1^ s^−1^). This is due to a possible addition of the NO_3_ radical to the carbon double bond, which proceeds faster than the H abstraction. *κ*_R_(NO_3_) of unsaturated acids, such as acrylic and methacrylic acid, is about 6.4 and 1.8, respectively (see Fig 16). The NO_3_ addition reaction on the C=C double bond is more important than the ETR for both the undissociated and dissociated acid. Hence, the calculated reactivity ratios are smaller compared to NO_3_ reactions of saturated acids.

Overall, it can be concluded that for NO_3_ in aqueous atmospheric systems, particularly cloud conditions, acidity can substantially affect the chemical NO_3_-initiated processing of organic compounds. Less acidic conditions will enhance the degradation of dissociating compounds via NO_3_ because of more rapid oxidation and increased partitioning (see Sect. 5.4.3 for further details). This acidity effect could be important in urban mixed regimes where higher NO_*x*_ regimes mix with marine, continental dust, or soil aerosols, which are typically less acidic. However, due to the sparse kinetic database for NO_3_ radical reactions in the aqueous phase, more kinetic and mechanistic laboratory investigations are needed, with special emphasis on acidity effects.

##### O_3_ oxidations

**Figure 17.**
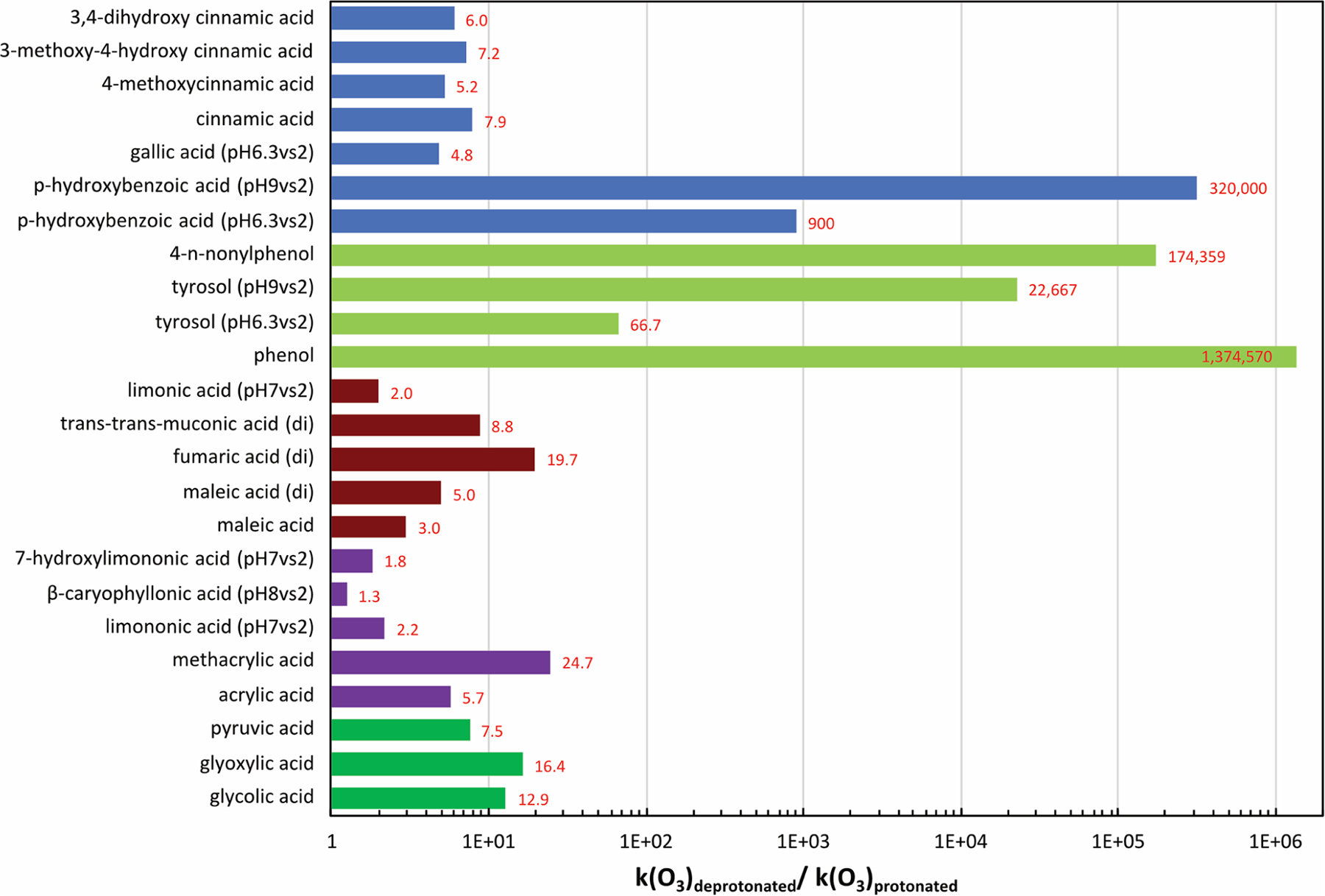
Calculated *κ*_R_(O_3_) of different dissociating organic compounds. The *κ*_R_(O_3_) ratio of the dianion and protonated diacid is indicated by the add-on, (di), behind the acid name. The applied aqueous-phase O_3_ reaction rate constants are provided in Table S7 in the Supplement. Different colors indicate different compounds classes, such as substituted saturated monoacids (green), unsaturated monoacids (purple), unsaturated diacids (brown), phenols (light green), and aromatic acids (blue).

In comparison to OH, ozone (O_3_) is commonly known to be an electrophilic and very selective oxidant for organic compounds, covering a very wide range of reactivities. It should be noted here that lower ozone rate constants might be compensated by much higher concentrations of ozone compared to OH (Tilgner and Herrmann, 2010; Schöne and Herrmann, 2014). A comparison of the O_3_ oxidation rates for protonated and deprotonated forms of dissociating compounds (Fig. 17) shows that O_3_ oxidation kinetics depend significantly on acidity, especially for phenolic compounds. For saturated carboxylic acids, carboxylates demonstrate roughly a factor of 10 higher reactivity towards O_3_ compared to the protonated acids. This higher reactivity can be explained by the higher electron-donating properties of the carboxylate. Therefore, BDEs of the carbon-bonded H atoms are smaller, making the H atoms more easily abstractable. Furthermore, ETR can also occur.

As expected, compared to saturated carboxylic acids, unsaturated carboxylic acids have significantly higher reactivities with O_3_, i.e., more than 4 orders of magnitude higher reaction rate constants. In the case of unsaturated carboxylic acids, addition to the C=C double bond will establish an important reaction pathway for both the protonated and the deprotonated unsaturated acid. Nevertheless, the calculated reactivity ratios *κ*_R_(O_3_) (see Fig. 17) show that the deprotonated unsaturated acid reacts more rapidly (1.3–25 times faster) with ozone than in its protonated form. Possible reasons could be the same as for saturated organic acids. The deprotonation likely leads to an increase in the electron density at the carbon–carbon double bond, enabling an easier O_3_ addition, i.e., a more rapid oxidation. From inductive effect theory, it is known that the COOH group is electronwithdrawing, and COO^−^ is electron-donating. Thus, the obtained behavior is feasible.

A comparison of the kinetic data of maleic and fumaric acids demonstrates that the molecular structure (symmetry and bonds) strongly affects ozone reactivity. These isomers are characterized by different physical and chemical properties (dipole moments, *pK*_a_, and reactivity). The differences in the molecular structure lead to a higher O_3_ reactivity of fumaric acid. The C=C double bond in a fumaric acid molecule is less shielded from the two acid groups, which simplifies O_3_ addition onto the double bond. Thus, a 6 times higher reactivity of the protonated fumaric acid results, compared to maleic acid.

Similar *κ*_R_(O_3_) values are also found for aromatic acids containing unsaturated carbon side chains, e.g., cinnamic acid. On the other hand, for hydroxylated acids such as p-hydroxy benzoic acid, significantly higher reactivity differences are found with decreasing acidity. Under highly acidic conditions (pH = 2), p-hydroxy benzoic acid shows a reaction rate constant with O_3_ of 2.0 × 10^2^ L mol^−1^ s^−1^ (Beltran et al., 2000), whereas it reacts much more rapidly (6.4 × 10^7^ L mol^−1^ s^−1^; Beltran et al., 2000) under alkaline conditions (pH = 9). This increase can be explained by increasing the deprotonation of the hydroxy group, leading to formation of the phenolate form and a higher contribution of the ETR with decreasing acidity. The significantly higher reactivity of the fully deprotonated form implies that the oxidation rate at a pH of 6 is still dominated by the reaction of O_3_ with the fully deprotonated form.

Rather high acidity dependencies of the reactivity data exist for phenolic compounds too (see Fig. 17), with a large increase in the ozone reaction rate of up to 6 orders of magnitude. However, it should be mentioned that the present kinetic literature data are based on extrapolations (see Hoigne and Bader, 1983b, for details). Due to the huge reactivity difference, the ozone oxidation of phenol can be dominated by the reaction with phenolate even at neutral or slightly acidic conditions. Moreover, charge transfer to ozone can lead to the formation of OH radicals (Mvula and von Sonntag, 2003), which can initiate further oxidation reactions. Thus, less acidic conditions can enhance the aqueous oxidation of phenolic compounds by dissolved ozone and, additionally, promote further oxidation due to the initiated OH chemistry.

Overall, the present O_3_ kinetic data analyses have demonstrated the crucial role of acidity for ozonolysis processes and, hence, the chemical processing of dissociating compounds in tropospheric aqueous solutions. The possible formation of OH radicals following initial ozone reactions can further enhance the oxidation capacity of the atmospheric aqueous phase. Further laboratory measurements and modeling studies are urgently needed to improve current knowledge.

#### 5.4.3 Overall considerations for the oxidation of dissociating species and the role of acidity

**Figure 18.**
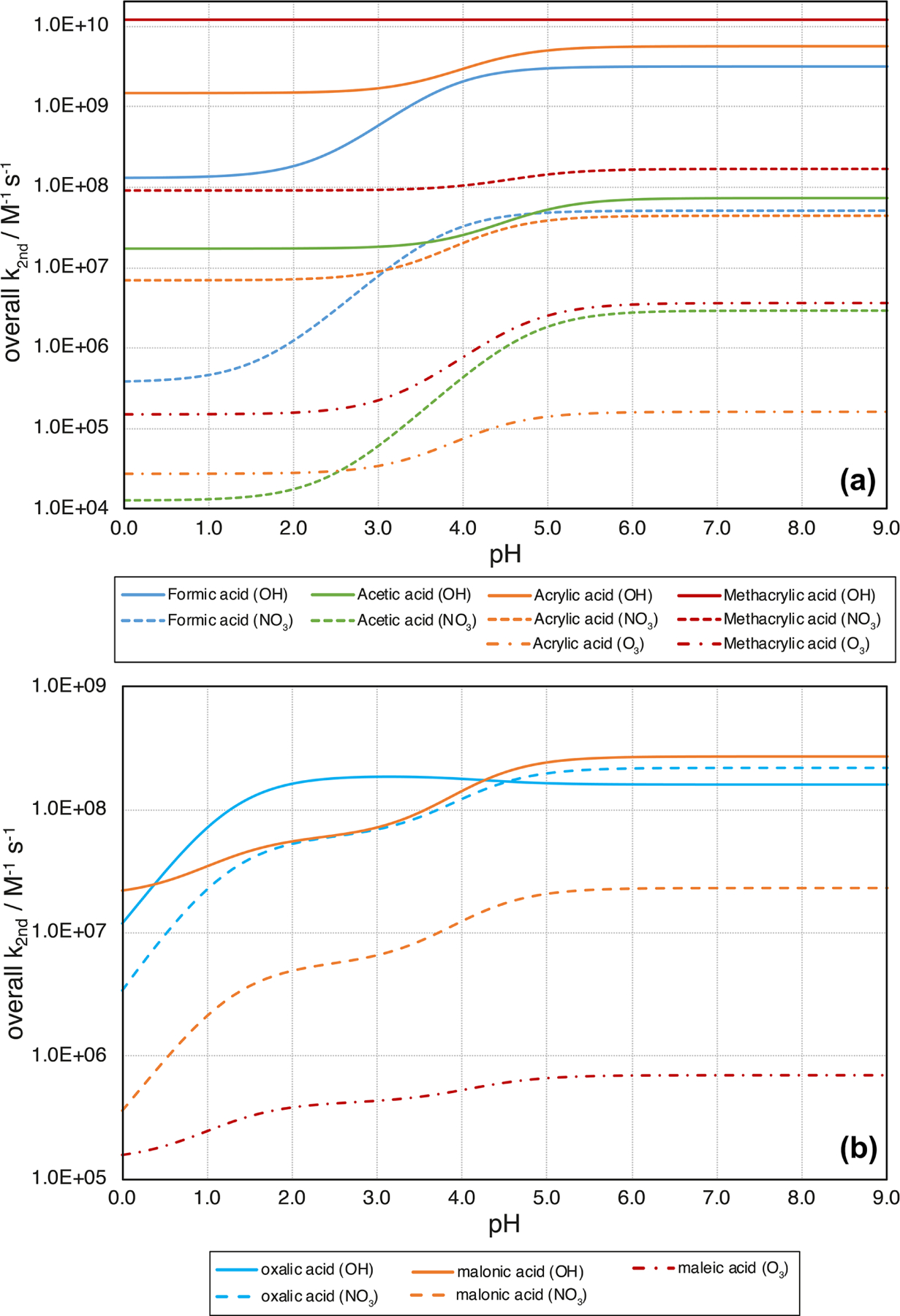
Overall condensed-phase second-order rate constant *k*_2nd_ as a function of pH of selected mono- (**a**) and dicarboxylic (**b**) acids for different radical (OH/NO_3_) and non-radical (O_3_) oxidants.

The overall reaction rate constant of a compound at a given pH depends on both the individual reaction rate constants (see above) and the degree of dissociation of the compound (which, in turn, is determined by its *pK*_a_ value(s)). The rate constants of the individual free acids and their dissociated anions represent only extreme values, and the overall processing rate for a weak acid or base will often fall between these two values, depending on the pH and the *pK*_a_. In order to illustrate this point, Fig. 18 shows the dependence of the overall reaction rate constant through a typical tropospheric pH range of 0 to 9 for a few selected mono- and diacids. The overall reaction rate constant is calculated by means of the individual reaction rate constants of the protonated and deprotonated forms and their respective speciation fraction. Please note that the overall second-order reaction rate constants consider the dissociation speciation of the carboxylic acids but not their effective solubility. Thus, the overall chemical reaction rate will depend on both the aqueous oxidant concentration and on the total aqueous compounds concentration. The latter largely depends on the microphysical conditions present.

Briefly, Fig. 18 demonstrates that the overall reaction rate constant can be largely pH dependent. This is particularly true for compounds with large reactivity ratios, i.e., those for which the anion is more reactive than the unionized form. For such compounds, the overall rate constant typically increases with increasing pH, and more efficient oxidation can be expected under less acidic conditions. In view of decreasing inorganic acid aerosol content, together with decreasing acidity in clouds in some parts of the world (Pye et al., 2020), this might imply both stronger partitioning and more efficient oxidation of organic acids (lower chemical aqueous-phase lifetime) in the troposphere under future conditions.

Overall, based on a compiled kinetic data set for oxidation by OH, NO_3_, and O_3_ of both protonated and deprotonated organic compounds, investigations of the reactivity ratio *κ*_R_ showed that, for OH reactions, the impact of acidity on the chemical kinetics is often quite small and only important for some specific compounds. For NO_3_ reactions, particularly in cloud droplets, acidity can substantially affect the chemical NO_3_-initiated processing of organic compounds, and less acidic conditions will enhance the degradation of dissociating compounds via NO_3_ because of more rapid oxidation from an increased likelihood for electron transfer reactions (ETRs). Furthermore, the present O_3_ kinetic data analyses demonstrate the crucial role of acidity for ozonolysis processes, especially for phenolic compounds.

## 6 Implications for a changing atmosphere

In the review of Pye et al. (2020), a detailed compilation of acidity data measured in cloud and fog water around the globe showed decreasing trends across North America and Europe, mainly driven by a decreased sulfate and nitrate aerosol content due to reduced anthropogenic emissions of SO_2_ and NO_*x,y*_. The reduction in fossil fuel combustion emissions in a changing world, and its related feedback on acidity, will have several implications for the chemistry-related topics discussed in the present review. As a similar trend in the acidity of aqueous aerosols particles has not yet been widely predicted by thermodynamic models, and as observations of such a trend for aerosol particles are scarce (see Pye et al., 2020), this section will mainly focus on the implications of changes in the acidity of cloud and fog on multiphase chemistry.

As a result of reductions in anthropogenic emissions of acid precursors in many western industrialized countries, the relative contributions of other sources to the acidification in fog and cloud droplets will continue to grow in importance over the next few decades, unless emissions of ammonia from agricultural fertilization are simultaneously reduced. Other direct and indirect acid sources are, for example, (1) the emission of dimethyl sulfide (DMS), (2) the emission of SO_2_ from volcanic activity, (3) the sea-spray-aerosol-related emission of HCl/Cl^−^, and (4) the emission and secondary multiphase formation of organic acids, such as formic and oxalic acid.

On the one hand, at pH ranges between 4 and 6, weaker acids tend to partition into less acidic cloud and fog waters more effectively and, thus, contribute more substantially to acidity in less acidic droplet waters. On the other hand, less acidic cloud and fog water pH values are in the typical range of the *pK*_a_ values of weak acids, such as acetic acid (*pK*_a_ = 4.75), so that they can efficaciously buffer acidification by stronger acids in this acidity range (see Sect. 2.2). As a consequence, the increased aqueous-phase partitioning enables higher chemical processing rates of weak acids such as SO_2_, HONO, and organic carboxylic acids. Both lower acidity and stronger buffering can support faster S(IV) to S(VI) conversions due to the higher efficiency of other chemical pathways, such as ozone oxidation (Li et al., 2020b), and, therefore, reduce the tropospheric lifetime (and in-cloud lifetime) of SO_2_. Thus, under future conditions with a lower overall SO_2_ burden, the increased secondary sulfate mass formation probabilities may compensate, at least partly, for the reduced sulfate formation potential. In the case of organic acids, the increased in-cloud partitioning allows greater opportunities for chemical processing, leading to higher formation yields of functionalized organic acids, which tend to partition even stronger towards the aqueous phase of particles and droplets. Hence, higher in-cloud SOA formation yields can be expected as a consequence of the lower acidification of cloud and fog waters by anthropogenic sulfate.

Having affected in-cloud chemistry processes, the decreasing SO_2_ burden will also presumably influence the isoprene-related SOA formation, particularly the OS formation. Here, several projection studies (Pye et al., 2013; Marais et al., 2016; Budisulistiorini et al., 2017; Q. F. He et al., 2018; Zhang et al., 2018a) have proposed a reduced IEPOX-derived SOA formation under reduced SO_2_ emissions. Also, studies for the southeastern USA (Pye et al., 2013; Marais et al., 2016; Budisulistiorini et al., 2017) implied that a reduction in SO_2_ by 25 %–48 % led to reduction in the IEPOX-derived SOA formation of about 35 %–70 %. This effect is mainly related to the changes in aerosol acidity but could be further modulated by the resulting changes in particle viscosity and phase separation that result from the extensive conversion of inorganic to organic sulfur expected with declines in SO_2_ (Riva et al., 2019). For the Pearl River Delta region, a reduction of ~45 % of the IEPOX-derived SOA was reported by Q. F. He et al. (2018) due to an aerosol sulfate reduction by 25 %. Finally, all studies clearly demonstrated that a SO_2_ emission decline in polluted regions could significantly lower the isoprene-related SOA. Similar effects can also be assumed for other acid-catalyzed or acidity-dependent processes.

Another chemical subsystem that will likely be affected by reduced anthropogenic acid precursor emissions in the future is TMI solubilization (see Pye et al., 2020, and references therein). The smaller possible acidification of aqueous interfacial layers on crustal aerosols can lower the acid-driven solubilization of TMI, particularly in regions where dust particles are mixed with urban pollutants. The decreased formation of soluble and, hence, bioavailable TMIs can (1) cause lower nutrient inputs into oceans, impacting the ocean biological activity there, (2) decrease the chemical HO_*x*_ radical cycling in both aqueous particles and droplets, and (3) may also affect the TMI-related S(IV) oxidation.

Decreasing atmospheric acidity may also impact the acidity-driven production of reactive halogens, with potential implications for ozone and OH. Observations of sea salt aerosol bromide and chloride deficits over the northeastern Pacific Ocean revealed that depletions in bromide and chloride relative to seawater were correlated to particle acidity (see, e.g., Newberg et al., 2005).

In order to explore the expected changes in the tropospheric multiphase chemistry and their overarching impacts in a changing environment, further field measurements, laboratory studies, and accompanied modeling are needed to both monitor occurring changes and improve air quality and climate model predictions.

## 7 Conclusions and outlook

In the present review, we have outlined different aspects and chemical subsystems in which acidity affects multiphase chemistry and, in turn, acidity is affected by tropospheric multiphase chemistry. Although many advances have been made in our understanding of acidity-driven and acid-catalyzed chemical processes, there are still many open issues which need to be addressed in order to further advance our understanding of the complex role of acidity in the atmosphere. Besides the implications caused by changing acidity conditions in the atmosphere (cf. Sect. 6), the present review has also identified chemistry-related research targets and needs for further investigation that are outlined below. Specifically, these chemistry-related future research needs and objectives are as follows:

1. To advance our understanding of the activation mechanisms and multiphase chemical processing of reactive halogen species in different acidity environments and to quantify their presence in and above the tropospheric boundary layer
2. To undertake more sophisticated field, laboratory, and model investigations on the importance of different S(IV) oxidation pathways under various acidity conditions in different environments, particularly under conditions typical of aerosols (lower pH and high ionic strength)
3. To advance our understanding of aqueous-phase organic accretion reactions, including their dependence on acidity in order to assess their role for the secondary aerosol formation in clouds and aqueous particles
4. To perform advanced kinetic and mechanistic studies on the acidity dependencies of aqueous-phase organic oxidations
5. To quantify the role of organosulfates (OS), organonitrates (ON), and/or nitrooxy organosulfates (NOS) as potential acidity reservoirs or sinks and to characterize the role of acidity for their formation and fate in aerosols
6. To investigate the impact of particle acidity on the formation and early growth of tropospheric nanometer-sized particles from highly oxidized molecules (HOMs)
7. To improve size- and time-resolved cloud and fog measurement techniques and to develop in situ measurements techniques for directly determining aerosol acidity, burdens which still limit our knowledge about the impact of acidity on the multiphase chemistry in different droplet and aerosol sizes and their feedbacks on the chemical composition.

With reference to point (1) above, while much effort has been devoted to investigations of reactive halogen chemistry in pristine open-ocean regions and partly coastal areas (see Sect. 4.8 for details), the impact of reactive halogens on atmospheric chemistry in developing countries is less examined. Especially in developing economies such as China and India, where a substantial amount of the air pollution is related to coal combustion and other biomass burning, a significant fraction of the aerosol matter consists of halogens related to such sources (Goetz et al., 2018; Gani et al., 2019). Further studies are needed to investigate the role of halogen chemistry in strongly polluted environments which are characterized by very acidic particles compared to marine environments. Under very polluted conditions, high acidity linked with high NO_*x,y*_ can cause active halogen radical chemistry that might influence the tropospheric cleaning capacity. However, the role of multiphase halogen chemistry in such environments is still not well investigated. For example, the recent study of X. Wang et al. (2019) has demonstrated that current models are not able to reproduce high observed Cl_2_ in the daytime in continental regimes. Furthermore, the recent model studies of Zhu et al. (2019) also reported over-estimates of free tropospheric BrO during the extratropical winter–spring. Moreover, our understanding of halogen activation processes in Arctic regions needs further improvement. Arctic regions are undergoing unprecedented climate changes, likely with substantial changes in the aerosol composition that can affect the aerosol acidity and, consequently, halogen activation processes. So, further research is needed to focus on how Arctic climate changes will impact halogen chemistry in this dynamic and sensitive environment.

Regarding point (2) above, comparisons of model findings with field measurements in polluted regions have shown that current models often underestimate the S(VI) formation rates or cannot reproduce the findings of sulfur isotope measurements regarding the responsible oxidation pathways. Hence, the current chemical kinetic and mechanistic understanding of the S(IV) to S(VI) conversion processes, including their acidity dependency, is still incomplete for adequately predicting the budgets of S(VI) in cloud, fog, and, especially, aqueous aerosol conditions. As multiphase chemistry models rely on detailed acidity-dependent kinetic and mechanistic knowledge, further laboratory studies are indispensable for improving model predictions of S(VI), particularly under conditions of high ionic strength (e.g., aerosol chamber or aerosol flow tube studies).

As outlined in point (3) above, non-oxidative aqueous organic chemical processes, such as accretion reactions (aldol condensation, hemiacetal and acetal formation, and the esterification of carboxylic acids), are affected by the acidity and/or basicity of the solution and are expected to be important formation pathways of aqueous-phase-related SOA (aqSOA). However, the potential role of such acidity-related processes in tropospheric aqueous solutions is still not yet fully explored since mechanistic and, particularly, kinetic data on acid-catalyzed accretion reactions in aerosols are still sparse (see Herrmann et al., 2015). So, these acidity-related processes should be a key objective of future laboratory and chamber studies towards a better representation of such processes in detailed chemistry mechanisms and models.

Furthermore (see point 4 above), more advanced kinetic and mechanistic studies on the acidity dependencies of aqueous-phase oxidations of dissociating organic compounds, such as functionalized carboxylic acids, are needed to better describe such processes in future multiphase models and to, finally, elucidate their impacts. Compared to the huge number of functionalized organic dissociating compounds that are formed by various multiphase reaction pathways, the body of investigated aqueous-phase oxidations are still rather small. Furthermore, existing estimation methods for aqueous-phase kinetic reaction rate constants are either characterized by large uncertainties, particularly for functionalized compounds (Bräuer et al., 2019), or do not exist because of the sparse data set available. Even though the present review showed that acidity could play an important role in the ozonolysis processes of dissociating compounds in tropospheric aqueous solutions, the kinetic and mechanistic knowledge of such oxidation processes, including the possible formation of OH radicals, is, nevertheless, currently rather limited. Further kinetic and mechanistic laboratory investigations are urgently needed to minimize the current enormous knowledge limitations and uncertainties, for example, with regards to the production of organic mono- and dicarboxylic acids, as well as their functionalized derivatives.

Moreover (see point 5 above), organosulfates (OSs) are ubiquitous constituents of atmospheric aerosol particles that not only contribute substantially to organic matter (OM) but may also bind a considerable portion of the sulfate content of atmospheric particles (Riva et al., 2019; Brüggemann et al., 2020). For this reason, these compounds have the potential to reduce the free sulfate and, consequently, the H^+^ formation potential, with major implications for aerosol acidity. Overall, OSs may be a temporary acidity reservoir due to the binding and release of sulfate or a sink of H^+^, if H^+^ is incorporated into the OS and unavailable for further processing. While progress has been achieved in the understanding of OS formation pathways in the last decade, the scientific understanding of their chemical processing in aqueous aerosols is still uncertain. In order to elucidate the role of OSs for acting as crucial acidity reservoirs and/or buffers in atmospheric particles, more detailed knowledge of their chemical processing is highly desirable. Thus, the chemical transformations of OSs through hydrolysis and oxidations by atmospheric radicals (e.g., OH, NO_3_, Cl, etc.) require more kinetic and mechanistic laboratory investigations as reaction data are usually not yet available. Additionally, similar investigations on the formation and transformation must be performed for organonitrates (ONs) and nitrooxy organosulfates (NOSs), which could also potentially act as nitrate and sulfate reservoirs and, thus, affect the acidity.

Historically (see point 6 above), sulfuric acid has been considered the driver of new particle formation events (e.g., Sipila et al., 2010). However, several studies (see Lee et al., 2019, and references therein) have shown that the gas-phase oxidation of biogenic and anthropogenic VOCs, via autoxidation reactions, can produce highly oxidized organic molecules (HOMs). HOMs are characterized by extremely low-saturation vapor pressures and can effectively condense on nanometer-sized aerosol, contributing there to the early growth of particles. Due to their high degree of oxygenation, including several chemical functional groups (e.g., peroxide, hydroperoxide, carbonyl, and carboxylic acid) that are sensitive to acidity, they can undergo chemical reactions there which can enhance their uptake and contribution to the particle growth. As a result, one possible chemical pathway would be the formation of HOOSs (highly oxidized OSs). Having first been reported by Mutzel et al. (2015), they potentially serve as an example to better explain the early growth of freshly formed particles. Such acidic aerosols might provide a chemical environment in which extremely low volatility compounds can condense and react, leading to the formation of HOOSs. Elucidating the role of acidity for the formation of SOA in such small particles and their importance for early nanoparticle growth will be a crucial objective for future field and chamber studies.

Finally (see point 7 above), because aerosols are ubiquitous in the atmosphere and cloud cover 60 % of the Earth, understanding their contribution to tropospheric composition and how it is evolving is crucial. Here, the present review has shown the role of acidity in determining aqueous chemistry, and vice versa, yet several issues demand more advanced field, kinetic laboratory, chamber, and modeling studies. Armed with this new fundamental knowledge, better predictions can be made of aerosol, cloud, and fog chemistry on tropospheric oxidizing capacity, air quality, climate, and human health. For example, to better characterize the effect of varying acidity on chemical processing and the identification of potential changes due to changing anthropogenic emissions, further advances in measuring the chemical composition of cloud and fog, in both a size- and time-resolved manner, is needed. The lack of measurement and analytical techniques for directly determining aerosol acidity in situ is even more urgent.

## Supplementary Material

Supplement1

## Figures and Tables

**Table 1.** Composition conditions applied for the calculation of the S(IV) oxidation rates of different reaction pathways for urban haze and rural aerosol conditions, as well as urban and rural cloud conditions (bottom) at 298 K.

Reactant	Concentration			
	Urban haze	Rural aerosol	Urban cloud	Rural cloud
SO_2_/ppb	40.0^b^	1.0^e^	5.0^a^	1.0^e^
H_2_O_2_/ppb	0.1^d^	0.1^e^	1.0^a^	0.1^e^
O_3_/ppb	1.0^b^	30.0^e^	50.0^a^	30.0^e^
NO_2_/ppb	66.0^b^	1.0^e^	10.0^e^	1.0^e^
HNO_4_/ppb	0.01^e^	0.001^e^	0.01^e^	0.001^e^
CH_3_OOH/ppb	0.1^e^	0.1^e^	0.1^e^	0.1^e^
CH_3_C(O)OOH/ppb	0.1^e^	0.1^e^	0.1^e^	0.1^e^
Fe(III)/mol L^−1^	1.1 × 10^−3b^	1.0 × 10^−3a^	1.0 × 10^−5c^	1.0 × 10^−6c^
Mn(II)/mol L^−1^	2.55 × 10^−3b^	1.0 × 10^−4a^	1.0 × 10^−6c^	1.0 × 10^−7c^
PS*/mol L^−1^	1.9 × 10^−11^	6.3 × 10^−11^	1.2 × 10^−12^	3.6 × 10^−13^
Ionic strength/mol L^−1^	1.0^e^	1.0^e^	1.0 × 10^−4e^	1.0 × 10^−4e^

a Based on Seinfeld and Pandis (2006).

b Based on Cheng et al. (2016).

c Estimated from data given in Deguillaume et al. (2005).

d Based on C. Ye et al. (2018).

e Estimated daytime particle and cloud mean concentrations based on simulations using the chemical aqueous phase radical mechanism (CAPRAM; Bräuer et al., 2019; Hoffmann et al., 2020). Further simulation details are given in the Supplement. Note: ppb – parts per billion.

**Table 2.** Influence of acidity on the hydration rate of formaldehyde and acetaldehyde in buffered solutions.

Acidic species	Acid dissociation constant of the catalyst acid–base pair *K*_a_ (unitless)	Acid catalytic constant *k*_a_ (L mol^−1^ s^−1^)	Base catalytic constant *k*_b_ (L mol^−1^ s^−1^)
Formaldehyde^a^			
H^+^	55.5	2.7	0.0051
Formic acid	1.77 × 10^−4^	0.070	0.013
Phenylacetic	4.88 × 10^−5^	–	0.015
Acetic acid	1.75 × 10^−5^	0.043	0.022
Trimethylacetic acid	8.9 × 10^−6^	0.025	0.022
Water	1.8 × 10^−16^	0.0051	1600
Acetaldehyde^b^			
H^+^	55.5	930	0.00014
Formic acid	1.77 × 10^−4^	1.74	0.065
Phenylacetic	4.88 × 10^−5^	0.91	0.054
Acetic acid	1.75 × 10^−5^	0.47	0.157
Trimethylacetic acid	9.4 × 10^−6^	0.33	0.161
Water	1.8 × 10^−16^	0.00014	8 × 10^−4^

a Bell and Evans (1966).

b Kurz (1967), Kurz and Coburn (1967), and Ogata and Kawasaki (1970).

## Data Availability

All data used for the figures are provided in the tables of this paper and in the Supplement.
