# Supplementary material for: Acidity and the multiphase chemistry of atmospheric aqueous particles and clouds
Source: Atmos Chem Phys. Author manuscript; Available in PMC 2022 Sep 10. (PMC8525431; doi:10.5194/acp-21-13483-2021)
Supplement: Supplement1 [file NIHMS1745324-supplement-Supplement1.pdf]

Supplement of Atmos. Chem. Phys., 21, 13483–13536, 2021
https://doi.org/10.5194/acp-21-13483-2021-supplement
© Author(s) 2021. CC BY 4.0 License.

Supplement of

Acidity and the multiphase chemistry of atmospheric aqueous particles and clouds

Andreas Tilgner et al.

Correspondence to: V. Faye McNeill (vfm2103@columbia.edu) and Hartmut Herrmann (herrmann@tropos.de)

The copyright of individual parts of the supplement might differ from the article licence.

Supplementary material to section 2

Table S1: Summary of the applied H_A and pK_a values for calculation of the LWC-acidity dependent aqueous fraction $X_{A_{aq}}$ presented in Figure 2, Figure 3 and Figure S1.

Compound	H_A (298K) M atm^{-1}	Reference	pK_{a1}	pK_{a2}	Reference
Sulfurous acid (dissolved SO_2)	1.32	Sander (2015)	1.9	7.0	Kolthoff and Elving (1959)
Nitrous acid HONO	48.6	Park and Lee (1988)	3.29	—	Kolthoff and Elving (1959)
Formic acid HCOOH	5530	Khan et al. (1992)	3.77	—	Braude et al. (1955)
Acetic acid CH_3COOH	5471	Khan et al. (1992)	4.76	—	Haynes (1958)
Glycolic acid $CH_2OHCOOH$	$2.83 \cdot 10^4$	Ip et al. (2009)	3.83	—	Zirchrom (2018)
Phthalic acid $C_6H_4(COOH)_2$	$4.96 \cdot 10^7$	TOXNET (2015)	2.98	5.28	Braude et al. (1955)
2-nitrophenol $C_6H_5NO_3$	$1.47 \cdot 10^2$	Guo and Brimblecombe (2007)	7.21	—	Rappoport (1984)
2,4-dinitrophenol $C_6H_4N_2O_5$	$1.11 \cdot 10^4$	Tremp et al. (1993)	4.09	—	Rappoport (1984)
Ammonia NH_3	59.8	Christie and Crisp (1967)	9.24		Bjerrum et al. (1958)
Dimetyhlamine $CH_3)_2NH$	56.5	Burkholder et al. (2015)	10.4		Braude et al. (1955)

Supplementary material to section 4

Table S2. Rate constants and equilibrium constants for reaction of NO_2 with S(IV).

	SO_3^{2-} (pH = 10)			HSO_3^- (pH = 4.5)		
**T / K**	**k_{10a} / L mol^{-1} s^{-1}**	**k_{-10a} / s^{-1}**	**K_{10a} / L mol^{-1}**	**k_{10b} / L mol^{-1} s^{-1}**	**k_{-10b} / s^{-1}**	**K_{10b} / L mol^{-1}**
288	$(1.5 \pm 2) \times 10^7$	$(8 \pm 3) \times 10^2$	1.9×10^4	$(1\ ^{-1}/_{+3}) \times 10^6$	$(1.7 \pm 0.2) \times 10^2$	5.9×10^3
298	$(1.2 \pm 2) \times 10^7$	$(9 \pm 2) \times 10^2$	1.3×10^4	$(1\ ^{-1}/_{+3}) \times 10^6$	$(2.3 \pm 0.2) \times 10^2$	4.4×10^3
308	$(1.7 \pm 2) \times 10^7$	$(1.2 \pm 0.3) \times 10^3$	1.4×10^4	$(2\ ^{-2}/_{+3}) \times 10^6$	$(3.7 \pm 0.3) \times 10^2$	5.4×10^3
318	$(1.4 \pm 2) \times 10^7$	$(1.6 \pm 0.2) \times 10^3$	8.8×10^3	$(4\ ^{-4}/_{+4}) \times 10^6$	$(5.8 \pm 0.4) \times 10^2$	6.9×10^3
328	$(1.2 \pm 2) \times 10^7$	$(2.2 \pm 0.2) \times 10^3$	5.5×10^3	$(6\ ^{-6}/_{+13}) \times 10^6$	$(7 \pm 1) \times 10^2$	8.6×10^3

Remarks: Since the measurement error of k_{10a} does not allow to derive an Arrhenius expression, the temperatures were averaged from 288 K to 328 K, with a specified error calculated using the standard deviation. The reported errors of the Arrhenius expressions represent the statistical error using the Student's t-distribution for a 95% confidence interval. The T-dependence of the equilibrium constant K_{10b} of the NO_2 reaction with HSO_3^- was calculated without considering the result at T = 288 K.

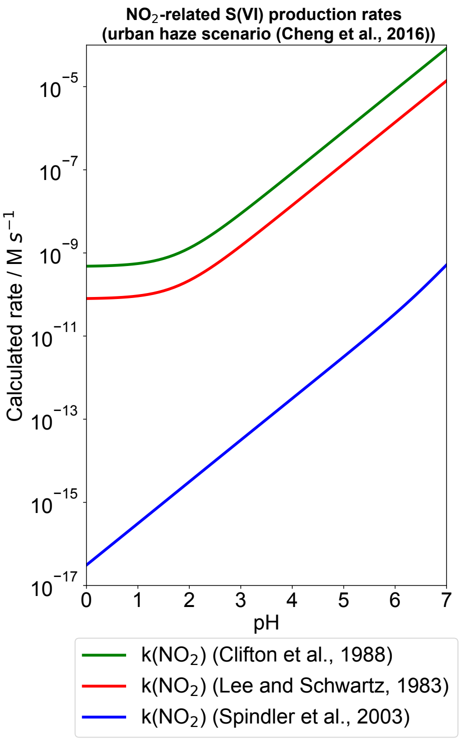

Figure S1. Comparison of the calculated S(IV) oxidation rates by the NO_2 reaction pathway in mol L^{-1} s^{-1} using the different reaction rate constants of Lee and Schwartz (1983), Clifton et al. (1988) and Spindler et al. (2003) for the urban winter haze scenario of Cheng et al. (2016).

Determination of aqueous-phase concentrations of excited triplet states of photosensitizers (PS*)

To calculate initial S(IV) oxidation rates of PS* and compare their potential atmospheric relevance compared to other S(IV) to S(VI) conversion pathways under different environmental and acidity conditions, realistic aqueous-phase concentration levels of PS* were determined by means of multiphase chemistry simulations using the CAPRAM mechanism (Bräuer et al., 2019; Hoffmann et al., 2020; Ye et al., 2021). Simulations with CAPRAM mechanism have been performed for urban winter haze conditions (model period 4 (Dec. 27th-31st) based on (Ye et al., 2021)) as well as the CAPRAM standard summer remote and urban scenarios (Tilgner et al., 2013; Bräuer, 2015; Bräuer et al., 2019). A simple chemical mechanism scheme with 12 reactions was developed and applied in the SPACCIM model (see Fig. S2 below) considering the photochemical formation of the excited triplet states of photosensitizers (PS*) and their main loss processes.

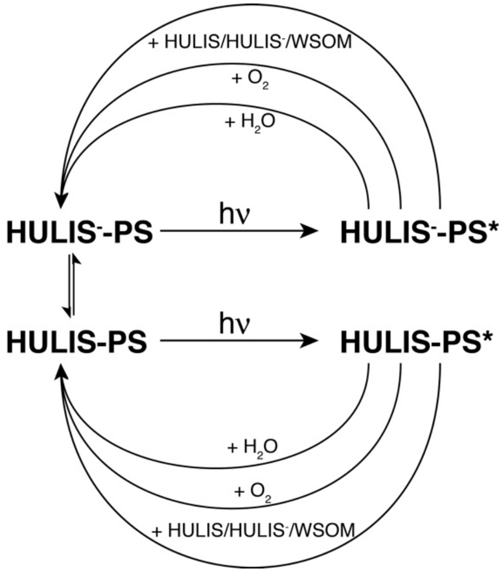

Figure S2. Chemical pathways of PS* considered in the reaction module.

For the simulations, it was assumed that humic-like substances (HULIS) can act as potential photosensitizers. The initial photochemical formation of excited triplet states of photosensitizers (PS*) was implemented based on the photochemical data of m-methoxybenzaldehyde from recent measurements at TROPOS (Felber et al. (2021), photochemical implementation following the MCM, $I = 1.72 \cdot 10^{-1}$ s^{-1}, $M = 0.765$, $N = 0.214$). Additionally, subsequent quenching reactions with water ($k_{1st} = 1 \cdot 10^5$ s^{-1} (Sharpless, 2012; Erickson et al., 2018; Ossola et al., 2019; Felber et al., 2020)), dissolved oxygen ($k_{2nd} = 1 \cdot 10^9$ M^{-1} s^{-1} (Zepp et al., 1985; Canonica et al., 1995; Schweitzer and Schmidt, 2003; Montalti et al., 2006; Canonica, 2007; Erickson et al., 2018; Kaur and Anastasio, 2018; Felber et al., 2020)) and dissolved organic matter ($k_{2nd} = 1 \cdot 10^8$ M^{-1} s^{-1} (Canonica, 2007; Wenk et al., 2013; Erickson et al., 2018; Moor et al., 2019)) were included. The applied second-order quenching rate constants represents conservative estimates based on typical values reported in the literature given above. However, it should be noted that literature values especially for the quenching with dissolved organic matter show a huge

variation with typical values between 10^7 and up to few 10^9 M^{-1} s^{-1} (see above-mentioned references). Moreover, the simple mechanism scheme does not consider any triplet reactions with inorganics such as halides due to their very compound and triplet specific reactivity (see e.g. (Treinin and Hayon, 1976; Loeff et al., 1992; Loeff et al., 1993; Tinel et al., 2014)). Consequently, the calculated concentrations represent upper limit values for the steady-state concentrations of PS*.

Finally, daytime mean concentrations were calculated for urban winter haze, rural aerosol and rural/urban cloud conditions based on the modelled PS* concentration profiles (see Table 1 in the main manuscript text) and applied for the comparison of the different S(IV) to S(VI) conversion pathways (see Fig 7). The obtained daytime mean concentrations, ranging from about few 10^{-11} mol L^{-1} under deliquesced aerosol conditions to $10^{-13} - 10^{-12}$ mol L^{-1} under cloud conditions, are in a reasonable agreement with reported triplet concentrations in aerosols ($2.3 \cdot 10^{-13} - 1.6 \cdot 10^{-10}$ mol L^{-1} estimated by Wang et al. (2020)), in fog samples ($0.07 - 1.5 \cdot 10^{-13}$ (Kaur and Anastasio, 2018)) and in natural surface waters ($10^{-14} - 10^{-13}$ (Zepp et al., 1985; Canonica et al., 1995)).

Supplementary material to section 5

Table S3. Overview of determined hydration equilibrium constants $K_{Hyd.}$ of aldehydes and ketones. Recommended values are in bold.

$K_{hydration}$	T /K	Comment	Reference
		Aldehydes	
**Formaldehyde**			
2000	298	Review	Bell (1966)
2000	298	Review	Ogata and Kawasaki (1970)
2190	293	UV/VIS, T-dependent, pH = 5 – 9	Zavitsas et al. (1970)
2220	295	Chemical trapping, pH = 4 – 7	Sutton and Downes (1972)
2420	298	UV/VIS, p-dependent	Lewis and Wolfenden (1973)
2270	298	UV/VIS, NMR spectroscopy	McDonald and Martin (1979)
**2000**	**298**	**Review**	**Doussin and Monod (2013)**
2100	293	NMR spectroscopy, pH = 1 – 8, T-dep.	Rivlin et al. (2015)
**Acetaldehyde**			
1.43	298	Review	Bell (1966)
1.06	298	UV/VIS, T-dependent	Kurz (1967)
1.2	296	UV/VIS, NMR spectroscopy	Ahrens and Strehlow (1965); Bell (1966)
1.2	298	UV/VIS, p-dependent	Lewis and Wolfenden (1973)
1.2	298	UV/VIS, Chemical trapping	Sørensen and Jencks (1987)
1.19	298	Review	Tur'yan (2000)
**1.2**	**298**	**Review**	**Doussin and Monod (2013)**
**Propanal (Propionaldehyde)**			
0.71	298	Review	Bell (1966)
0.71	298	NMR spectroscopy	Greenzaid et al. (1967a)
0.708	308.65	NMR spectroscopy	Socrates (1969)
0.714	298	UV/VIS, p-dependent	Lewis and Wolfenden (1973)
0.83	298	Stopped flow measurement, pH = 1 – 6	Buschmann et al. (1982)
**0.85**	**298**	**Review**	**Doussin and Monod (2013)**
**Butanal**			
0.43	298	Review	Bell (1966)
0.48	298	NMR spectroscopy	Greenzaid et al. (1967a)
0.426	298	UV/VIS, p-dependent	Lewis and Wolfenden (1973)
**0.60**	**298**	**Review**	**Doussin and Monod (2013)**
**Pivaldehyde**			
0.1	298	UV/VIS, pH = 4.6	Lienhard and Jencks (1966)
0.24	298	NMR spectroscopy	Greenzaid et al. (1967a)
0.244	298	UV/VIS, p-dependent	Lewis and Wolfenden (1973)
**0.23**	**298**	**Review**	**Doussin and Monod (2013)**
**Glyoxal (1st hydration)**			
**207**	**298**	**Review**	**Doussin and Monod (2013)**

**Glyoxal (2nd hydration)**			
**20000**	**298**	**Review**	**Doussin and Monod (2013)**
**Methylglyoxal (1st hydration)**			
2700	298	Polarography	Wasa and Musha (1970)
565	298	Terminal gem-diol, NMR spectroscopy	Creighton et al. (1988)
1279	293	Polarography	Montoya and Mellado (1994)
2000	298	Review	Doussin and Monod (2013)
**1515**	**298**	**Review, average from 3 values**	**This work**
**Methylglyoxal (2nd hydration)**			
**0.5**	**298**	**Second gem-diol, NMR spectroscopy**	**Creighton et al. (1988)**
**Glycolaldehyde**			
25	298	Review	Bell (1966)
**15.7**	**298**	**Review**	**Doussin and Monod (2013)**
**Acetone**			
0.002	298	Review	Bell (1966)
0.002	298	NMR spectroscopy	Greenzaid et al. (1967a)
**0.002**	**298**	**Review**	**Doussin and Monod (2013)**
**Diacetyl, (2,3-butandione)**			
3.3	298	Review	Bell (1966)
0.244	298	UV/VIS, p-dependent	Lewis and Wolfenden (1973)
2.1	298	Stopped flow measurement, pH = 1 – 3	Buschmann et al. (1982)
**2**	**298**	**Review**	**Doussin and Monod (2013)**
**2-imidazol-carboxaldehyde**			
**125**	**not reported**	**pH = 1.5, NMR spectroscopy, pH-dep.**	**Ackendorf et al. (2017)**
**0.043**	**not reported**	**pH = 9, NMR spectroscopy, pH-dep.**	**Ackendorf et al. (2017)**

Table S4. Overview of determined hydration equilibrium constants $K_{Hyd.}$ of α-oxocarboxylic acids.

$K_{hydration}$	T /K	Comment	Reference
Glyoxylic acid			
1100	298	Review	Tur'yan (1998)
**1100**	**298**	**Review**	**Doussin and Monod (2013)**
Glyoxylate			
19	298	NMR spectroscopy, in D_2O, pH = 6	Copper and Redfield (1975)
67	298	Review	Tur'yan (1998)
54.6		NMR spectroscopy, room temperature	Leitzke et al. (2001)
**67**	**298**	**Review**	**Doussin and Monod (2013)**
Pyruvic acid			
2.38	298	Review referring Strehlow (1962)	Bell (1966)
2.4	298	Review	Tur'yan (1999)
1.4	298	Review	Doussin and Monod (2013)
2.35	298	NMR spectroscopy, in 10% D_2O, pH = 0, ionic strength 0.15, pH = 0 – 6 (cf. Fig. 11)	Lopalco et al. (2016)
1.0, 1.3, 1.5	296	NMR spectroscopy, in 0.02, 0.05 and 0.1 M pyruvic acid, pH = 2.06	Reed Harris et al. (2014)
0.98<br>0.29	296	in 0.01 M pyruvic acid, pH = 2.2, NMR spectroscopy,<br>in 0.0005 M pyruvic acid, pH = 3.5	Rapf et al. (2017b)
0.89	296	in 0.01 M pyruvic acid, at pH = 2.2, NMR spectroscopy, pH-dep. (cf. Fig. 11)	Rapf et al. (2017a)
**2.4**	**298**	**pH = 0 (cf. Tur'yan (1999) and Lopalco et al. (2016))**	**This work**
Pyruvate			
not hydrated		IR spectroscopy, pH = neutral	Jencks and Carriuolo (1958)
0.054	298	NMR spectroscopy, in 2 M pyruvic acid	Becker (1964)
not hydrated	298	UV/VIS, pH = 5.2	Lienhard and Jencks (1966)
0.054	298	NMR spectroscopy	Greenzaid et al. (1967a)
0.011<br>0.053	303	in 10% D_2O, NMR spectroscopy<br>in 20% D_2O, NMR spectroscopy	Öjelund et al. (1967)
0.09	298	Pulse polarography	Fonds et al. (1969)
0.04	296.7	NMR spectroscopy, pH = 6.5	Patting and Strehlow (1969)
0.087	298	NMR spectroscopy, in D_2O, pH = 6	Copper and Redfield (1975)
0.075	311	NMR spectroscopy, in 0.5 M pyruvic acid in D_2O	Kokesh (1976)
0.06	298	Review	Doussin and Monod (2013)
0.087	298	NMR spectroscopy, in 10% D_2O, pH = 6, ionic strength 0.15, pH = 0 – 6 (cf. Fig. 11)	Lopalco et al. (2016)
0.13	296	in 0.01 M pyruvic acid, at pH = 7, pH-dep.	Rapf et al. (2017a)
**0.08**	**298**	**Average of all values at 298 K at pH = 6**	**This work**
Mesoxalic acid (α-Keto acid)			
100	298	Pressure jump method, 0.1 M mesoxalic acid in H_2O	Strehlow (1962)
99	298	Review	Doussin and Monod (2013)

$K_{hydration}$	T /K	Comment	Reference
**100**	**298**	**Average value**	**This work**
α-Ketobutyric acid			
**1.703**	**298**	**NMR spectroscopy, 0.1 M in D_2O, pH = 0.5**	**Copper and Redfield (1975)**
**0.075**	**298**	**NMR spectroscopy, 0.1 M in D_2O, pH = 6**	**Copper and Redfield (1975)**
1.38	311	NMR spectroscopy, 0.5 M in D_2O, pH = 0.5	Kokesh (1976)
0.075	311	NMR spectroscopy, 0.55 M in D_2O, pH = 0.5	Kokesh (1976)
Oxaloacetic acid (2-Butanedioic acid, Oxobutanedioic acid)			
6.05	311	NMR spectroscopy, 1.4 M in H_2O, pH = 1.3 (6.2% enol form)	Kokesh (1976)
0.5	311	NMR spectroscopy, 1.4 M in H_2O, anion	Kokesh (1976)
0.06	311	NMR spectroscopy, 1.4 M in H_2O, pH = 6.89 (7.4% enol form)	Kokesh (1976)
**1.7**	**298**	**Polarography, diacid**	**Kozlowski and Zuman (1992)**
**0.06**	**298**	**Polarography, dianion**	**Kozlowski and Zuman (1992)**
2-Ketopentanoic acid (α-Ketovaleric acid, 2-Oxopentanoic acid)			
**1.6**	**298**	**Estimated value, stopped flow measurement, pH = 1.1 – 1.7**	**Buschmann et al. (1982)**
Dimethylpyruvic acid (α-Ketoisovaleric acid, 3-Methyl-2-oxobutanoic acid)			
1.703	298	NMR spectroscopy, 0.1 M in D_2O, pH = 0.5	Copper and Redfield (1975)
0.075	298	NMR spectroscopy, 0.1 M in D_2O, pH = 6	Copper and Redfield (1975)
1.786	298	NMR spectroscopy, in 10% D_2O, pH = 0, pH – dep. measurement	Lopalco et al. (2016)
0.037	298	NMR spectroscopy, in 10% D_2O, pH = 6, pH – dep. measurement	Lopalco et al. (2016)
**1.75**	**298**	**Dianion, average of 2 values at 298 K**	**This work**
**0.06**	**298**	**Diacid, average of 2 values at 298 K**	**This work**
Trimethylpyruvic acid (3,3-Dimethyl-2-oxobutanoic acid)			
**0.46**	**298**	**Polarography, c = 0.025 M, pH = 5**	**Wasa and Musha (1970)**
(dl-α-Keto-β-methylvaleric acid, 4-Methyl-2-oxopentanoic acid)			
1.5	298	NMR spectroscopy, 0.1 M in D_2O, pH = 0.5	Copper and Redfield (1975)
**0.285**	**298**	**NMR spectroscopy, in 10% D_2O, pH = 0, pH – dep. measurement**	**Lopalco et al. (2016)**
**0.006**	**298**	**NMR spectroscopy, in 10% D_2O, pH = 6, pH – dep. measurement**	**Lopalco et al. (2016)**
(α-Ketoisocaproic acid)			
**0.111**	**298**	**NMR spectroscopy, 0.1 M in D_2O, pH = 0.5**	**Copper and Redfield (1975)**
**>0.01**	**298**	**NMR spectroscopy, 0.1 M in D_2O, pH = 6**	**Copper and Redfield (1975)**
(α-Ketoglutaric acid)			
1.128	298	NMR spectroscopy, 0.1 M in D_2O, pH = 0.5	Copper and Redfield (1975)
0.064	298	NMR spectroscopy, 0.1 M in D_2O, pH = 6	Copper and Redfield (1975)
1.35	298	NMR spectroscopy	Jen and Knoche (1969)

$K_{hydration}$	T /K	Comment	Reference
0.03	298	NMR spectroscopy, pH = 5.3	Jen and Knoche (1969)
1.79	298	Polarography, diacid	Kozlowski and Zuman (1992)
0.26	298	Polarography, dianion	Kozlowski and Zuman (1992)
**1.4**	**298**	**Diacid, average of all values at 298 K**	**This work**
**0.12**	**298**	**Dianion, average of 2 values at 298 K**	**This work**
(α-Ketoadipinic acid)			
1.128	298	NMR spectroscopy, 0.1 M in D_2O, pH = 0.5	Copper and Redfield (1975)
**0.064**	**298**	**NMR spectroscopy, 0.1 M in D_2O, pH = 6**	**Copper and Redfield (1975)**
1.13	298	Estimated value, stopped flow measurement, pH = 1.1 – 1.6	Buschmann et al. (1982)
**1.129**	**298**	**Diacid, average of 2 values at 298 K**	**This work**
Acetopyruvic acid			
**4.4**	**301**	**NMR spectroscopy, pH = dep. measurement**	**Guthrie (1972)**

In the following paragraphs, the data compiled in Tables S3 and S4 are briefly discussed. It should be noted that individual data which have been already been compiled in former reviews are generally not repeated here.

Formaldehyde

The evaluation of the hydration constant of formaldehyde has been done several times (Bell, 1966; Ogata and Kawasaki, 1970; Doussin and Monod, 2013). Nevertheless, some studies were not included in these reviews. The values obtained $K_{hyd.}$ = 2190 at T = 293 K by Zavitsas et al. (1970), $K_{hyd.}$ = 2220 at T = 295 K by (Sutton and Downes, 1972), $K_{hyd.}$ = 2420 at T = 298 K by Lewis and Wolfenden (1973) and $K_{hyd.}$ = 2270 at T = 298 K by McDonald and Martin (1979) are slightly higher than the last recommended value $K_{hyd.}$ = 2000 at T = 298 K by Doussin and Monod (2013). Except the investigation of formaldehyde by Rivlin et al. (2015) with $K_{hyd.}$ = 2100 at T = 293 K no further recent measured value of the hydration equilibrium constant was added. Since there is no significant change in the recent reported values, the last recommendation suggested by Doussin and Monod (2013) should be applied.

Acetaldehyde

In contrast to formaldehyde, acetaldehyde has a significant smaller hydration equilibrium constant by a factor of 10^3, due to the electronic influence of the CH_3 group. The value recommended $K_{hyd.}$ = 1.43 at T = 298 K by Bell (1966) was suggested by a review of Tur'yan (2000) appears to be lower with $K_{hyd.}$ = 1.2 at T = 298 K. Further studies (Kurz, 1967; Lewis and Wolfenden, 1973; Sorensen and Jencks, 1987) which were not included in Tur'yan (2000) or Doussin and Monod (2013) indicated the same $K_{hyd.}$ value. The last recent review by Doussin and Monod (2013) suggested the use of the recommended value from Tur'yan (2000), which as well be the recommendation from the present work.

Propanal

Table S3 summarizes the given $K_{hyd.}$ values from Bell (1966) and the references therein, Greenzaid et al. (1967b); Lewis and Wolfenden (1973); Buschmann et al. (1982) as well as Doussin and Monod (2013) and the references therein. The recommended value $K_{hyd.}$ = 0.85 from Doussin and Monod (2013) refers to a slightly higher value based on studies from the 1980's. Overall, it is recommended to use the suggested value from Doussin and Monod (2013).

Butanal

The hydration of butanal ($K_{hyd.}$ = 0.43) was first revised by Bell (1966). Similar values have been determined by Greenzaid et al. (1967b); Lewis and Wolfenden (1973) compiled in Table S3. The recent evaluation by Doussin and Monod (2013) considering more recent investigations, which indicated higher values. The recommendation from Doussin and Monod (2013) as well as our recommendation can be given with $K_{hyd.}$ = 0.60.

Pivaldehyde

The investigation compiled by Greenzaid et al. (1967b); Lewis and Wolfenden (1973) as well as references included in Doussin and Monod (2013) supports the recommended value. A smaller value was found by Lienhard and Jencks (1966), but it has to be mentioned that the derived values tends to be too low in this study.

Methylglyoxal

A further hydration constant of the dicarbonyl compound was found in the literature, which was not considered by the evaluation in Doussin and Monod (2013). The $K_{hyd.}$ of methylglyoxal was reported with a value of 2700 (Wasa and Musha, 1970), 1279 (Montoya and Mellado, 1994) and 565 (Creighton et al., 1988). The new recommended value is suggested to be the average value of all three studies, with a derived $K_{hyd.}$ = 1512. Nevertheless, even with this value the majority (>99.9%) of the methylglyoxal is present in its gem-diol.

Glycolaldehyde

Bell (1966) recommend for glycolaldehyde a $K_{hyd.}$ = 25. The revision from Doussin and Monod (2013) considers more recent and higher values. The recommendation based on the reference in Doussin and Monod (2013) yield with $K_{hyd.}$ = 15.7. Our study follows this recommended value.

Acetone

In case of acetone, the reported values agree very well. Bell (1966) recommended a very low hydration equilibrium constant for acetone, which was also obtained by an investigation of Greenzaid et al. (1967b).

Diacetyl

The recommendation from Doussin and Monod (2013) for Diacetyl was based on more recent results from the references therein. A higher value $K_{hyd.}$ = 3.3 was earlier suggested by Bell (1966). Later on a smaller value was obtained by Lewis and Wolfenden (1973) ($K_{hyd.}$ = 0.244) and by Buschmann et al. (1982) ($K_{hyd.}$ = 2.1). Therefore, we also recommend the given value from Doussin and Monod (2013).

Further hydration constants for simple aldehydes and ketones have been evaluated in Doussin and Monod (2013). Since there are no further recent studies found in the literature, in many cases these available recommended values can be followed.

Glyoxylic acid

The study of Tur'yan (1998) compiled and reviewed the hydration equilibrium constants of glyoxylic acid as well as the deprotonated glyoxylate. The $K_{hyd.}$ values of this compound have been determined by UV/VIS spectroscopy (Strehlow, 1962; Ahrens, 1968; Sørensen et al., 1974), NMR spectroscopy (Cooper and Redfield, 1975; Rendina et al., 1984; Gunshore et al., 1985) and electrochemical methods (Kůta and Valenta, 1963; Fonds et al., 1969; Tur'yan, 1998). The obtained range of $K_{hyd.}$ >99 – 1700, results in an uncertainty of less than 1% for the appearance of the keto form of the protonated glyoxylic acid. Tur'yan (1998) recommended a value $K_{hyd.}$ = 1100 by measuring and evaluating the rate constant of the dehydration and recombination reaction of the glyoxylic acid. The variety of glyoxylate values is similar. The values reported in the literature change from $K_{hyd.}$ >15 – 260, results in 6.3% respectively 0.4% for the keto form of glyoxylate. The suggested recommended value $K_{hyd.}$ = 67 by Tur'yan (1998) indicates that only a small fraction of 1.5% of the glyoxylate is not hydrated, which is in good agreement with the directly reported fraction of 1.8% (Leitzke et al., 2001). Several pK_a values to describe the dissociation equilibrium of the keto form of glyoxylic acid at various ionic strength have been reported: pK_a = 1.72 (Leussing and Hanna, 1966), pK_a = 1.68 (Fonds et al., 1969) and pK_a = 1.70 – 1.75 (Tur'yan, 1998). In contrast the hydrated form of the glyoxylic acid (dihydroxyethanoic acid) has a slightly higher pK_a value: pK_a = 2.92 (Leussing and Hanna, 1966), pK_a = 3.06 (Fonds et al., 1969) and pK_a = 2.89 – 2.96 (Tur'yan, 1998). Further reported values do not distinguish between the keto form of the carbonyl group and the diol form (pK_a = 3.3 (Strehlow, 1962), pK_a = 3.46 (Öjelund et al., 1967).
Since the revision compiled by Tur'yan (1998) no recent determination of hydration constants of glyoxylic acid was found, with exception for the deprotonated glyoxylate from Leitzke et al. (2001). Nevertheless, Doussin and Monod (2013)and the present work follow the recommendation given by Tur'yan (1998).

Pyruvic acid

As in the previous case, many methods have been used to study the hydration equilibrium of pyruvic acid, such as pressure (Strehlow, 1962; Buschmann et al., 1982) or temperature jump relaxation (Ahrens, 1968), UV/VIS spectrometry (Knoche et al., 1985; Fischer and Warneck, 1991), NMR spectroscopy (Becker, 1964; Gold et al., 1964; Leussing and Stanfield, 1964;

Greenzaid et al., 1967b; Griffiths and Socrates, 1967; Öjelund et al., 1967; Socrates, 1967; Patting and Strehlow, 1969; Pocker et al., 1969; Hellström and Almqvist, 1970; Cooper and Redfield, 1975; Kokesh, 1976; Reed Harris et al., 2014; Lopalco et al., 2016; Rapf et al., 2017a; Rapf et al., 2017b), as well as electrochemical methods (Fonds et al., 1969; Fleury et al., 1977; Kozlowski and Zuman, 1987; Tur'yan, 1999). The values obtained from these studies are in a range from $K_{Hyd.1}$ = 0.6 – 3.2. The majority of the studies were undertaken at high pyruvic acid concentrations (> 2 mol L^{-1}), while the pH values were not adjusted or measured (cf. Table S4). Patting and Strehlow (1969) investigated the concentration dependency of the hydration equilibria from 0.14 – 10 mol L^{-1} at different temperatures. The results of these authors indicate that the hydration equilibrium constant is concentration-dependent. The value of the pyruvic acid for infinitive dilution at T = 298 K can be calculated with $K_{hyd.1}$ = 2.53 from their temperature-dependent studies. However, the evaluation of the hydration equilibrium constant $K_{hyd.1}$ is dependent on the used dissociation constant. In the literature, several pK_a values of the keto form respectively diol form of pyruvic acid can be found. Becker (1964) reported the pK_a values of the dehydrated and hydrate acid with pK_a = 2.07 and 3.6. Smaller values of have been reported by Hellström and Almqvist (1970) pK_a = 1.5 and 3.0, (Fischer et al., 1988) pK_a = 1.55 and 2.79. Fischer and Warneck (1991) reinvestigated the temperature dependent dissociation equilibria of pyruvic acid with the pK_a values 1.8 and 3.71. Lopalco et al. (2016) yield for the hydrated acid pK_a = 1.76 and in case of the dehydrated acid pK_a = 3.24. The use of the different dissociation constants can explain many of the uncertainties of the derived hydration constants. The pH-dependency of the equilibria was investigated by Pocker et al. (1969) using the pK_a values 2.18 (Strehlow, 1962) and 3.6 (Becker, 1964). Tur'yan (1999) reinvestigated the hydration equilibria by polarography.

In this study, many values of $K_{hyd.1}$ have been corrected by using the pK_a from Fischer and Warneck (1991), deriving a recommended value of $K_{hyd.1}$ = 2.4 at pH = 0 and T = 298 K (cf. Table S4). The more recent pH dependent measurements employed by Lopalco et al. (2016) and Rapf et al. (2017a) agree quite well with the recommended value from Tur'yan (1999), although Rapf et al. (2017a) used the pK_a values as in the study of Pocker et al. (1969) to describe the dissociation equilibria. In conclusion for pyruvic acid, the recommended value for the hydration equilibrium constant of pyruvic acid of $K_{hyd.1}$ = 1.4 given by Doussin and Monod (2013) appears too low when considering the recently published values (Lopalco et al., 2016; Rapf et al., 2017a). Our present review suggests to follow the recommendation of $K_{hyd.1}$ = 2.4 given by Tur'yan (1998).

Pyruvate

The variability of the hydration equilibrium constant $K_{hyd.2}$ of the pyruvate is reported in the literature in a range of $K_{hyd.2}$ < 0.1 at T = 298 K. The present study recommends a $K_{hyd.}$ = 0.08 of pyruvate, as an average value from the data at T = 298 K in Table S4. This value is slightly higher than recommendation by Doussin and Monod (2013).

Mesoxalic acid

In case of the mesoxalic acid only the fully protonated dicarboxylic acid equilibrium constant was reported by Strehlow (1962) ($K_{hyd.}$ = 100) and by Le Henaff (1968) ($K_{hyd.}$ = 99). The derived recommended value $K_{Hyd.}$ = 100 suggested from this present

review is very close to the value of $K_{hyd.}$ = 99 suggested by Doussin and Monod (2013). Unfortunately, there are no further data reported concerning the pH dependency of mesoxalic acid.

Other α-oxocarboxylic acids

In case of other α-oxocarboxylic acids a few more hydration equilibrium constants have been reported in the literature by Jen and Knoche (1969); Wasa and Musha (1970); Guthrie (1972); Cooper and Redfield (1975); Kokesh (1976); Buschmann et al. (1982); Kozlowski and Zuman (1992); Lopalco et al. (2016) and compiled in Kerber and Fernando (2010). The values for the compounds oxaloacetic acid, α-ketopentanoic acid, dimethylpyruvic acid, trimethylpyruvic acid, α-keto-β-methylvaleric acid, α-ketoisocaproic acid, α-ketoglutaric acid, α-ketoadipinic acid and acetopyruvic acid were taken from the aforementioned references and compiled in (Table S4). Jen and Knoche (1969); Guthrie (1972); Kokesh (1976); Buschmann et al. (1982); Kozlowski and Zuman (1992) studied an even more complex system and determined the hydration and enolization equilibria. In general, the pH dependent behaviour of the apparent hydration constant $K_{app.}$, similar to pyruvic acid, is expected because of the connected equilibria. The α-oxocarboxylic acids were found to exist in equilibrium with the hydrate (gem-diol). The equilibrium position favours the hydrated α-oxocarboxylic acids under acidic conditions, but at neutral pH values the dehydrated keto-form predominates, depending on the pK_a values. With the exception of mesoxalic acid and acetopyruvic acid the reported values of $K_{hyd.}$ are smaller than < 1.8 under acidic conditions and for neutral conditions they are even smaller (cf. Table S4).

2-imidazol-carboxaldehyde

In addition to the compounds discussed up to here, a similar pH dependency of the hydration constant $K_{hyd.}$ was reported by Ackendorf et al. (2017) for 2-imidazol-carboxaldehyde or 2-IC (cf. Table S3). Under acidic conditions, the photochemical active side-chain aldehyde group undergoes the equilibrium reaction with water and forms the gem-diol while the imidazole ring will be fully protonated. In general, 2-IC behaves as a double-basic acid.

Regarding further studies of multifunctional carbonyl compounds, based on the findings discussed here, future studies should consider the complex equilibria which can result in complex dependencies on hydration of carbonyl compounds on acidity.

Computational chemistry

For simple aldehydes and ketones the comparison in Doussin and Monod (2013) of calculated towards measured hydration constants, leads to reasonable derived values for T = 298 K (Guthrie, 2000; Hilal et al., 2005; Gomez-Bombarelli et al., 2009; Raventos-Duran et al., 2010). Nevertheless, for molecules that are more complex the values obtained by calculation methods scatter in a broader range around the determined equilibrium constant, partly with a poor level of agreement. Apparently, there is quite some potential for improvement for numerical predictions of hydration constants, especially for more complex species.

Apparently, there is quite some potential for improvement for numerical predictions of hydration constants, especially for more complex species.

Table S5. Compiled kinetic data (k_{OH}(298 K) in M^{-1} s^{-1})) of OH radical reactions with dissociating organic compounds.

Reactant	SMILES	k_{298K} [M^{-1} s^{-1}]	Reference
**Monocarboxylic acids (undissociated and dissociated)**			
Formic acid	C(=O)O	1.30E+08	Buxton et al. (1988)
Formate	C(=O)[O-]	3.20E+09	Buxton et al. (1988); Elliot and Simsons (1984)
Acetic acid	CC(=O)O	1.70E+07	Chin and Wine (1994)[c]
Acetate	CC(=O)[O-]	7.30E+07	Chin and Wine (1994)[c]
Propionic acid	CCC(=O)O	3.20E+08	Ervens et al. (2003)
Propionate	CCC(=O)[O-]	7.20E+08	Ervens et al. (2003)
Butyric acid	CCCC(=O)O	2.20E+09	Scholes and Willson (1967)
Butyrate	CCCC(=O)[O-]	2.00E+09	Anbar et al. (1966)
iso-Valeric acid	CC(C)CC(=O)O	1.40E+09	Merz and Waters (1949)
iso-Valerate	CC(C)CC(=O)[O-]	2.40E+09	Anbar et al. (1966)
Pivalic acid	CC(C)(C)C(=O)O	6.50E+08	Nauser and Bühler (1994)
Pivalate	CC(C)(C)C(=O)[O-]	1.10E+09	Average of Nauser and Bühler (1994) and Anbar et al. (1966)
cis-Pinonic acid	CC(=O)C1CC(C1(C)C)CC(=O)O	2.70E+09	Otto et al. (2018)
cis-Pinonate	CC(=O)C1CC(C1(C)C)CC(=O)[O-]	2.50E+09	Otto et al. (2018)
**Unsaturated monocarboxylic acids (undissociated and dissociated)**			
Acrylic acid	C=CC(=O)O	1.50E+09	Walling and El-Taliawi (1973)[a]
Acrylate	C=CC(=O)[O-]	5.70E+09	Average of Kumar et al. (1988)[a] and Maruthamuthu (1980)[a]
Crotonic acid	CC=CC(=O)O	2.90E+09	Walling and El-Taliawi (1973)[b]
Crotonate	CC=CC(=O)[O-]	5.00E+09	Lilie and Henglein (1970) Maruthamuthu and Dhandavel (1980)[b]
Methacrylic acid	C=C(C)C(=O)O	1.20E+10	Schöne et al. (2014)
Methacrylate	C=C(C)C(=O)[O-]	1.20E+10	Schöne et al. (2014)
Methylcrotonic acid	CC(C)=CC(=O)O	9.00E+09	Kumar and Rao (1991)[a]
Methylcrotonate	CC(C)=CC(=O)[O-]	5.90E+09	Average of different measurements of Kumar and Rao (1991)[a]
**Dicarboxylic acids (undissociated and dissociated)**			
Oxalic acid	OC(=O)C(=O)O	1.40E+06	Sehested et al. (1971)
Oxalate monoanion	OC(=O)C(=O)[O-]	1.90E+08	Ervens et al. (2003)
Oxalate dianion	[O-]C(=O)C(=O)[O-]	1.60E+08	Ervens et al. (2003)
Malonic acid	OC(=O)CC(=O)O	2.00E+07	Buxton et al. (1988)
Malonate monoanion	OC(=O)CC(=O)[O-]	6.00E+07	Ervens et al. (2003)
Malonate dianion	[O-]C(=O)CC(=O)[O-]	2.70E+08	Average of Logan (1989) and Adams et al. (1965)

Reactant	SMILES	k_{298K} [M^{-1} s^{-1}]	Reference
Succinic acid	OC(=O)CCC(=O)O	1.10E+08	Ervens et al. (2003)
Succinate dianion	[O-]C(=O)CCC(=O)[O-]	5.00E+08	Ervens et al. (2003)
Glutaric acid	OC(=O)CCCC(=O)O	2.00E+08	Herrmann (2003)
Glutarate dianion	[O-]C(=O)CCCC(=O)[O-]	1.00E+09	Herrmann (2003)
Adipic acid	OC(=O)CCCCC(=O)O	1.70E+09	Herrmann (2003)
Adipate dianion	[O-]C(=O)CCCCC(=O)[O-]	2.40E+09	Herrmann (2003)
Pimelic acid	OC(=O)CCCCCC(=O)O	2.40E+09	Herrmann (2003)
Pimelate dianion	[O-]C(=O)CCCCCC(=O)[O-]	2.90E+09	Herrmann (2003)
Suberic acid	OC(=O)CCCCCCC(=O)O	5.00E+09	Hesper (2003)
Suberate dianion	[O-]C(=O)CCCCCCC(=O)[O-]	5.80E+09	Hesper (2003)
(+)-Camphoric acid	CC1(C(CCC1(C)C(=O)O)C(=O)O)C	2.00E+09	Otto et al. (2018)
(+)-Camphoriate monoanion	CC1(C(CCC1(C)C(=O)O)C(=O)[O-])C	2.60E+09	Otto et al. (2018)
(+)-Camphoriate monoanion	CC1(C(CCC1(C)C(=O)[O-])C(=O)[O-])C	2.60E+09	Otto et al. (2018)
**Aliphatic polyfunctional monocarboxylic acids (undissociated and dissociated)**			
Glycolic acid	OCC(=O)O	6.00E+08	Buxton et al. (1988)
Glycolate	OCC(=O)[O-]	1.20E+09	Logan (1989)[a]
Hydrated glyoxylic acid	OC(O)C(=O)O	3.60E+08	Ervens et al. (2003)
Hydrated glyoxylate	OC(O)C(=O)[O-]	2.60E+09	Ervens et al. (2003)
Lactic acid	CC(O)C(=O)O	5.24E+08	Martin et al. (2009)
Lactate	CC(O)C(=O)[O-]	7.77E+08	Martin et al. (2009)
Pyruvic acid	CC(=O)C(=O)O	1.20E+08	Ervens et al. (2003)
Pyruvate	CC(=O)C(=O)[O-]	7.00E+08	Ervens et al. (2003)
**Aliphatic polyfunctional dicarboxylic acids (undissociated and dissociated)**			
Tartronic acid	OC(=O)C(O)C(=O)O	1.70E+08	Schuchmann et al. (1995)
Tartronate monoanion	OC(=O)C(O)C(=O)[O-]	3.60E+08	Schuchmann et al. (1995)
Tartronate dianion	[O-]C(=O)C(O)C(=O)[O-]	4.40E+08	Schuchmann et al. (1995)
Mesoxalic acid	OC(=O)C(=O)C(=O)O	1.80E+08	Gligorovski et al. (2009)
Mesoxalate monoanion	OC(=O)C(=O)C(=O)[O-]	5.70E+07	Schuchmann et al. (1991)
Mesoxalate dianion	[O-]C(=O)C(=O)C(=O)[O-]	1.00E+08	Schuchmann et al. (1991)
Malic acid	OC(=O)CC(O)C(=O)O	3.60E+08	Gligorovski et al. (2009)
Malate monoanion	OC(=O)CC(O)C(=O)[O-]	9.70E+08	Gligorovski et al. (2009)
Malate dianion	[O-]C(=O)CC(O)C(=O)[O-]	8.50E+08	Gligorovski et al. (2009)
Tartaric acid	OC(=O)C(O)C(O)C(=O)O	1.10E+09	Average of Scholes and Willson (1967)[b] and Moore et al. (1979)[a]
Tartrate dianion	[O-]C(=O)C(O)C(O)C(=O)[O-]	1.00E+08	Average of Kraljic (1967)[a,b] and Logan (1989)
Citric acid	OC(=O)CC(O)(C(=O)O)CC(=O)O	5.00E+07	Adams et al. (1965)[b]

Reactant	SMILES	k_{298K} $[M^{-1} s^{-1}]$	Reference
Citrate	[O-]C(=O)CC(O)(C(=O)[O-])CC(=O)[O-]	1.50E+08	Zepp et al. (1992)[a]
**Phenols and other aromatic compounds with alcohol functionalities**			
Phenol	c1ccccc1O	1.01E+10	Chen and Schuler (1993); partial k @ ortho-/meta-/para-position: $2.37 \cdot 10^9/1.3 \cdot 10^8/3.56 \cdot 10^9$ M^{-1} s^{-1}
Phenolate	c1ccccc1[O-]	9.60E+09	Bonin et al. (2007)
2-Nitrophenol	Oc1ccccc1N(=O)=O	9.20E+09	Savel'eva et al. (1972)[d]
2-Nitrophenolate	[O-]c1ccccc1N(=O)=O	9.20E+09	Savel'eva et al. (1972)[b]
3-Nitrophenol	c1ccc(O)cc1N(=O)=O	4.10E+09	Biswal et al. (2013)
3-Nitrophenolate	c1ccc([O-])cc1N(=O)=O	8.70E+09	Biswal et al. (2013)
2,4-Nitrophenol	c1cc(O)ccc1N(=O)=O	1.76E+09	Albinet et al. (2010)
2,4-Nitrophenolate	c1cc([O-])ccc1N(=O)=O	2.33E+09	Albinet et al. (2010)
2-Hydroxybenzaldehyde	c1(O)ccccc1C=O	5.20E+09	Geeta et al. (2001)
2-Hydroxybenzaldehyde, conjugated base	c1([O-])ccccc1C=O	5.20E+09	Savel'eva et al. (1972)[b]
4-Hydroxybenzaldehyde	c1cc(O)ccc1C=O	1.21E+10	Geeta et al. (2001)
4-Hydroxybenzaldehyde, conjugated base	c1cc([O-])ccc1C=O	1.00E+10	Savel'eva et al. (1972)[2]
**Aromatic mono-/dicarboxylic acids (undissociated and dissociated)**			
Benzoic acid	c1ccccc1C(=O)O	3.10E+09	Average of Wander et al. (1968)[b] and Ashton et al. (1995)[a]
Benzoate	c1ccccc1C(=O)[O-]	5.90E+09	Buxton et al. (1988)
2-Hydroxybenzoic acid	c1(O)ccccc1C(=O)O	2.20E+10	Buxton et al. (1988)
2-Hydroxybenzoate	c1(O)ccccc1C(=O)[O-]	1.60E+10	Buxton et al. (1988)
4-Hydroxybenzoic acid	c1cc(O)ccc1C(=O)O	7.70E+09	Average of Neta and Dorfman (1968)[a]; Anderson et al. (1987)[a]; and Shetiya et al. (1976)[a]
4-Hydroxybenzoate	c1cc(O)ccc1C(=O)[O-]	8.50E+09	Buxton et al. (1988)
Gallic acid	c1=c(c=c(c(=c1O)O)O)C(=O)O	6.40E+09	Dwibedy et al. (1999)
Gallate	c1=c(c=c(c(=c1O)O)O)C(=O)[O-]	1.10E+10	Dwibedy et al. (1999)
Salicylic acid	c1=cc=c(c(=c1)C(=O)O)O	2.20E+10	Buxton et al. (1988)
Salicylate anion	c1=cc=c(c(=c1)C(=O)[O-])O	1.60E+10	Buxton et al. (1988)
**Imidazoles**			
Imidazole-2-carboxaldehyde (pH=0)	C1=C[NH+]=C(N1)C(O)O	1.80E+09	Felber et al. (2019)
Imidazole-2-carboxaldehyde (pH=9.1)	C1=CN=C(N1)C=O	4.40E+09	Felber et al. (2019)
1-Methylimidazole (pH=2)	CN1C=C[NH+]=C1	2.30E+09	Felber et al. (2019)

Reactant	SMILES	k_{298K} [M^{-1} s^{-1}]	Reference
1-Methylimidazole (pH=9.1)	CN1C=CN=C1	6.10E+09	Felber et al. (2019)
2-Methylimidazole (pH=2)	CC1=[NH+]C=CN1	3.90E+09	Felber et al. (2019)
2-Methylimidazole (pH=9.2)	CC1=NC=CN1	5.70E+09	Felber et al. (2019)
4-Methylimidazole (pH=2)	CC1=C[NH+]=CN1	3.20E+09	Felber et al. (2019)
4-Methylimidazole (pH=9.1)	CC1=CN=CN1	6.90E+09	Felber et al. (2019)
1-Ethylimidazole (pH=2)	CCN1C=C[NH+]=C1	2.60E+09	Felber et al. (2019)
1-Ethylimidazole (pH=9.1)	CCN1C=CN=C1	5.70E+09	Felber et al. (2019)
2-Ethylimidazole (pH=2)	CCC1=[NH+]C=CN1	3.90E+09	Felber et al. (2019)
2-Ethylimidazole (pH=9.2)	CCC1=NC=CN1	6.40E+09	Felber et al. (2019)

Remarks: References taken from: [a]NIST database: Ross et al. (1998); [b]Buxton et al. (1988); [c]Warneck (2005); [d]Barzaghi and Herrmann (2004)

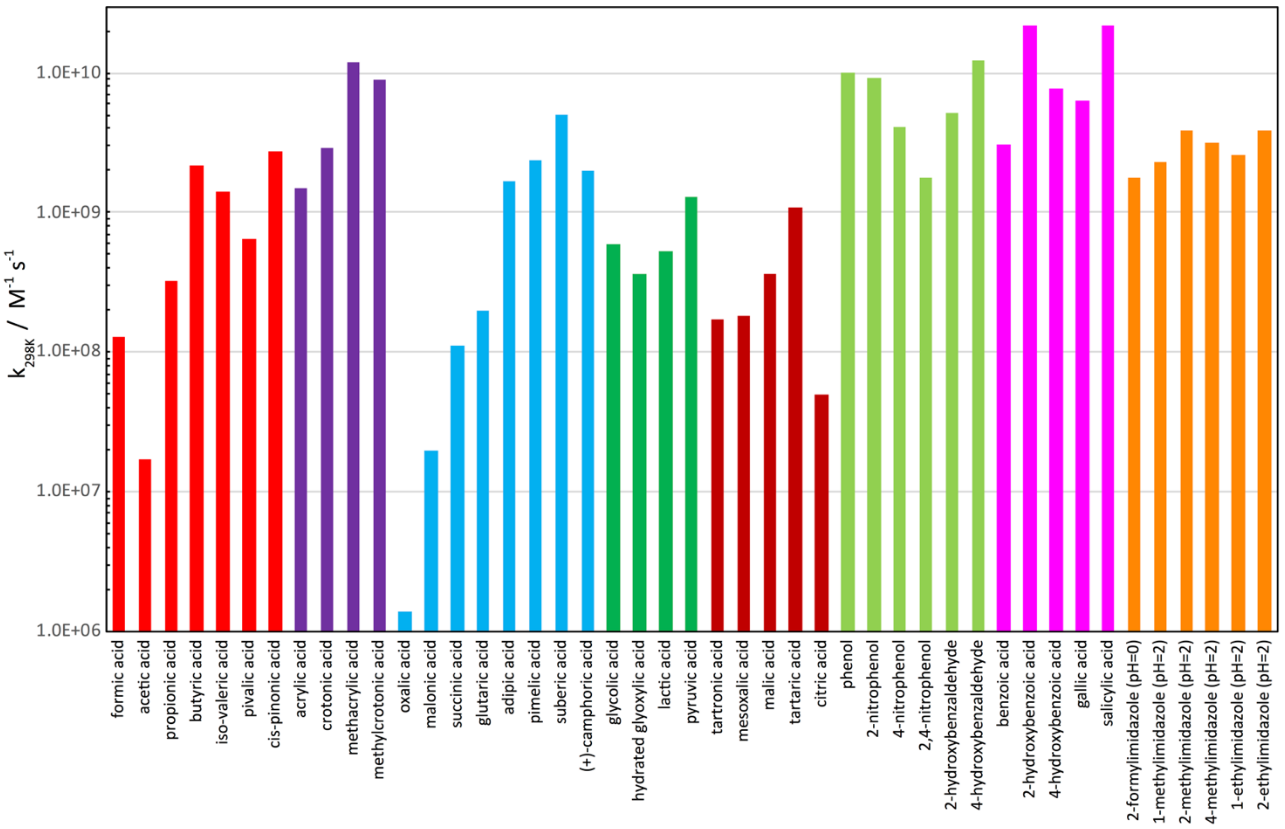

Figure S3. Schematic of second-order OH reaction rate constants (k_{OH}(298 K) in M^{-1} s^{-1}) of protonated organic compounds included in Table S5.

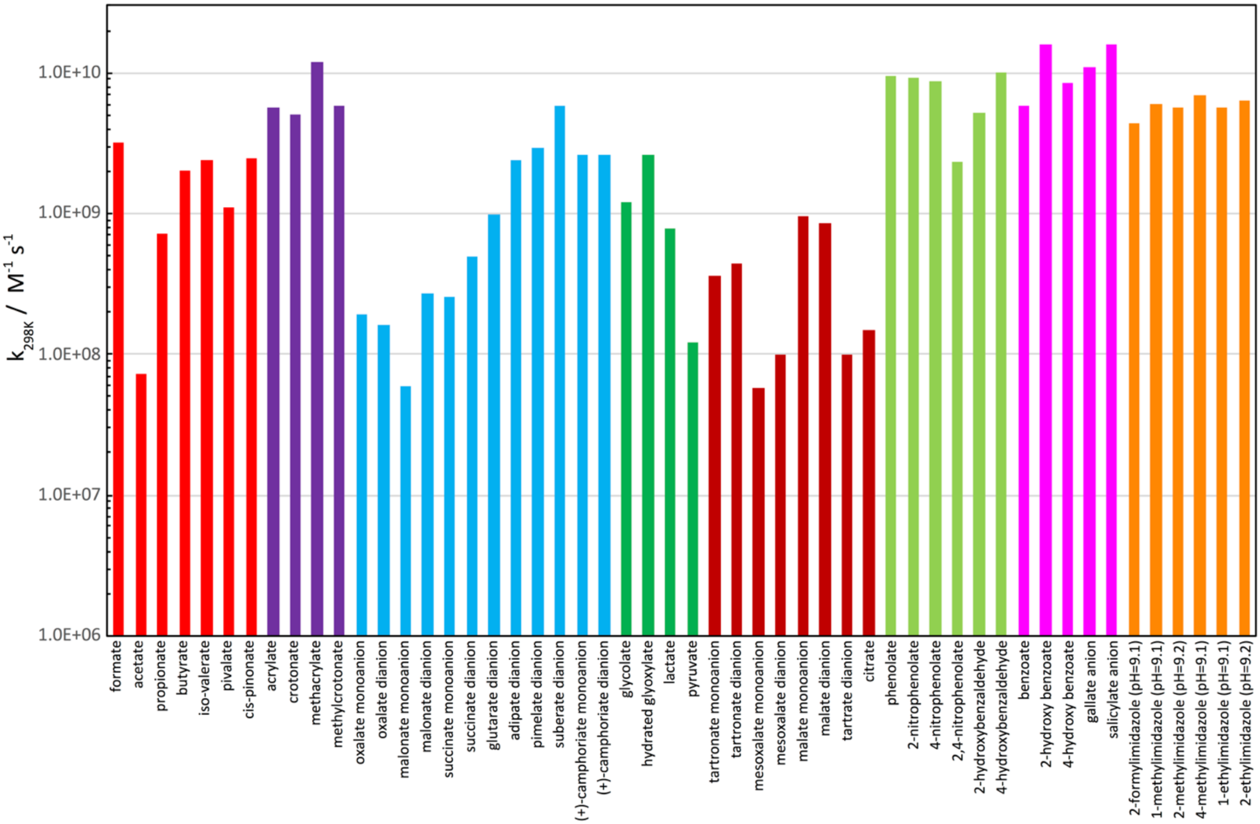

Figure S4. Schematic of the second-order OH reaction rate constants (k_{OH}(298 K) in M^{-1} s^{-1}) of deprotonated organic compounds included in Table S5.

Table S6. Compiled kinetic data (k_{NO3}(298 K) in M^{-1} s^{-1})) of NO_3 radical reactions with dissociating organic compounds.

**Reactant**	**SMILES**	**k_{298K} [M^{-1} s^{-1}]**	**Reference**
Monocarboxylic acids (undissociated and dissociated)			
Formic acid	C(=O)O	3.80E+05	Exner et al. (1994)
Formate	C(=O)[O-]	5.10E+07	Exner et al. (1994)
Acetic acid	CC(=O)O	1.30E+04	Exner et al. (1994)
Acetate	CC(=O)[O-]	2.90E+06	Exner et al. (1994)
cis-Pinonic acid	CC(=O)C1CC(C1(C)C)CC(=O)O	1.51E+06	Otto et al. (2018)
cis-Pinonate	CC(=O)C1CC(C1(C)C)CC(=O)[O-]	1.58E+07	Otto et al. (2018)
Unsaturated monocarboxylic acids (undissociated and dissociated)			
Acrylic acid	C=CC(=O)O	6.90E+06	Schöne et al. (2014)
Acrylate	C=CC(=O)[O-]	4.40E+07	Schöne et al. (2014)
Methacrylic acid	C=C(C)C(=O)O	9.20E+07	Schöne et al. (2014)
Methacrylate	C=C(C)C(=O)[O-]	1.70E+08	Schöne et al. (2014)
Dicarboxylic acids (undissociated and dissociated)			
Oxalic acid	OC(=O)C(=O)O	2.40E+04	Yang et al. (2004)
Oxalate monoanion	OC(=O)C(=O)[O-]	6.10E+07	Avg. of Yang et al. (2004) and de Sémainville et al. (2010) with E_a/R of Raabe (1996)
Oxalate dianion	[O-]C(=O)C(=O)[O-]	2.20E+08	Average of Yang et al. (2004) and de Sémainville et al. (2010) with E_a/R of de Sémainville et al. (2010)
Malonic acid	OC(=O)CC(=O)O	5.10E+04	de Sémainville et al. (2010)
Malonate monoanion	OC(=O)CC(=O)[O-]	5.60E+06	de Sémainville et al. (2010)
Malonate dianion	[O-]C(=O)CC(=O)[O-]	2.30E+07	de Sémainville et al. (2010)
Succinic acid	OC(=O)CCC(=O)O	5.00E+03	de Sémainville et al. (2010)
Succinate monoanion	OC(=O)CCC(=O)[O-]	1.10E+07	de Sémainville et al. (2010)
Succinate dianion	[O-]C(=O)CCC(=O)[O-]	1.80E+07	de Sémainville et al. (2010)
(+)-Camphoric acid	CC1(C(CCC1(C)C(=O)O)C(=O)O)C	8.80E+05	Otto et al. (2018)
(+)-Camphoriate monoanion	CC1(C(CCC1(C)C(=O)O)C(=O)[O-])C	4.31E+07	Otto et al. (2018)
(+)-Camphoriate monoanion	CC1(C(CCC1(C)C(=O)[O-])C(=O)[O-])C	5.93E+07	Otto et al. (2018)
Substituted mono-/dicarboxylic acids (undissociated and dissociated)			
Glycolic acid	OCC(=O)O	9.10E+05	de Semainville et al. (2007)
Glycolate	OCC(=O)[O-]	1.00E+07	de Semainville et al. (2007)
Lactic acid	CC(O)C(=O)O	2.10E+06	de Semainville et al. (2007)
Lactate	CC(O)C(=O)[O-]	1.00E+07	de Semainville et al. (2007)
Pyruvic acid	CC(=O)C(=O)O	2.40E+06	de Semainville et al. (2007)

Reactant	SMILES	k_{298K} [M^{-1} s^{-1}]	Reference
Pyruvate	CC(=O)C(=O)[O-]	1.90E+07	de Semainville et al. (2007)
Mesoxalic acid	OC(=O)C(=O)C(=O)O	1.70E+06	de Semainville et al. (2007)
Mesoxalate monoanion	OC(=O)C(=O)C(=O)[O-]	2.30E+07	de Semainville et al. (2007)
Mesoxalate dianion	[O-]C(=O)C(=O)C(=O)[O-]	4.90E+07	de Semainville et al. (2007)
Aromatic acids (undissociated and dissociated)			
4-Hydroxybenzoic acid	c1cc(O)ccc1C(=O)O	1.60E+09	Weller et al. (2010)
4-Hydroxybenzoate	c1cc(O)ccc1C(=O)[O-]	6.00E+09	Anderson et al. (1987)
4-Methoxybenzoic acid	c1cc(OC)ccc1C(=O)O	6.90E+08	Umschlag et al. (2002)
4-Methoxybenzoate	c1cc(OC)ccc1C(=O)[O-]	8.00E+09	O'Neill et al. (1977)

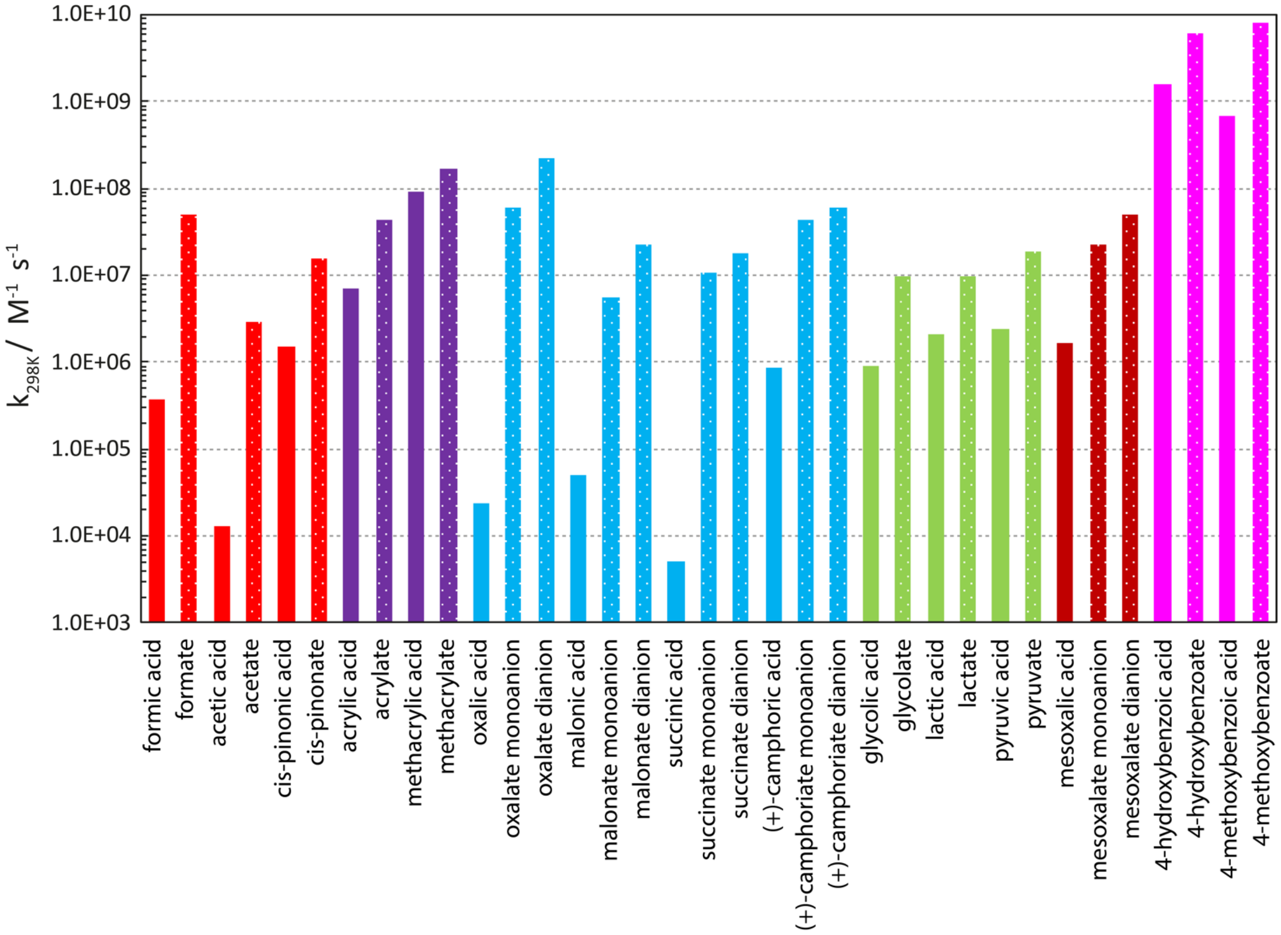

Figure S5. Schematic of second-order NO_3 reaction rate constants (k_{NO3}(298 K) in M^{-1} s^{-1}) of deprotonated and protonated organic compounds included in Table S6.

Table S7. Compiled kinetic data (k_{O3}(298 K) in $M^{-1} s^{-1}$)) of O_3 reactions with dissociating organic compounds.

Reactant	k_{298K} $M^{-1} s^{-1}$	Formula	Remarks	Reference
**Saturated aliphatic mono-carboxylic acids (undissociated and dissociated)**				
Glycolic acid	0.055	C(O)C(=O)O	pH = 1.0	Schöne and Herrmann (2014)
Glycolate	0.71	C(O)C(=O)[O-]	pH = 7.0	Schöne and Herrmann (2014)
Glyoxylic acid	0.14	O=CC(=O)O	pH = 1.0	Schöne and Herrmann (2014)
Glyoxylate	2.3	O=C(C(=O)[O-]	pH = 7.0	Schöne and Herrmann (2014)
Pyruvic acid	0.13	CC(=O)C(=O)O	pH = 1.0	Schöne and Herrmann (2014)
Pyruvate	0.98	CC(=O)C(=O)[O-]	pH = 7.0	Schöne and Herrmann (2014)
**Unsaturated aliphatic mono/di-carboxylic acids (undissociated and dissociated)**				
Acrylic acid	2.80E+04	C=CC(=O)O	pH = 2.0	Leitzke and von Sonntag (2009)
Acrylate	1.60E+05	C=CC(=O)[O-]	pH = 7.0	Leitzke and von Sonntag (2009)
Methacrylic acid	1.50E+05	C=CC(C)(=O)O	pH = 2.0	Leitzke and von Sonntag (2009)
Methacrylate	3.70E+06	C=CC(C)(=O)[O-]	pH = 7.0	Leitzke and von Sonntag (2009)
Maleic acid	1.40E+03	O=C(O)C=CC(=O)O		Leitzke and von Sonntag (2009)
Maleic acid monoanion	4.20E+03	O=C(O)C=CC(=O)[O-]		Leitzke and von Sonntag (2009)
Maleic acid dianion	≈ 7.0E+03	OC([O-])C=CC(=O)[O-]		Leitzke and von Sonntag (2009)
Fumaric acid	8.50E+03	O=C(O)C=CC(=O)O		Leitzke and von Sonntag (2009)
Fumaric acid dianion	1.68E+05	OC([O-])C=CC(=O)[O-]	pH = 10	Average of Leitzke and von Sonntag (2009) and King et al. (2008)
cis-cis-Muconic acid monoanion	4.00E+04<br>2.65E+04	O=C(O)C=CC=CC(=O)[O-]	pH = 3.1<br>pH = 6.0	Mvula and von Sonntag (2003)<br>Leitzke and von Sonntag (2009)
cis-trans-Muconic acid	1.40E+04<br>2.50E+05	O=C(O)C=CC=CC(=O)[O-]	pH = 3.0<br>pH = 7.0	Ramseier and Gunten (2009)<br>Ramseier and Gunten (2009)
trans-trans-Muconic acid monoanion	1.60E+04	O=C(O)C=CC=CC(=O)[O-]	pH = 3.0	Beltran et al. (2006)
trans-trans-Muconic acid dianion	1.40E+05	OC([O-])C=CC=CC(=O)[O-]	pH = 7.0	Beltran et al. (2006)
Limononic acid	2.60E+04	CC(=C)C(CCC(C)=O)CC(=O)O	pH = 2	Witkowski et al. (2018a); Witkowski et al. (2018b)
Limononic acid mono-anion	5.70E+04	CC(=C)C(CCC(C)=O)CC(=O)[O-]	pH = 7	Witkowski et al. (2018a); Witkowski et al. (2018b)
β-Caryophyllonic acid	4.80E+05	CC(=O)CCC1C(CC1(C)C)C(=C)C CC(=O)O	pH = 2	Witkowski et al. (2019)
β-Caryophyllonic acid mono-anion	6.00E+05	CC(=O)CCC1C(CC1(C)C)C(=C)C CC(=O)[O-]	pH = 8	Witkowski et al. (2019)
Limonic acid	2.30E+04	OC(=O)CCC(CC(=O)O)C(=C)C	pH = 2	Witkowski et al. (2018a)

Limonic acid mono-anion	4.60E+04	[O-]C(=O)CCC(CC(=O)O)C(=C)C	pH = 7	Witkowski et al. (2018a)
7-Hydroxylimononic acid	2.90E+04	O=C(CCC(CC(=O)O)C(=C)C)CO	pH = 2	Witkowski et al. (2018a)
7-Hydroxylimononic acid mono-anion	5.30E+04	[O-]C(CCC(CC(=O)O)C(=C)C)CO	pH = 7	Witkowski et al. (2018a)
**Aromatic organic compounds (undissociated and dissociated)**				
Phenol	8.67E+02	c1ccccc1O	pH = 2.0	Poznyak and Vivero (2005)
	1.17E+03		pH = 7.0	Poznyak and Vivero (2005)
Phenolate	1.4E+09	c1ccccc1[O-]		Hoigné and Bader (1983)
Tyrosol	3.00E+03	c1=cc(=cc=c1CCO)O	pH = 2.0	Beltrán et al. (2000)
	2.00E+05		pH = 6.3	Beltrán et al. (2000)
	6.80E+07		pH = 9.0	Beltrán et al. (2000)
4-*n*-Nonylphenol	3.80E+04	CCCCCCCCCc1=cc=c(c=c1)O		Deborde et al. (2005)
	3.90E+04			Ning et al. (2007)
4-*n*-Nonylphenol anion	6.80E+09	CCCCCCCCCc1=cc=c(c=c1)[O-]		Deborde et al. (2005)
**Aromatic acids (undissociated and dissociated)**				
p-Hydroxybenzoic acid	2.0E+02	c1=cc(=cc=c1C(=O)O)O	pH = 2.0	Beltrán et al. (2000)
	1.8E+05		pH = 6.3	Beltrán et al. (2000)
	6.4E+07		pH = 9.0	Beltrán et al. (2000)
Gallic acid	9.7E+04	c1=c(c=c(c(=c1O)O)O)C(=O)O	pH = 2.0	Beltrán et al. (2000)
	4.7E+05		pH = 6.3	Beltrán et al. (2000)
Cinnamic acid	1.0E+05	c1=cc=c(c=c1)C=CC(=O)O		Jans (1996)
Cinnamic acid monoanion	1.2E+06	c1=cc=c(c=c1)C=CC(=O)[O-]		Jans (1996)
	3.8E+05			Leitzke et al. (2001)
4-methoxy Cinnamic acid	1.3E+05	COc1=cc=c(c=c1)C=CC(=O)O		Leitzke et al. (2001)
4-methoxy Cinnamic acid monoanion	6.8E+05	COc1=cc=c(c=c1)C=CC(=O)[O-]		Leitzke et al. (2001)
3-methoxy-4-hydroxy Cinnamic acid	1.1E+06	COc1=c(c=cc(=c1)C=CC(=O)O)O		Jans (1996)
3-methoxy-4-hydroxy Cinnamic acid monoanion	7.9E+06	COc1=c(c=cc(=c1)C=CC(=O)([O-]))O		Jans (1996)
3,4-dihydroxy Cinnamic acid	2.0E+06	c1=cc(=c(c=c1C=CC(=O)O)O)O		Jans (1996)
3,4-dihydroxy Cinnamic acid monoanion	1.2E+07	c1=cc(=c(c=c1C=CC(=O)([O-]))O)O		Jans (1996)

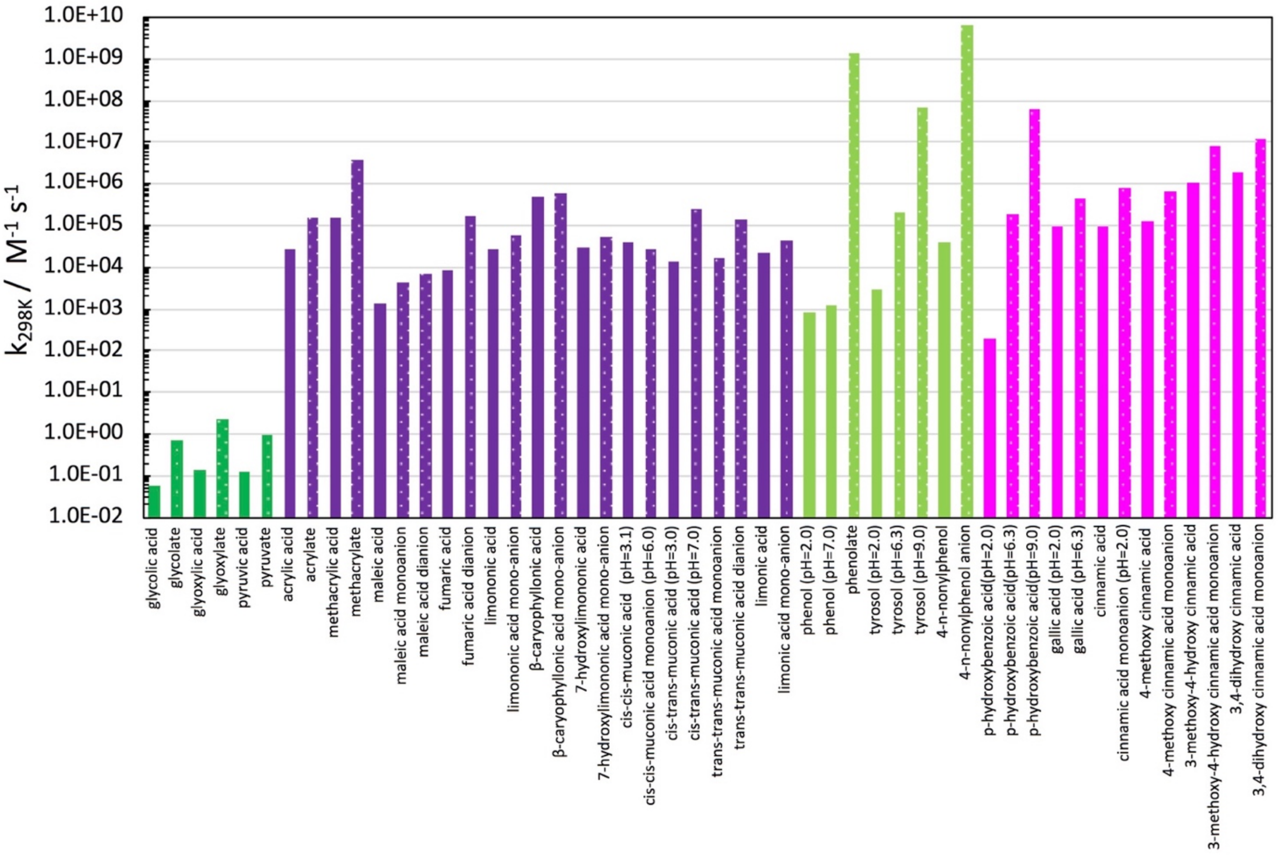

Figure S6. Schematic of second-order O_3 reaction rate constants (k_{O3}(298 K) in M^{-1} s^{-1}) of deprotonated and protonated organic compounds included in Table S7.

References

Ackendorf, J. M., Ippolito, M. G., and Galloway, M. M.: pH Dependence of the Imidazole-2-carboxaldehyde Hydration Equilibrium: Implications for Atmospheric Light Absorbance, Environ. Sci. Technol., 4, 551-555, https://doi.org/10.1021/acs.estlett.7b00486, 2017.

Adams, G. E., Boag, J. W., Currant, J., and Michael, B.: Absolute rate constants for the reaction of the hydroxyl radical with organic compounds, in: Pulse Radiolysis, edited by: Ebert, M., Keene, J. P., Swallow, A. J., and Baxendale, J. H., Academic Press, New York, 131-143, 1965.

Ahrens, M. L., and Strehlow, H.: Acid catalyzed hydration of acetaldehyde, Discuss. Faraday Soc., 39, 112-120, https://doi.org/10.1039/DF9653900112, 1965.

Ahrens, M. L.: Hydration Kinetics of Carbonyl Compounds, Ber. Bunsenges. Phys. Chem., 72, 691-696, https://doi.org/10.1002/bbpc.19680720606, 1968.

Albinet, A., Minero, C., and Vione, D.: Phototransformation processes of 2,4-dinitrophenol, relevant to atmospheric water droplets, Chemosphere, 80, 753-758, https://doi.org/10.1016/j.chemosphere.2010.05.016, 2010.

Anbar, M., Meyerstein, D., and Neta, P.: Reactivity of aliphatic compounds towards hydroxyl radicals, J. Chem. Soc. B, 742-747, https://doi.org/10.1039/j29660000742, 1966.

Anderson, R. F., Patel, K. B., and Stratford, M. R. L.: Radical spectra and product distribution following electrophilic attack by the $OH^{\cdot}$ radical on 4-hydroxybenzoic acid and subsequent oxidation, J. Chem. Soc., Faraday Trans., 83, 3177-3187, https://doi.org/10.1039/f19878303177, 1987.

Ashton, L., Buxton, G. V., and Stuart, C. R.: Temperature dependence of the rate of reaction of OH with some aromatic compounds in aqueous solution. Evidence for the formation of a π-complex intermediate?, J. Chem. Soc., Faraday Trans., 91, 1631-1633, https://doi.org/10.1039/ft9959101631, 1995.

Barzaghi, P., and Herrmann, H.: Kinetics and mechanisms of reactions of the nitrate radical (NO_3) with substituted phenols in aqueous solution, Phys. Chem. Chem. Phys., 6, 5379-5388, https://doi.org/10.1039/b412933d, 2004.

Becker, M.: Über magnetische Kernresonanzspektren wäßriger Brenztraubensäurelösungen, Ber. Bunsenges. Phys. Chem., 68, 669-676, https://doi.org/10.1002/bbpc.19640680713, 1964.

Bell, R. P.: The Reversible Hydration of Carbonyl Compounds, in: Advances in Physical Organic Chemistry Volume 4, edited by: Gold, V., Advances in Physical Organic Chemistry, Academic Press, 1-29, https://doi.org/10.1016/s0065-3160(08)60351-2, 1966.

Beltran, F. J., Rodriguez, E. M., and Romero, M. T.: Kinetics of the ozonation of muconic acid in water, J. Hazard Mater., 138, 534-538, https://doi.org/10.1016/j.jhazmat.2006.05.089, 2006.

Beltrán, F. J., García-Araya, J. F., Rivas, F. J., Alvarez, P., and Rodríguez, E.: Kinetics of competitive ozonation of some phenolic compounds present in wastewater from food processing industries, Ozone Sci. Eng., 22, 167-183, https://doi.org/0.1080/01919510008547218, 2000.

Biswal, J., Paul, J., Naik, D. B., Sarkar, S. K., and Sabharwal, S.: Radiolytic degradation of 4-nitrophenol in aqueous solutions: Pulse and steady state radiolysis study, Radiat. Phys. Chem., 85, 161-166, https://doi.org/10.1016/j.radphyschem.2013.01.003, 2013.

Bjerrum, J., Schwarzenbach, G., and Sillen, L. G.: Stability Constants. Part II. Inorganic Ligands, Chemical Society, London, 1958.

Bonin, J., Janik, I., Janik, D., and Bartels, D. M.: Reaction of the hydroxyl radical with phenol in water up to supercritical conditions, J. Phys. Chem. A, 111, 1869-1878, https://doi.org/10.1021/jp0665325, 2007.

Braude, E. A., Nachod, F. C., and Philips, W. D.: Determination of Organic Structures by Physical Methods, Academic Press, New York, https://doi.org/10.1016/c2013-0-12413-0, 1955.

Bräuer, P.: Extension and application of a tropospheric aqueous phase chemical mechanism (CAPRAM) for aerosol and cloud models, PhD thesis, University of Leipzig, 2015.

Bräuer, P., Mouchel-Vallon, C., Tilgner, A., Mutzel, A., Böge, O., Rodigast, M., Poulain, L., van Pinxteren, D., Wolke, R., Aumont, B., and Herrmann, H.: Development of a protocol for the auto-generation of explicit aqueous-phase oxidation schemes of organic compounds, Atmos. Chem. Phys., 19, 9209-9239, https://doi.org/10.5194/acp-19-9209-2019, 2019.

Burkholder, J. B., Sander, S. P., Abbatt, J., Barker, J. R., Huie, R. E., Kolb, C. E., Kurylo, M. J., Orkin, V. L., Wilmouth, D. M., and Wine, P. H.: Chemical kinetics and photochemical data for use in atmospheric studies, Evaluation No. 18, JPL Publication 15-10, Jet Propulsion Laboratory, Pasadena, available at: http://jpldataeval.jpl.nasa.gov (last access: 27 Nov 2019), 2015.

Buschmann, H.-J., Dutkiewicz, E., and Knoche, W.: The Reversible Hydration of Carbonyl Compounds in Aqueous Solution Part II: The Kinetics of the Keto/Gem-diol Transition, Ber. Bunsenges. Phys. Chem., 86, 129-134, https://doi.org/10.1002/bbpc.19820860208, 1982.

Buxton, G. V., Greenstock, C. L., Helman, W. P., and Ross, A. B.: Critical Review of rate constants for reactions of hydrated electrons, hydrogen atoms and hydroxyl radicals (·OH/·O− in Aqueous Solution, J. Phys. Chem. Ref. Data, 17, 513-886, https://doi.org/10.1063/1.555805, 1988.

Canonica, S., Jans, U., Stemmler, K., and Hoigne, J.: Transformation kinetics of phenols in water: photosensitization by dissolved natural organic material and aromatic ketones, Environ. Sci. Technol., 29, 1822-1831, https://doi.org/10.1021/es00007a020, 1995.

Canonica, S.: Oxidation of Aquatic Organic Contaminants Induced by Excited Triplet States, CHIMIA, 61, 641-644, https://doi.org/10.2533/chimia.2007.641, 2007.

Chen, X., and Schuler, R. H.: Directing effects of phenyl substitution in the reaction of hydroxyl radical with aromatics: the radiolytic hydroxylation of biphenyl, J. Phys. Chem., 97, 421-425, https://doi.org/10.1021/j100104a025, 1993.

Cheng, Y., Zheng, G., Wei, C., Mu, Q., Zheng, B., Wang, Z., Gao, M., Zhang, Q., He, K., Carmichael, G., Pöschl, U., and Su, H.: Reactive nitrogen chemistry in aerosol water as a source of sulfate during haze events in China, Sci. Adv., 2, e1601530, https://doi.org/10.1126/sciadv.1601530, 2016.

Chin, M., and Wine, P. H.: A temperature-dependent competitive kinetics study of the aqueous-phase reactions of OH radicals with formate, formic acid, acetate, acetic acid and hydrated formaldeheyde, in: Aquatic and Surface Photochemistry, edited by: Helz, G. R., Zepp, R. G., and Crosby, D. G., Lewis, Boca Raton, 85-96, 1994.

Christie, A. O., and Crisp, D. J.: Activity coefficients of the n-primary, secondary and tertiary aliphatic amines in aqueous solution, J. Appl. Chem., 17, 11-14, https://doi.org/10.1002/jctb.5010170103, 1967.

Clifton, C. L., Altstein, N., and Huie, R. E.: Rate constant for the reaction of nitrogen dioxide with sulfur(IV) over the pH range 5.3-13, Environ. Sci. Technol., 22, 586-589, https://doi.org/10.1021/es00170a018, 1988.

Cooper, A. J. L., and Redfield, A. G.: Proton magnetic resonance studies of alpha-keto acids, J. Biol. Chem., 250, 527-532, https://doi.org/http://www.jbc.org/content/250/2/527.full.pdf, 1975.

Copper, A. J., and Redfield, A. G.: Proton magnetic resonance studies of alpha-keto acids., J. Biol. Chem., 250, 527-532, 1975.

Creighton, D. J., Migliorini, M., Pourmotabbed, T., and Guha, M. K.: Optimization of efficiency in the glyoxalase pathway, Biochem., 27, 7376-7384, https://doi.org/10.1021/bi00419a031, 1988.

de Semainville, P., Hoffmann, D., George, C., and Herrmann, H.: Study of nitrate radical (NO_3) reactions with carbonyls and acids in aqueous solution as a function of temperature, Phys. Chem. Chem. Phys., 9, 958-968, https://doi.org/10.1039/b613956f, 2007.

de Sémainville, P. G., D Anna, B., and George, C.: Aqueous Phase Reactivity of Nitrate Radicals (NO_3) Toward Dicarboxylic Acids, Z. Phys. Chem., 224, 1247-1260, https://doi.org/10.1524/zpch.2010.6150, 2010.

Deborde, M., Rabouan, S., Duguet, J. P., and Legube, B.: Kinetics of aqueous ozone-induced oxidation of some endocrine disruptors, Environ. Sci. Technol., 39, 6086-6092, https://doi.org/10.1021/es0501619, 2005.

Doussin, J. F., and Monod, A.: Structure–activity relationship for the estimation of OH-oxidation rate constants of carbonyl compounds in the aqueous phase, Atmos. Chem. Phys., 13, 11625-11641, https://doi.org/10.5194/acp-13-11625-2013, 2013.

Dwibedy, P., Dey, G. R., Naik, D. B., Kishore, K., and Moorthy, P. N.: Pulse radiolysis studies on redox reactions of gallic acid: one electron oxidation of gallic acid by gallic acid–OH adduct, Phys. Chem. Chem. Phys., 1, 1915-1918, https://doi.org/10.1039/a809147a, 1999.

Elliot, A. J., and Simsons, A. S.: Rate constants for reactions of hydroxyl radicals as a function of temperature, Radiat. Phys. Chem., 24, 229-231, https://doi.org/10.1016/0146-5724(84)90056-6, 1984.

Erickson, P. R., Moor, K. J., Werner, J. J., Latch, D. E., Arnold, W. A., and McNeill, K.: Singlet Oxygen Phosphorescence as a Probe for Triplet-State Dissolved Organic Matter Reactivity, Environ. Sci. Technol., 52, 9170-9178, https://doi.org/10.1021/acs.est.8b02379, 2018.

Ervens, B., Gligorovski, S., and Herrmann, H.: Temperature-dependent rate constants for hydroxyl radical reactions with organic compounds in aqueous solutions, Phys. Chem. Chem. Phys., 5, 1811-1824, https://doi.org/10.1039/b300072a, 2003.

Exner, M., Herrmann, H., and Zellner, R.: Rate constants for the reactions of the NO_3 radical with HCOOH/HCOO? and CH_3COOH/CH_3COO? in aqueous solution between 278 and 328 K, J. Atmos. Chem., 18, 359-378, https://doi.org/10.1007/bf00712451, 1994.

Felber, T., Schaefer, T., and Herrmann, H.: OH-Initiated Oxidation of Imidazoles in Tropospheric Aqueous-Phase Chemistry, J. Phys. Chem. A, 123, 1505-1513, https://doi.org/10.1021/acs.jpca.8b11636, 2019.

Felber, T., Schaefer, T., and Herrmann, H.: Five-Membered Heterocycles as Potential Photosensitizers in the Tropospheric Aqueous Phase: Photophysical Properties of Imidazole-2-carboxaldehyde, 2-Furaldehyde, and 2-Acetylfuran, J. Phys. Chem. A, 124, 10029-10039, https://doi.org/10.1021/acs.jpca.0c07028, 2020.

Felber, T., Schaefer, T., He, L., and Herrmann, H.: Aromatic Carbonyl and Nitro Compounds as Photosensitizers and Their Photophysical Properties in the Tropospheric Aqueous Phase, J. Phys. Chem. A, 125, 5078-5095, https://doi.org/10.1021/acs.jpca.1c03503, 2021.

Fischer, G., Flatau, S., Schellenberger, A., and Zschunke, A.: Carbon-13 NMR investigations on the structure of .alpha.-keto acids in aqueous solution, J. Org. Chem., 53, 214-216, https://doi.org/10.1021/jo00236a050, 1988.

Fischer, M., and Warneck, P.: The Dissociation Constant of Pyruvic Acid: Determination by Spectrophotometric Measurements, Ber. Bunsenges. Phys. Chem., 95, 523-527, https://doi.org/10.1002/bbpc.19910950414, 1991.

Fleury, M. B., Moiroux, J., Fleury, D., and Dufresne, J.-C.: Consequences of acceptor effects on the polarization of oxo and imine bonds in acyl and R-CO-COOH compounds, J. Electroanal. Chem., 81, 365-376, https://doi.org/10.1016/s0022-0728(77)80033-8, 1977.

Fonds, A. W., Molenaar, J. L., and Los, J. M.: Pulse polarography, J. Electroanal. Chem., 22, 229-241, https://doi.org/10.1016/s0022-0728(69)80255-x, 1969.

Geeta, S., Sharma, S. B., Rao, B. S. M., Mohan, H., Dhanya, S., and Mittal, J. P.: Study of kinetics and absorption spectra of OH adducts of hydroxy derivatives of benzaldehyde and acetophenone, J. Photoch. Photobio. A, 140, 99-107, https://doi.org/10.1016/s1010-6030(01)00402-6, 2001.

Gligorovski, S., Rousse, D., George, C. H., and Herrmann, H.: Rate constants for the OH reactions with oxygenated organic compounds in aqueous solution, Int. J. Chem. Kinet., 41, 309-326, https://doi.org/10.1002/kin.20405, 2009.

Gold, V., Socrates, G., and Crampton, M.: Hydration of pyruvic acid, J. Chem. Soc., S, 5888-5889, 1964.

Gomez-Bombarelli, R., Gonzalez-Perez, M., Perez-Prior, M. T., Calle, E., and Casado, J.: Computational calculation of equilibrium constants: addition to carbonyl compounds, J. Phys. Chem. A, 113, 11423-11428, https://doi.org/10.1021/jp907209a, 2009.

Greenzaid, P., Luz, Z., and Samuel, D.: A Nuclear Magnetic Resonance Study of the Reversible Hydration of Aliphatic Aldehydes and Ketones. I. Oxygen-17 and Proton Spectra and Equilibrium Constants, J. Am. Chem. Soc., 89, 749-756, https://doi.org/10.1021/ja00980a004, 1967a.

Greenzaid, P., Luz, Z., and Samuel, D.: A Nuclear Magnetic Resonance Study of the Reversible Hydration of Aliphatic Aldehydes and Ketones. II. The Acid-Catalyzed Oxygen Exchange of Acetaldehyde, J. Am. Chem. Soc., 89, 756-759, https://doi.org/10.1021/ja00980a005, 1967b.

Griffiths, V. S., and Socrates, G.: N.m.r. study of the hydration of pyruvic acid, Trans. Faraday Soc., 63, 673-677, https://doi.org/10.1039/TF9676300673, 1967.

Gunshore, S., Brush, E. J., and Hamilton, G. A.: Equilibrium constants for the formation of glyoxylate thiohemiacetals and kinetic constants for their oxidation by O2 catalyzed by l-hydroxy acid oxidase, Bioorg. Chem., 13, 1-13, https://doi.org/10.1016/0045-2068(85)90002-1, 1985.

Guo, X. X., and Brimblecombe, P.: Henry's law constants of phenol and mononitrophenols in water and aqueous sulfuric acid, Chemosphere, 68, 436-444, https://doi.org/10.1016/j.chemosphere.2007.01.011, 2007.

Guthrie, J. P.: Acetopyruvic acid. Rate and equilibrium constants for hydration and enolization, J. Am. Chem. Soc., 94, 7020-7024, https://doi.org/10.1021/ja00775a026, 1972.

Guthrie, J. P.: Hydration of Carbonyl Compounds, an Analysis in Terms of Multidimensional Marcus Theory, J. Am. Chem. Soc., 122, 5529-5538, https://doi.org/10.1021/ja992992i, 2000.

Haynes, W. M.: CRC Handbook of Chemistry and Physics, 97 ed., Taylor and Francis, CRC Press, Boca Raton, FL, 1958.

Hellström, N., and Almqvist, S.-O.: Hydration and dehydration of pyruvic acid, J. Phys. Chem. B, 0, 1396-1400, https://doi.org/10.1039/j29700001396, 1970.
Herrmann, H.: Kinetics of aqueous phase reactions relevant for atmospheric chemistry, Chem. Rev., 103, 4691-4716, https://doi.org/10.1021/cr020658q, 2003.
Hesper, J.: Spektroskopische und kinetische Untersuchungen von Reaktionen der Radikale O_2^- und OH in wässriger Phase, University of Leipzig, Leipzig, Germany, 2003.
Hilal, S. H., Bornander, L. L., and Carreira, L. A.: Hydration Equilibrium Constants of Aldehydes, Ketones and Quinazolines, QSAR Comb. Sci., 24, 631-638, https://doi.org/10.1002/qsar.200430913, 2005.
Hoffmann, E. H., Schrödner, R., Tilgner, A., Wolke, R., and Herrmann, H.: CAPRAM reduction towards an operational multiphase halogen and dimethyl sulfide chemistry treatment in the chemistry transport model COSMO-MUSCAT(5.04e), Geosci. Model Dev., 13, 2587-2609, https://doi.org/10.5194/gmd-13-2587-2020, 2020.
Hoigné, J., and Bader, H.: Rate constants of reactions of ozone with organic and inorganic compounds in water—II, Water Res., 17, 185-194, https://doi.org/10.1016/0043-1354(83)90099-4, 1983.
Ip, H. S. S., Huang, X. H. H., and Yu, J. Z.: Effective Henry's law constants of glyoxal, glyoxylic acid, and glycolic acid, Geophys. Res. Lett., 36, L01802, https://doi.org/10.1029/2008gl036212, 2009.
Jans, U.: Radikalbildung aus Ozon in atmosphärischen Wassern-Einfluss von Licht, gelösten Stoffen und Rußpartikeln, Radikalbildung aus Ozon in atmosphärischen Wassern-Einfluss von Licht, gelösten Stoffen und Rußpartikeln, PhD Dissertation, ETH Zürich, Switzerland, 1996.
Jen, J., and Knoche, W.: Alpha-ketoglutaric acid hydration, Ber. Bunsenges. Phys. Chem., 73, 539-541, https://doi.org/10.1002/bbpc.19690730607, 1969.
Jencks, W. P., and Carriuolo, J.: Structure of Pyruvate in Aqueous Solution, 182, 598-599, https://doi.org/10.1038/182598b0, 1958.
Kaur, R., and Anastasio, C.: First Measurements of Organic Triplet Excited States in Atmospheric Waters, Environ. Sci. Technol., 52, 5218-5226, https://doi.org/10.1021/acs.est.7b06699, 2018.
Kerber, R. C., and Fernando, M. S.: α-Oxocarboxylic Acids, J. Chem. Educ., 87, 1079-1084, https://doi.org/10.1021/ed1003096, 2010.
Khan, I., Brmblecombe, P., and Clegg, S. L.: The henry's law constants of pyruvic and methacrylic acids, Environ. Technol., 13, 587-593, https://doi.org/10.1080/09593339209385187, 1992.
King, M. D., Thompson, K. C., Ward, A. D., Pfrang, C., and Hughes, B. R.: Oxidation of biogenic and water-soluble compounds in aqueous and organic aerosol droplets by ozone: a kinetic and product analysis approach using laser Raman tweezers, Faraday discuss., 137, 173-192, https://doi.org/10.1039/b702199b, 2008.
Knoche, W., Arturo Lopez-Quintela, M., and Weiffen, J.: The Hydration of Aliphatic Aldehydes and Pyruvic Acid in Mixed Solvents, Ber. Bunsenges. Phys. Chem., 89, 1047-1050, https://doi.org/10.1002/bbpc.19850891008, 1985.
Kokesh, F. C.: The determination by proton nuclear magnetic resonance of the enol, hydrate and keto forms of oxaloacetic acid and its anions, J. Org. Chem., 41, 3593-3599, https://doi.org/10.1021/jo00884a025, 1976.
Kolthoff, I. M., and Elving, P. J.: Treatise on analytical chemistry, Interscience Encyclopedia, Inc., New York, 1959.
Kozlowski, J., and Zuman, P.: Polarographic reduction of aldehydes and ketones, J. Electroanal. Chem., 226, 69-102, https://doi.org/10.1016/0022-0728(87)80035-9, 1987.
Kozlowski, J., and Zuman, P.: Acid-base, hydration-dehydration and keto-enol equilibria in aqueous solutions of α-ketoacids: Study by spectroscopy, polarography and linear sweep voltammetry, Bioelectroch. Bioener., 28, 43-70, https://doi.org/10.1016/0302-4598(92)80003-y, 1992.
Kraljic, I.: Kinetics of OH radical reactions in radiolysis, photolysis, and the Fenton system, in: The Chemistry of Ionization and Excitation, edited by: Johnson, G. R. A., and Scholes, G., Taylor and Francis Ltd, London, 303-309, 1967.
Kumar, M., Rao, M. H., Moorthy, P. N., and Rao, K. N.: Structural influences on the reactivity of vinyl monomers with hydroxyl radicals and hydrated electrons studied by pulse radiolysis, Radiat. Eff. Express, 1, 167 - 173, 1988.
Kumar, M., and Rao, M. H.: Pulse Radiolysis Study of Initiation, Dimerization, and Propagation Steps of 3,3-Dimethylacrylic Acid in Aqueous Medium, J. Macromol. Sci. A Chem., 28, 531-544, https://doi.org/10.1080/00222339108052105, 1991.
Kurz, J. L.: Hydration of acetaldehyde. I. Equilibrium thermodynamic parameters, J. Am. Chem. Soc., 89, 3524-3528, https://doi.org/10.1021/ja00990a032, 1967.

Kůta, J., and Valenta, P.: Ermittlung der Hydratationsgleichgewichtskonstanten der Glyoxalsäure und ihres Anions durch Oszillographie mit vorgegebenem Spannungsverlauf, Collection of Czech. Chem. Commun., 28, 1593-1597, https://doi.org/10.1135/cccc19631593, 1963.

Le Henaff, P.: Methodes d'etude et proprietes des hydrates, hemiacetals et hemithioacetals derives des aldehydes et des cetones., B. Soc. Chim. Fr., 4687–4698, 1968.

Lee, Y.-N., and Schwartz, S. E.: Kinetics of Oxidation of Aqueous Sulfur(IV) by Nitrogen Dioxide, in: Precipitation Scavenging, Dry Deposition, and Resuspension. Volume 1: Precipitation Scavenging, edited by: Pruppacher, H. R., Semonin, R. G., and Slinn, W. G., Elsevier, New York, Amsterdam, Oxford, 453-470, 1983.

Leitzke, A., Reisz, E., Flyunt, R., and von Sonntag, C.: The reactions of ozone with cinnamic acids: formation and decay of 2-hydroperoxy-2-hydroxyacetic acid, J. Chem. Soc., Perkin Trans. 2, 793-797, https://doi.org/10.1039/b009327k, 2001.

Leitzke, A., and von Sonntag, C.: Ozonolysis of unsaturated acids in aqueous solution: acrylic, methacrylic, maleic, fumaric and muconic acids, Ozone Sci. Eng., 31, 301-308, https://doi.org/0.1080/01919510903041354, 2009.

Leussing, D., and Stanfield, C. K.: A Nuclear Magnetic Resonance Study of Aqueous Pyruvate-Glycinate-Zinc(II) and Related Systems, J. Am. Chem. Soc., 86, 2805-2810, https://doi.org/10.1021/ja01068a011, 1964.

Leussing, D. L., and Hanna, E. M.: Metal Ion Catalysis in Transamination. IV. Nickel(II)-and Zinc(II)-Glyoxalate-Amino Acid Complexes1, J. Am. Chem. Soc., 88, 696-699, https://doi.org/10.1021/ja00956a014, 1966.

Lewis, C. A., and Wolfenden, R.: Influence of pressure on the equilibrium of hydration of aliphatic aldehydes, J. Am. Chem. Soc., 95, 6685-6688, https://doi.org/10.1021/ja00801a026, 1973.

Lienhard, G. E., and Jencks, W. P.: Thiol addition to the carbonyl group. Equilibria and kinetics, J. Am. Chem. Soc., 88, 3982-3994, https://doi.org/10.1021/ja00969a017, 1966.

Lilie, J., and Henglein, A.: Pulsradiolytische Untersuchung der Oxidation von ungesättigten Karbonylverbindungen in wäßriger Lösung: Hydratisierte Enole als Zwischenprodukte, Ber. Bunsenges. Phys. Chem., 74, 388-393, https://doi.org/10.1002/bbpc.19700740417, 1970.

Loeff, I., Treinin, A., and Linschitz, H.: Interactions of nitrite and sulfite with organic triplets: charge transfer versus energy transfer: the role of reorganization energy in triplet-anion interactions and spectroscopic methods for its evaluation, J. Phys. Chem., 96, 5264-5272, https://doi.org/10.1021/j100192a018, 1992.

Loeff, I., Rabani, J., Treinin, A., and Linschitz, H.: Charge transfer and reactivity of n.pi.* and .pi..pi.* organic triplets, including anthraquinonesulfonates, in interactions with inorganic anions: a comparative study based on classical Marcus theory, J. Am. Chem. Soc., 115, 8933-8942, https://doi.org/10.1021/ja00073a007, 1993.

Logan, S. R.: Redox reactions of organic radicals with ferrocene/ferricenium species in aqueous solution. Part 1. Radicals derived from carboxylic acids, J. Chem. Soc., Perkin Trans. 2, 751-754, https://doi.org/10.1039/p29890000751, 1989.

Lopalco, A., Douglas, J., Denora, N., and Stella, V. J.: Determination of pKa and Hydration Constants for a Series of alpha-Keto-Carboxylic Acids Using Nuclear Magnetic Resonance Spectrometry, J. Pharm. Sci., 105, 664-672, https://doi.org/10.1002/jps.24539, 2016.

Martin, L. R., Mezyk, S. P., and Mincher, B. J.: Determination of arrhenius and thermodynamic parameters for the aqueous reaction of the hydroxyl radical with lactic acid, J. Phys. Chem. A, 113, 141-145, https://doi.org/10.1021/jp806290s, 2009.

Maruthamuthu, M., and Dhandavel, R.: Binding of Bromocresol Green onto Poly(N-Vinyl-2-Pyrrolidone), Makromol. Chem. Rapid. Comm., 1, 633-636, https://doi.org/10.1002/marc.1980.030011007, 1980.

Maruthamuthu, P.: Absolute Rate Constants for the Reactions of Sulfate, Phosphate and Hydroxyl Radicals with Monomers, Makromol. Chem. Rapid. Comm., 1, 23-25, https://doi.org/10.1002/marc.1980.030010105, 1980.

McDonald, R. S., and Martin, E. V.: The kinetics and equilibrium of the hydration of phthalaldehyde, Can. J. Chem., 57, 506-516, https://doi.org/10.1139/v79-084, 1979.

Merz, J. H., and Waters, W. A.: S3. Some oxidations involving the free hydroxyl radical, J. Chem. Soc., S15-S25, https://doi.org/10.1039/jr9490000s15, 1949.

Montalti, M., Credi, A., Prodi, L., and Gandolfi, M. T.: Handbook of Photochemistry (3rd ed.), CRC Press., https://doi.org/10.1201/9781420015195, 2006.

Montoya, M. R., and Mellado, J. M. R.: Use of convolutive potential sweep voltammetry in the calculation of hydration equilibrium constants of α-dicarbonyl compounds, J. Electroanal. Chem., 370, 183-187, https://doi.org/10.1016/0022-0728(93)03203-2, 1994.

Moor, K. J., Schmitt, M., Erickson, P. R., and McNeill, K.: Sorbic Acid as a Triplet Probe: Triplet Energy and Reactivity with Triplet-State Dissolved Organic Matter via (1)O2 Phosphorescence, Environ. Sci. Technol., 53, 8078-8086, https://doi.org/10.1021/acs.est.9b01787, 2019.

Moore, J. S., Kemsley, K. G., Davies, J. V., and Phillips, G. O.: Pulse radiolysis of carbohydrates, in: Radiation Biology and Chemistry. Research Developements (Proceedings of the Association for Radiation Research), edited by: Edwards, H. E., Navaratnam, S., Parsons, B. J., and Phillips, G. O., Elsevier, New York, 99-113, 1979.

Mvula, E., and von Sonntag, C.: Ozonolysis of phenols in aqueous solution, Org. Biomol. Chem., 1, 1749-1756, https://doi.org/10.1039/b301824p, 2003.

Nauser, T., and Bühler, R. E.: Pivalic acid as combined buffer and scavenger for studies of cloud water chemistry with pulse radiolysis, J. Chem. Soc., Faraday Trans., 90, 3651-3656, https://doi.org/10.1039/ft9949003651, 1994.

Neta, P., and Dorfman, L. M.: Pulse Radiolysis Studies. XIII. Rate Constants for Reaction of Hydroxyl Radicals with Aromatic Compounds in Aqueous Solutions, Adv. Chem. Ser., 222-230, https://doi.org/0.1021/ba-1968-0081.ch015, 1968.

Ning, B., Graham, N. J., and Zhang, Y.: Degradation of octylphenol and nonylphenol by ozone - part I: direct reaction, Chemosphere, 68, 1163-1172, https://doi.org/10.1016/j.chemosphere.2007.01.055, 2007.

O'Neill, P., Steenken, S., and Schulte-Frohlinde, D.: Formation of radical zwitterions from methoxylated benzoic acids. 2. Hydroxyl adducts as precursors, J. Phys. Chem., 81, 31-34, https://doi.org/10.1021/j100516a008, 1977.

Ogata, Y., and Kawasaki, A.: Equilibrium additions to carbonyl compounds, in: The Chemistry of the Carbonyl Group, edited by: Zabicky, J., PATAI'S Chemistry of Functional Groups, Interscience Publishers, London, New York, Sydney, Toronto, 2-59, https://doi.org/10.1002/9780470771228.ch1, 1970.

Öjelund, G., Wadsö, I., Thom, E., Stoll, E., Eriksson, G., Blinc, R., Paušak, S., Ehrenberg, L., and Dumanović, J.: Thermochemistry of Ionization Reactions for Glyoxylic Acid, Pyruvic Acid, and alpha-Ketobutyric Acid, Acta Chem. Scand., 21, 1408-1414, https://doi.org/10.3891/acta.chem.scand.21-1408, 1967.

Ossola, R., Schmitt, M., Erickson, P. R., and McNeill, K.: Furan Carboxamides as Model Compounds To Study the Competition between Two Modes of Indirect Photochemistry, Environ. Sci. Technol., 53, 9594-9603, https://doi.org/10.1021/acs.est.9b02895, 2019.

Otto, T., Schaefer, T., and Herrmann, H.: Aqueous-Phase Oxidation of Terpene-Derived Acids by Atmospherically Relevant Radicals, J. Phys. Chem. A, 122, 9233-9241, https://doi.org/10.1021/acs.jpca.8b08922, 2018.

Park, J. Y., and Lee, Y. N.: Solubility and decomposition kinetics of nitrous acid in aqueous solution, J. Phys. Chem., 92, 6294-6302, https://doi.org/10.1021/j100333a025, 1988.

Patting, H., and Strehlow, H.: Pyruvic acid hydration, Ber. Bunsenges. Phys. Chem., 73, 534-538, https://doi.org/10.1002/bbpc.19690730606, 1969.

Pocker, Y., Meany, J. E., Nist, B. J., and Zadorojny, C.: Reversible hydration of pyruvic acid. I. Equilibrium studies, J. Phys. Chem., 73, 2879-2882, https://doi.org/10.1021/j100843a015, 1969.

Poznyak, T., and Vivero, J.: Degradation of aqueous phenol and chlorinated phenols by ozone, Ozone Sci. Eng., 27, 447-458, https://doi.org/0.1080/01919510500351529, 2005.

Raabe, G.: Eine laserphotolytische Studie zur Kinetik von Raktionen des NO_3^- Radikals in wäßriger Lösung, Essen, 1996.

Ramseier, M. K., and Gunten, U. v.: Mechanisms of Phenol Ozonation—Kinetics of Formation of Primary and Secondary Reaction Products, Ozone Sci. Eng., 31, 201-215, https://doi.org/10.1080/01919510902740477, 2009.

Rapf, R. J., Dooley, M. R., Kappes, K., Perkins, R. J., and Vaida, V.: pH Dependence of the Aqueous Photochemistry of alpha-Keto Acids, J. Phys. Chem. A, 121, 8368-8379, https://doi.org/10.1021/acs.jpca.7b08192, 2017a.

Rapf, R. J., Perkins, R. J., Carpenter, B. K., and Vaida, V.: Mechanistic Description of Photochemical Oligomer Formation from Aqueous Pyruvic Acid, J. Phys. Chem. A, 121, 4272-4282, https://doi.org/10.1021/acs.jpca.7b03310, 2017b.

Rappoport, Z.: CRC Handbook of Tables for Organic Compound Identification, Taylor and Francis, CRC Press, Boca Raton, FL, 1984.

Raventos-Duran, T., Camredon, M., Valorso, R., Mouchel-Vallon, C., and Aumont, B.: Structure-activity relationships to estimate the effective Henry's law constants of organics of atmospheric interest, Atmos. Chem. Phys., 10, 7643-7654, https://doi.org/10.5194/acp-10-7643-2010, 2010.

Reed Harris, A. E., Ervens, B., Shoemaker, R. K., Kroll, J. A., Rapf, R. J., Griffith, E. C., Monod, A., and Vaida, V.: Photochemical kinetics of pyruvic acid in aqueous solution, J. Phys. Chem. A, 118, 8505-8516, https://doi.org/10.1021/jp502186q, 2014.

Rendina, A. R., Hermes, J. D., and Cleland, W. W.: A novel method for determining rate constants for dehydration of aldehyde hydrates, Biochem., 23, 5148-5156, https://doi.org/10.1021/bi00317a011, 1984.
Rivlin, M., Eliav, U., and Navon, G.: NMR studies of the equilibria and reaction rates in aqueous solutions of formaldehyde, J. Phys. Chem. B, 119, 4479-4487, https://doi.org/10.1021/jp513020y, 2015.
Ross, A. B., Bielski, B. H. J., Buxton, G. V., Cabelli, D. E., Helman, W. P., Huie, R. E., Grodkowski, J., Neta, P., Mulazzani, Q. G., and Wilkinson, F.: NIST Standard Reference Database 40, Version 3.0, Gaithersburg, MD, USA, 1998.
Sander, R.: Compilation of Henry's law constants (version 4.0) for water as solvent, Atmos. Chem. Phys., 15, 4399-4981, https://doi.org/10.5194/acp-15-4399-2015, 2015.
Savel'eva, O. S., Vysotska.Na, and Shevchuk, L. G.: Reactions of Substituted Phenols with Hydroxyl Radicals and Their Dissociated Form O^-, Zh. Org. Khim., 8, 283-, 1972.
Scholes, G., and Willson, R. L.: Gamma-Radiolysis of aqueous thymine solutions. Determination of relative reaction rates of OH radicals, Trans. Faraday Soc., 63, 2983-2993 https://doi.org/10.1039/tf9676302983, 1967.
Schöne, L., and Herrmann, H.: Kinetic measurements on the reactivity of hydrogen peroxide and ozone towards small atmospherically relevant aldehydes, ketones and organic acids in aqueous solution, Atmos. Chem. Phys., 14, 4503-4514, https://doi.org/0.5194/acp-14-4503-2014, 2014.
Schöne, L., Schindelka, J., Szeremeta, E., Schaefer, T., Hoffmann, D., Rudzinski, K. J., Szmigielski, R., and Herrmann, H.: Atmospheric aqueous phase radical chemistry of the isoprene oxidation products methacrolein, methyl vinyl ketone, methacrylic acid and acrylic acid--kinetics and product studies, Phys Chem Chem Phys, 16, 6257-6572, https://doi.org/10.1039/c3cp54859g, 2014.
Schuchmann, M. N., Schuchmann, H. P., and Von Sonntag, C.: Oxidation of Hydroxymalonic Acid by OH Radicals in the Presence and in the Absence of Molecular Oxygen - A Pulse-Radiolysis and Product Study, J. Phys. Chem., 99, 9122-9129, https://doi.org/0.1021/j100022a026, 1995.
Schweitzer, C., and Schmidt, R.: Physical mechanisms of generation and deactivation of singlet oxygen, Chem. Rev., 103, 1685-1757, https://doi.org/10.1021/cr010371d, 2003.
Sehested, K., Getoff, N., Schwoerer, F., Markovic, V. M., and Nielsen, S. O.: Pulse radiolysis of oxalic acid and oxalates, J. Phys. Chem., 75, 749-755, https://doi.org/10.1021/j100676a004, 1971.
Sharpless, C. M.: Lifetimes of triplet dissolved natural organic matter (DOM) and the effect of NaBH(4) reduction on singlet oxygen quantum yields: implications for DOM photophysics, Environ. Sci. Technol., 46, 4466-4473, https://doi.org/10.1021/es300217h, 2012.
Shetiya, R. S., Rao, K. N., and Shankar, J.: OH radical rate constants of phenols using para-nitrosodimethylaniline, INDIAN J. Chem. A, 14, 575-578, 1976.
Socrates, G.: Kinetic study by NMR, J. Chem. Educ., 44, 575-&, https://doi.org/10.1021/ed044p575, 1967.
Socrates, G.: Hydration study of acetaldehyde and propionaldehyde, J. Org. Chem., 34, 2958-2961, https://doi.org/10.1021/jo01262a033, 1969.
Sorensen, P. E., and Jencks, W. P.: Acid- and base-catalyzed decomposition of acetaldehyde hydrate and hemiacetals in aqueous solution, J. Am. Chem. Soc., 109, 4675-4690, https://doi.org/10.1021/ja00249a034, 1987.
Sørensen, P. E., Bruhn, K., and Lindeløv, F.: Kinetics and Equilibria for the Reversible Hydration of the Aldehyde Group in Glyoxylic Acid., Acta Chem. Scand., 28A, 162-168, https://doi.org/10.3891/acta.chem.scand.28a-0162, 1974.
Sørensen, P. E., and Jencks, W. P.: Acid- and base-catalyzed decomposition of acetaldehyde hydrate and hemiacetals in aqueous solution, J. Am. Chem. Soc., 109, 4675-4690, https://doi.org/10.1021/ja00249a034, 1987.
Spindler, G., Hesper, J., Brüggemann, E., Dubois, R., Müller, T., and Herrmann, H.: Wet annular denuder measurements of nitrous acid: laboratory study of the artefact reaction of NO_2 with S(IV) in aqueous solution and comparison with field measurements, Atmos. Environ., 37, 2643-2662, https://doi.org/10.1016/S1352-2310(03)00209-7, 2003.
Strehlow, H.: Die Kininetic der Hydratation von alpha-Ketocarbonsäuren, Z. Elektrochem., 66, 392-396, https://doi.org/10.1002/bbpc.19620660503, 1962.
Sutton, H. C., and Downes, T. M.: Rate of hydration of formaldehyde in aqueous solution, J. Chem. Soc., Chem Comm., 1-2, https://doi.org/10.1039/C39720000001, 1972.
Tilgner, A., Bräuer, P., Wolke, R., and Herrmann, H.: Modelling multiphase chemistry in deliquescent aerosols and clouds using CAPRAM3.0i, J. Atmos. Chem., 70, 221-256, https://doi.org/10.1007/s10874-013-9267-4, 2013.

Tinel, L., Dumas, S., and George, C.: A time-resolved study of the multiphase chemistry of excited carbonyls: Imidazole-2-carboxaldehyde and halides, C. R. Chim., 17, 801-807, https://doi.org/10.1016/j.crci.2014.03.008, 2014.
TOXNET: (TOXicology data NET-work), HDSB: Haxardous Substances Data Bank, National Library of Medicine (US), 2015.
Treinin, A., and Hayon, E.: Quenching of triplet states by inorganic ions. Energy transfer and charge transfer mechanisms, J. Am. Chem. Soc., 98, 3884-3891, https://doi.org/10.1021/ja00429a025, 1976.
Tremp, J., Mattrel, P., Fingler, S., and Giger, W.: Phenols and nitrophenols as tropospheric pollutants: Emissions from automobile exhausts and phase transfer in the atmosphere, Water-Air-Soil-Poll., 68, 113-123, https://doi.org/10.1007/bf00479396, 1993.
Tur'yan, Y.: Kinetics and equilibrium of the dehydration-hydration and recombination-dissociation reactions of glyoxylic acid investigated by electrochemical methods, Croat. Chem. Acta, 71, 727-743, 1998.
Tur'yan, Y.: Studying kinetics of the dehydration reaction of pyruvic acid and ethyl pyruvate using the polarographic kinetic currents, Croat. Chem. Acta, 72, 13-24, 1999.
Tur'yan, Y.: Studying kinetics of the dehydration reaction of acetaldehyde in aqueous solutions using polarographic kinetic currents, Croat. Chem. Acta, 73, 657-666, 2000.
Tur'yan, Y. I.: Kinetics and Equilibrium of the Dehydration-Hydration and Recombination-Dissociation Reactions of Glyoxylic Acid Investigated by Electrochemical Methods, Croat. Chem. Acta, 71, 727 - 742, 1998.
Tur'yan, Y. I.: Studying Kinetics of the Dehydration Reaction of Pyruvic Acid and Ethyl Pyruvate Using the Polarographic Kinetic Currents, Croat. Chem. Acta, 72, 13-34, 1999.
Tur'yan, Y. I.: Studying Kinetics of the Dehydration Reaction of Acetaldehyde in Aqueous Solutions Using Polarographic Kinetic Currents, Croat. Chem. Acta, 73, 657-666, 2000.
Umschlag, T., Zellner, R., and Herrmann, H.: Laser-based studies of NO3 radical reactions with selected aromatic compounds in aqueous solution, Phys. Chem. Chem. Phys., 4, 2975-2982, https://doi.org/10.1039/b110263j, 2002.
Walling, C., and El-Taliawi, G. M.: Fenton's reagent. II. Reactions of carbonyl compounds and .alpha.,.beta.-unsaturated acids, J. Am. Chem. Soc., 95, 844-847, https://doi.org/10.1021/ja00784a035, 1973.
Wander, R., Neta, P., and Dorfman, L. M.: Pulse radiolysis studies. XII. Kinetics and spectra of the cyclohexadienyl radicals in aqueous benzoic acid solution, J. Phys. Chem., 72, 2946-2949, https://doi.org/10.1021/j100854a044, 1968.
Wang, X., Gemayel, R., Hayeck, N., Perrier, S., Charbonnel, N., Xu, C., Chen, H., Zhu, C., Zhang, L., Wang, L., Nizkorodov, S. A., Wang, X., Wang, Z., Wang, T., Mellouki, A., Riva, M., Chen, J., and George, C.: Atmospheric Photosensitization: A New Pathway for Sulfate Formation, Environ. Sci. Technol., 54, 3114-3120, https://doi.org/10.1021/acs.est.9b06347, 2020.
Warneck, P.: Multi-Phase Chemistry of C_2 and C_3 Organic Compounds in the Marine Atmosphere, J. Atmos. Chem., 51, 119-159, https://doi.org/10.1007/s10874-005-5984-7, 2005.
Wasa, T., and Musha, S.: Polarographic Behavior of Glyoxal and its Related Compounds, B. .Univ. Osaka Prefecture, 19, 169-180, 1970.
Weller, C., Hoffmann, D., Schaefer, T., and Herrmann, H.: Temperature and Ionic Strength Dependence of NO_3-radical Reactions with Substituted Phenols in Aqueous Solution, Z. Phys. Chem., 224, 1261-1287, https://doi.org/10.1524/zpch.2010.6151, 2010.
Wenk, J., Eustis, S. N., McNeill, K., and Canonica, S.: Quenching of excited triplet states by dissolved natural organic matter, Environ. Sci. Technol., 47, 12802-12810, https://doi.org/10.1021/es402668h, 2013.
Witkowski, B., Al-Sharafi, M., and Gierczak, T.: Kinetics of Limonene Secondary Organic Aerosol Oxidation in the Aqueous Phase, Environ. Sci. Technol., 52, 11583-11590, https://doi.org/10.1021/acs.est.8b02516, 2018a.
Witkowski, B., Jurdana, S., and Gierczak, T.: Limononic Acid Oxidation by Hydroxyl Radicals and Ozone in the Aqueous Phase, Environ. Sci. Technol., 52, 3402-3411, https://doi.org/10.1021/acs.est.7b04867, 2018b.
Witkowski, B., Al-Sharafi, M., and Gierczak, T.: Ozonolysis of beta-Caryophyllonic and Limononic Acids in the Aqueous Phase: Kinetics, Product Yield, and Mechanism, Environ. Sci. Technol., 53, 8823-8832, https://doi.org/10.1021/acs.est.9b02471, 2019.
Yang, X. K., Wang, J. Q., and Wang, T. D.: Rate constants for the reactions of $NO_3^{\cdot}$ and $SO_4^{\cdot-}$ radicals with oxalic acid and oxalate anions in aqueous solution, Chineses Chem. Lett., 15, 583-586, 2004.

Ye, C., Chen, H., Hoffmann, E. H., Mettke, P., Tilgner, A., He, L., Mutzel, A., Bruggemann, M., Poulain, L., Schaefer, T., Heinold, B., Ma, Z., Liu, P., Xue, C., Zhao, X., Zhang, C., Zhang, F., Sun, H., Li, Q., Wang, L., Yang, X., Wang, J., Liu, C., Xing, C., Mu, Y., Chen, J., and Herrmann, H.: Particle-Phase Photoreactions of HULIS and TMIs Establish a Strong Source of H_2O_2 and Particulate Sulfate in the Winter North China Plain, Environ. Sci. Technol., 55, 7818-7830, https://doi.org/10.1021/acs.est.1c00561, 2021.

Zavitsas, A. A., Coffiner, M., Wiseman, T., and Zavitsas, L. R.: Reversible hydration of formaldehyde. Thermodynamic parameters, J. Phys. Chem., 74, 2746-2750, https://doi.org/10.1021/j100708a003, 1970.

Zepp, R. G., Schlotzhauer, P. F., and Sink, R. M.: Photosensitized transformations involving electronic energy transfer in natural waters: role of humic substances, Environ. Sci. Technol., 19, 74-81, https://doi.org/10.1021/es00131a008, 1985.

Zepp, R. G., Faust, B. C., and Hoigne, J.: Hydroxyl Radical Formation in Aqueous Reactions (pH 3-8) of Iron(II) with Hydrogen-Peroxide - the Photo-Fenton Reaction, Environ. Sci. Technol., 26, 313-319, https://doi.org/0.1021/es00026a011, 1992.

Zirchrom: Dissociation constants of organic acids and bases. https://www.zirchrom.com/organic.htm, 2018.
